# Supplementary figures and images for: The coordinated action of UFMylation and the RQC pathways clears arrested polypeptides at the ER (part 1 of 2)
Source: EMBO J. 2026 Mar 25;45(9):3252–75. doi: 10.1038/s44318-026-00753-9 (PMC13144351; doi:10.1038/s44318-026-00753-9)

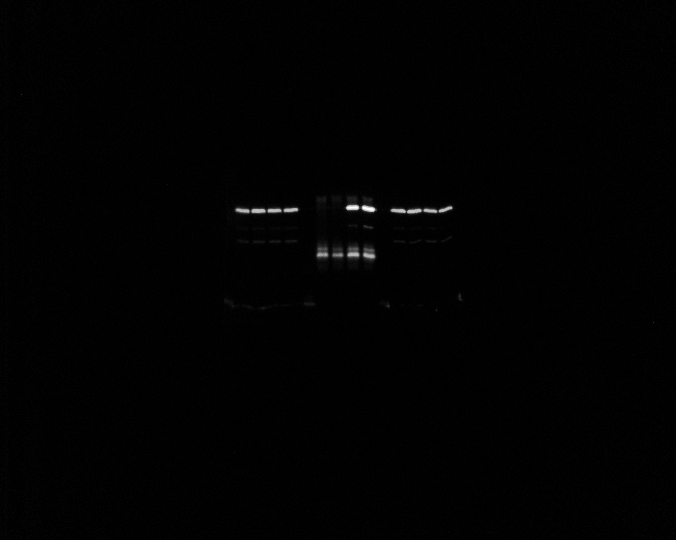

Supplement: Supplementary file 4 — Source data Fig. 1 [file 44318_2026_753_MOESM4_ESM.zip › Figure 1/1D/DDRGK1/CHEMI_10212025_184811Chemi.tif]

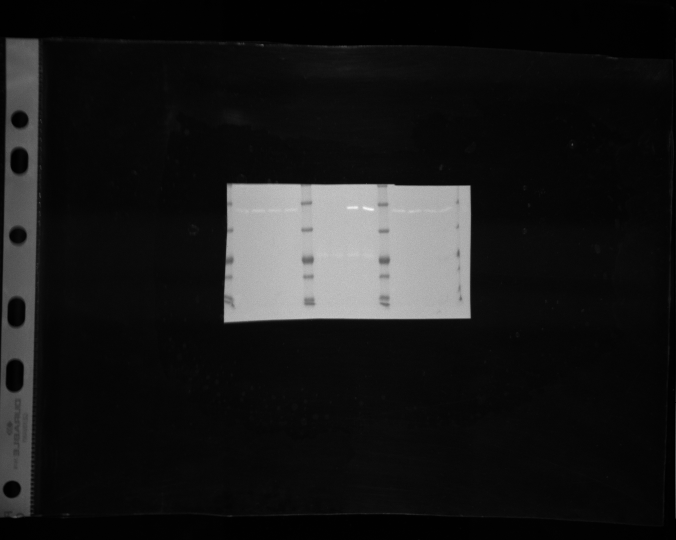

Supplement: Supplementary file 4 — Source data Fig. 1 [file 44318_2026_753_MOESM4_ESM.zip › Figure 1/1D/DDRGK1/CHEMI_10212025_184811Membrane.tif]

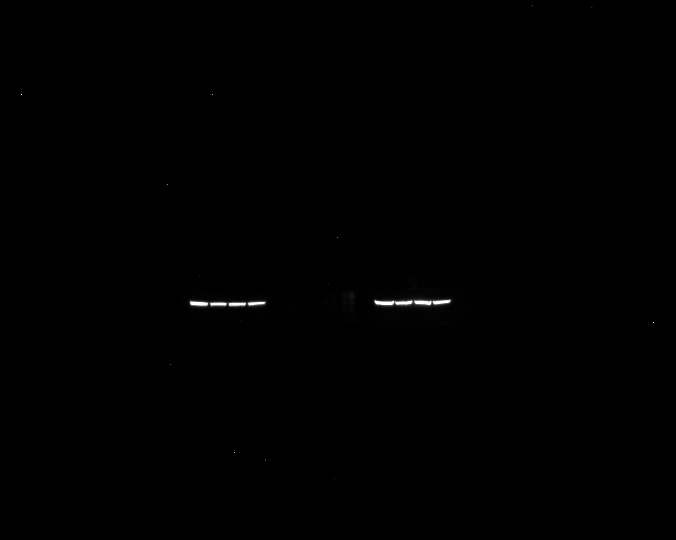

Supplement: Supplementary file 4 — Source data Fig. 1 [file 44318_2026_753_MOESM4_ESM.zip › Figure 1/1D/UFL1 cell lysate/CHEMI_10212025_181640Chemi.tif]

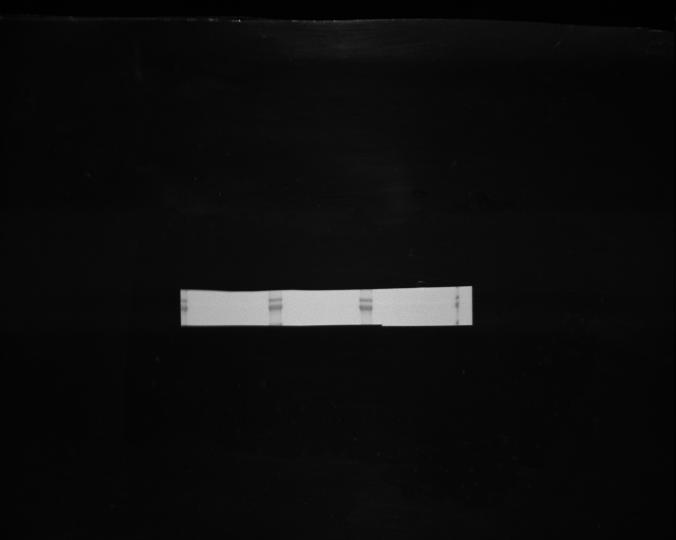

Supplement: Supplementary file 4 — Source data Fig. 1 [file 44318_2026_753_MOESM4_ESM.zip › Figure 1/1D/UFL1 cell lysate/CHEMI_10212025_181640Membrane.tif]

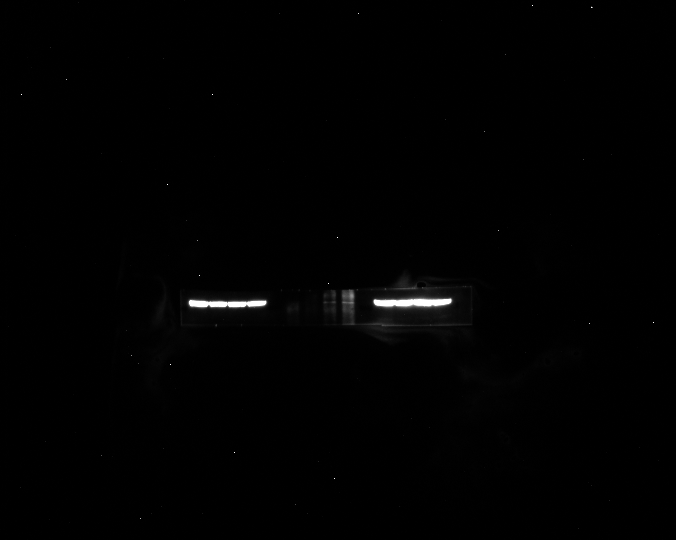

Supplement: Supplementary file 4 — Source data Fig. 1 [file 44318_2026_753_MOESM4_ESM.zip › Figure 1/1D/UFL1 IP FLAG/CHEMI_10212025_181828Chemi.tif]

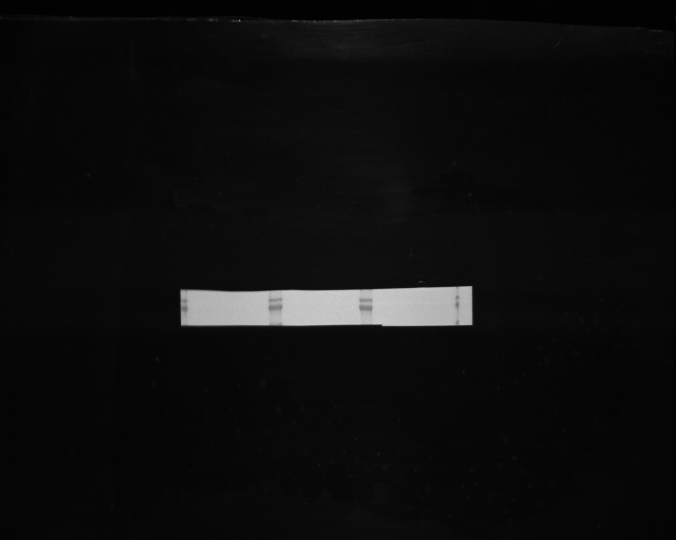

Supplement: Supplementary file 4 — Source data Fig. 1 [file 44318_2026_753_MOESM4_ESM.zip › Figure 1/1D/UFL1 IP FLAG/CHEMI_10212025_181828Membrane.tif]

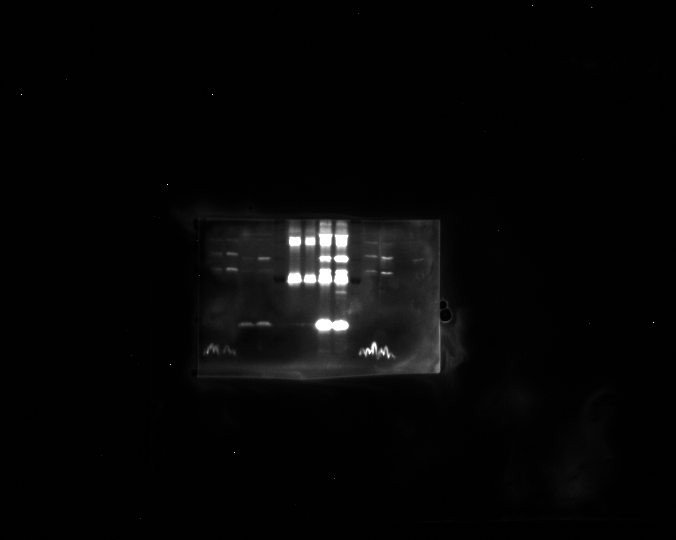

Supplement: Supplementary file 4 — Source data Fig. 1 [file 44318_2026_753_MOESM4_ESM.zip › Figure 1/1D/UFM1 cell lysate/CHEMI_10212025_185748Chemi.tif]

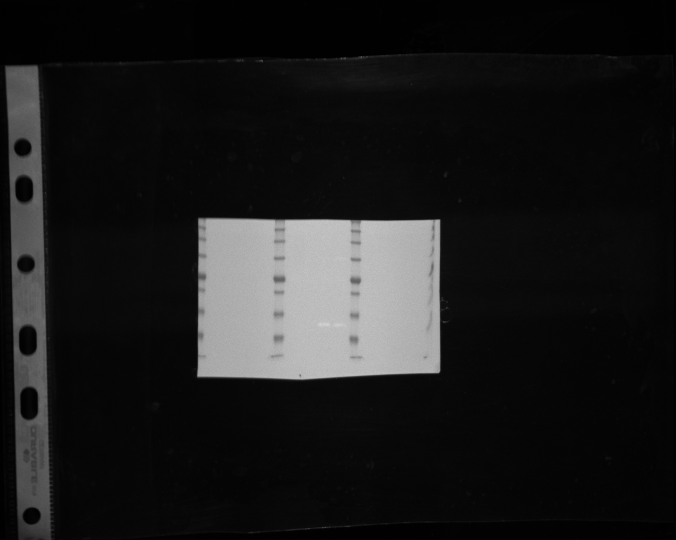

Supplement: Supplementary file 4 — Source data Fig. 1 [file 44318_2026_753_MOESM4_ESM.zip › Figure 1/1D/UFM1 cell lysate/CHEMI_10212025_185748Membrane.tif]

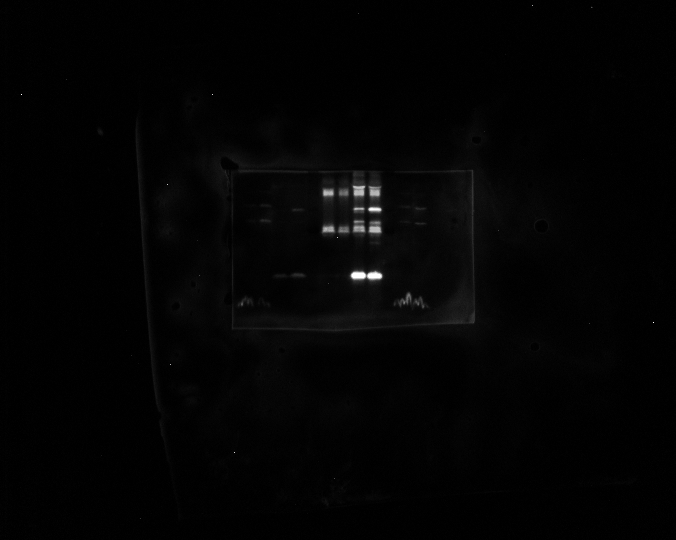

Supplement: Supplementary file 4 — Source data Fig. 1 [file 44318_2026_753_MOESM4_ESM.zip › Figure 1/1D/UFM1 IP FLAG/CHEMI_10212025_185136Chemi.tif]

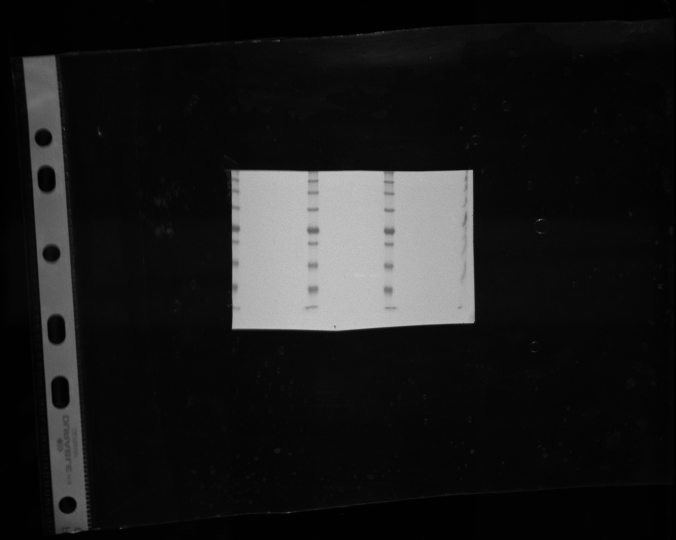

Supplement: Supplementary file 4 — Source data Fig. 1 [file 44318_2026_753_MOESM4_ESM.zip › Figure 1/1D/UFM1 IP FLAG/CHEMI_10212025_185136Membrane.tif]

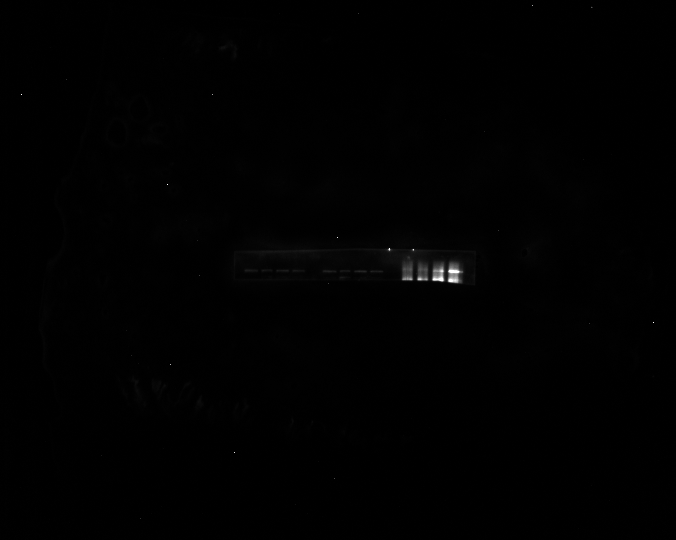

Supplement: Supplementary file 4 — Source data Fig. 1 [file 44318_2026_753_MOESM4_ESM.zip › Figure 1/1E/NEMF cell lysate/CHEMI_10072025_131136Chemi.tif]

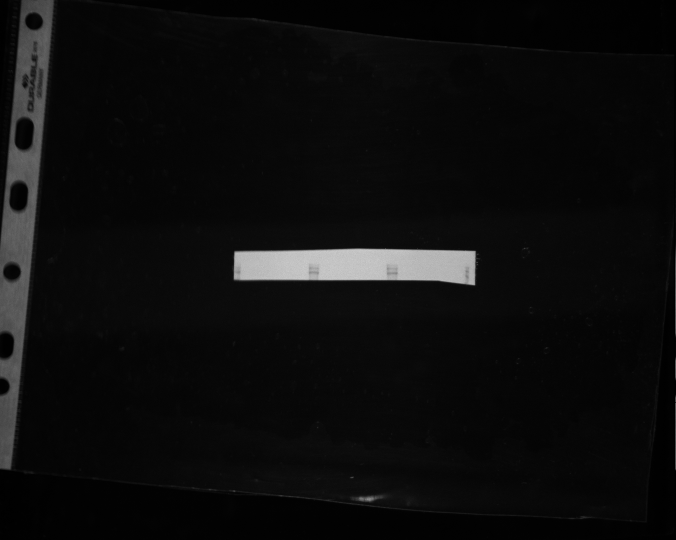

Supplement: Supplementary file 4 — Source data Fig. 1 [file 44318_2026_753_MOESM4_ESM.zip › Figure 1/1E/NEMF cell lysate/CHEMI_10072025_131136Membrane.tif]

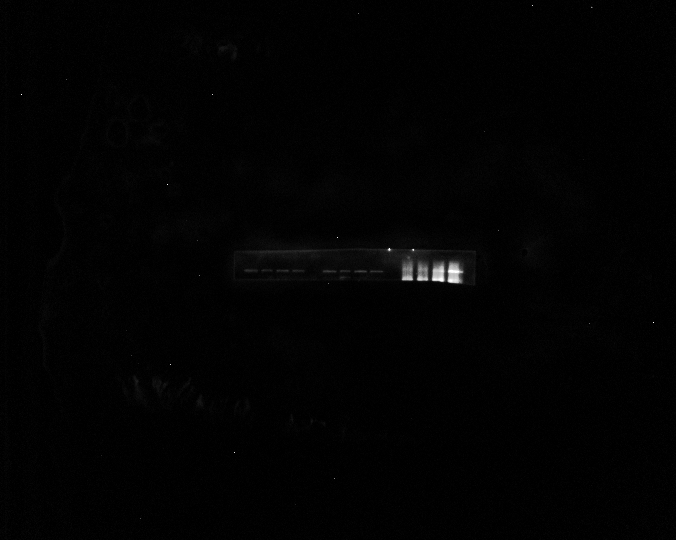

Supplement: Supplementary file 4 — Source data Fig. 1 [file 44318_2026_753_MOESM4_ESM.zip › Figure 1/1E/NEMF IP FLAG/CHEMI_10072025_131114Chemi.tif]

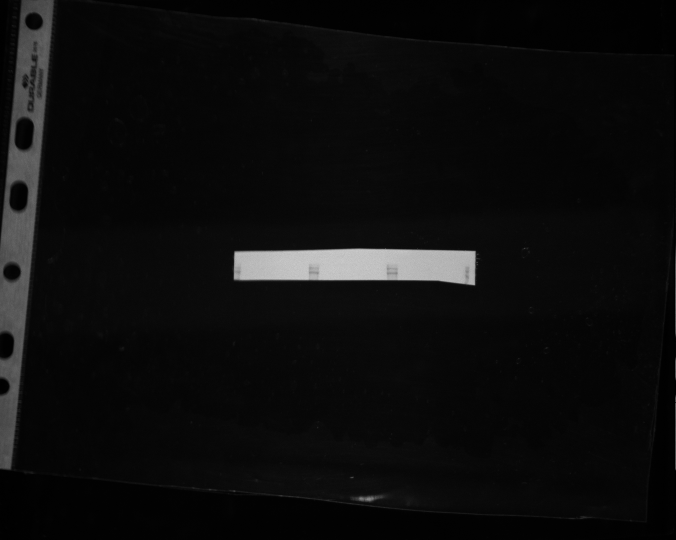

Supplement: Supplementary file 4 — Source data Fig. 1 [file 44318_2026_753_MOESM4_ESM.zip › Figure 1/1E/NEMF IP FLAG/CHEMI_10072025_131114Membrane.tif]

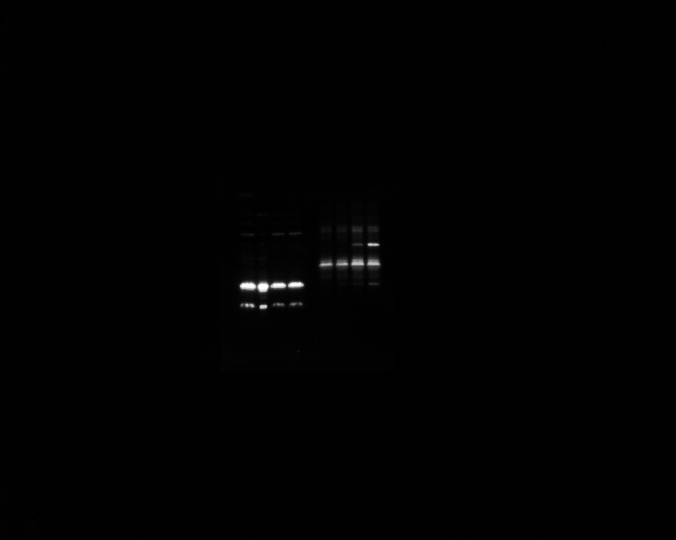

Supplement: Supplementary file 4 — Source data Fig. 1 [file 44318_2026_753_MOESM4_ESM.zip › Figure 1/1E/RPL26 cell lysate/CHEMI_11122025_133113Chemi.tif]

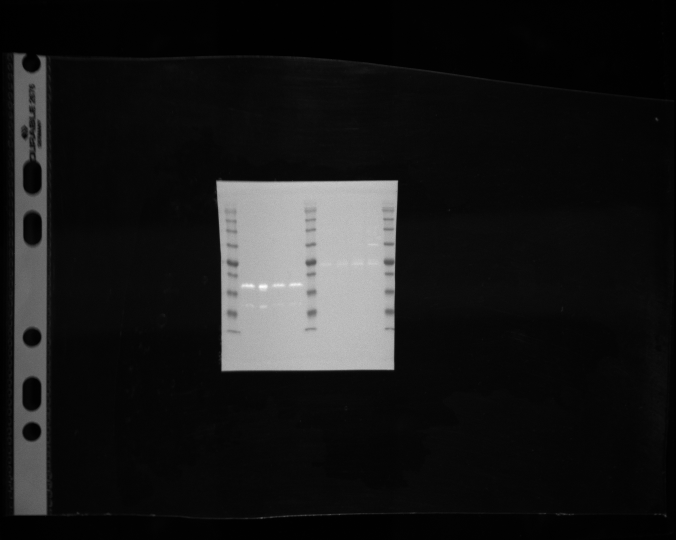

Supplement: Supplementary file 4 — Source data Fig. 1 [file 44318_2026_753_MOESM4_ESM.zip › Figure 1/1E/RPL26 cell lysate/CHEMI_11122025_133113Membrane.tif]

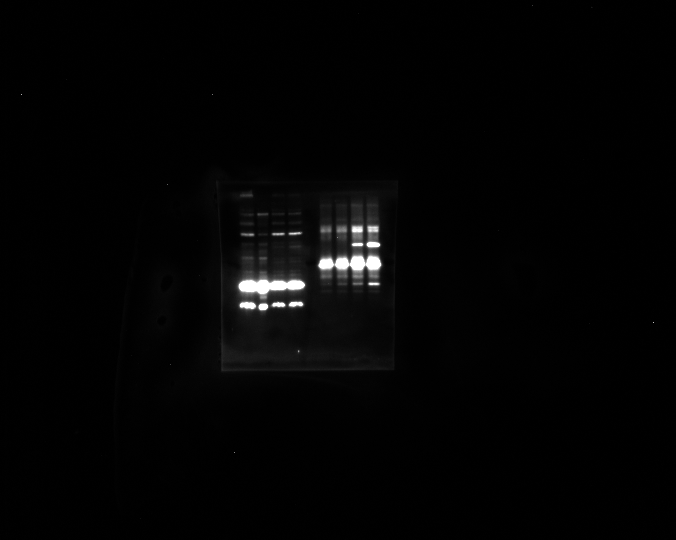

Supplement: Supplementary file 4 — Source data Fig. 1 [file 44318_2026_753_MOESM4_ESM.zip › Figure 1/1E/RPL26 IP FLAG/CHEMI_11122025_133135Chemi.tif]

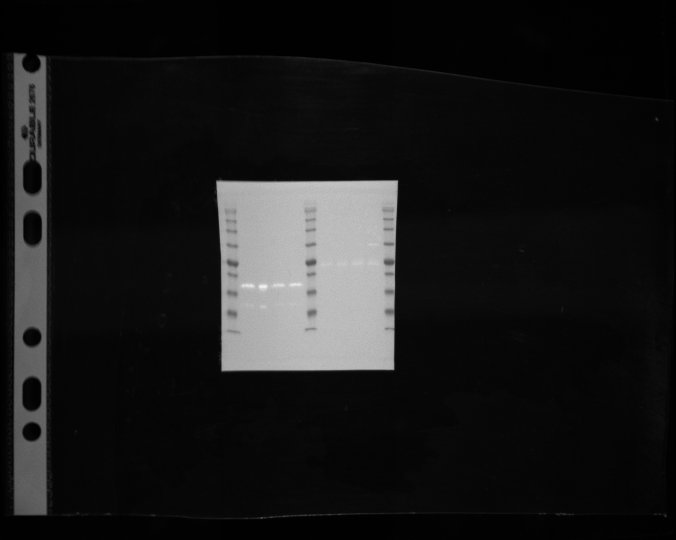

Supplement: Supplementary file 4 — Source data Fig. 1 [file 44318_2026_753_MOESM4_ESM.zip › Figure 1/1E/RPL26 IP FLAG/CHEMI_11122025_133135Membrane.tif]

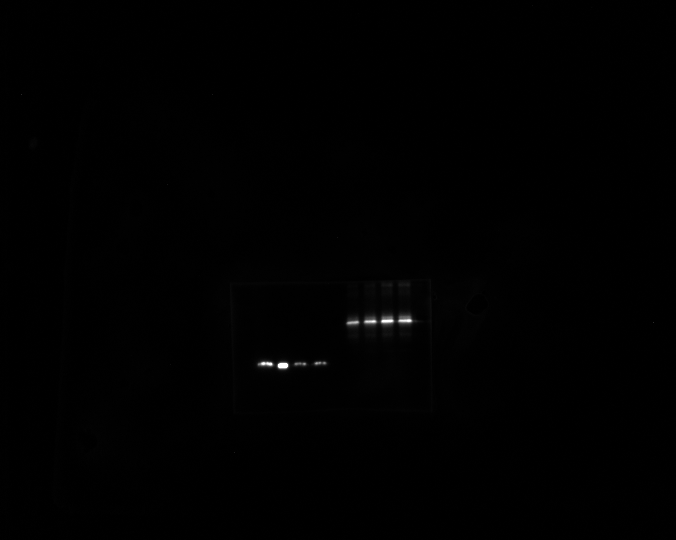

Supplement: Supplementary file 4 — Source data Fig. 1 [file 44318_2026_753_MOESM4_ESM.zip › Figure 1/1E/RPS20/CHEMI_11122025_133603Chemi.tif]

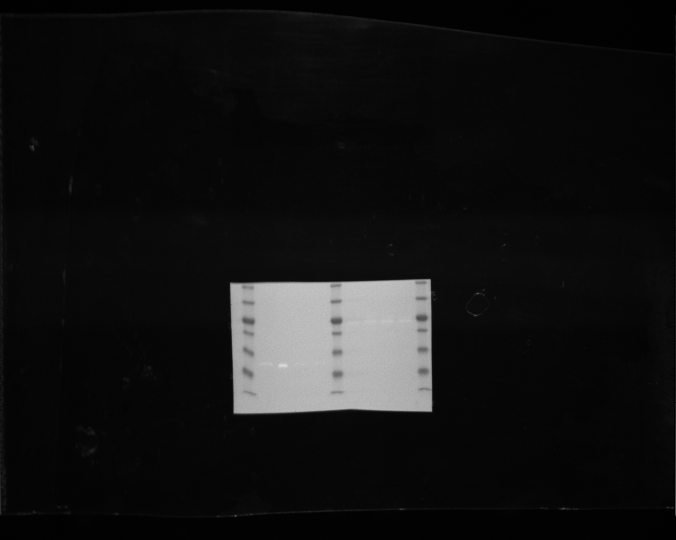

Supplement: Supplementary file 4 — Source data Fig. 1 [file 44318_2026_753_MOESM4_ESM.zip › Figure 1/1E/RPS20/CHEMI_11122025_133603Membrane.tif]

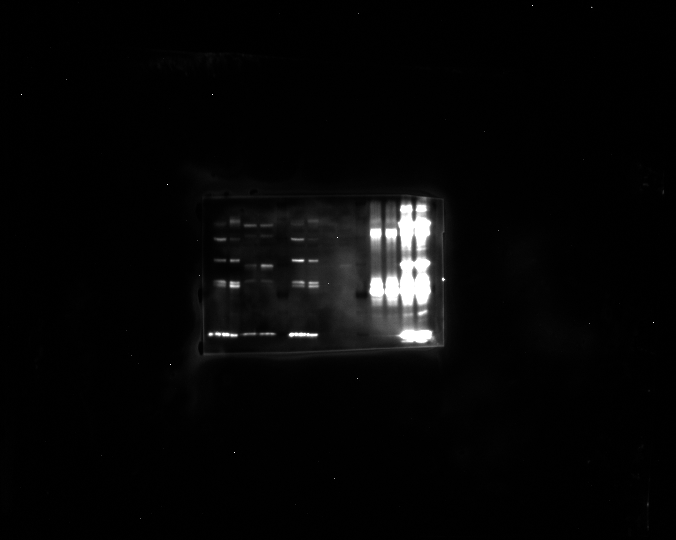

Supplement: Supplementary file 4 — Source data Fig. 1 [file 44318_2026_753_MOESM4_ESM.zip › Figure 1/1E/UFM1 cell lysate/CHEMI_10072025_131824Chemi.tif]

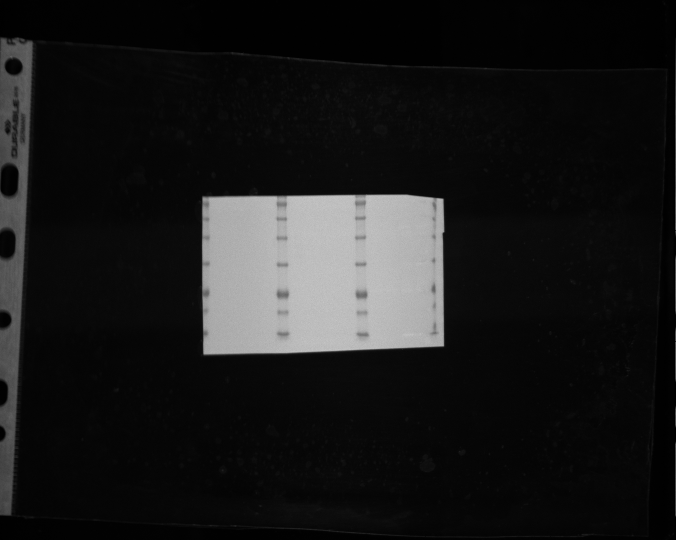

Supplement: Supplementary file 4 — Source data Fig. 1 [file 44318_2026_753_MOESM4_ESM.zip › Figure 1/1E/UFM1 cell lysate/CHEMI_10072025_131824Membrane.tif]

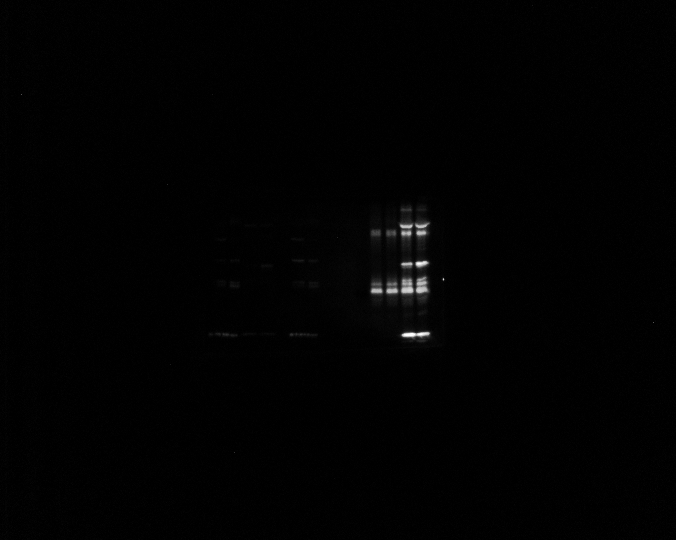

Supplement: Supplementary file 4 — Source data Fig. 1 [file 44318_2026_753_MOESM4_ESM.zip › Figure 1/1E/UFM1 IP FLAG/CHEMI_10072025_131615Chemi.tif]

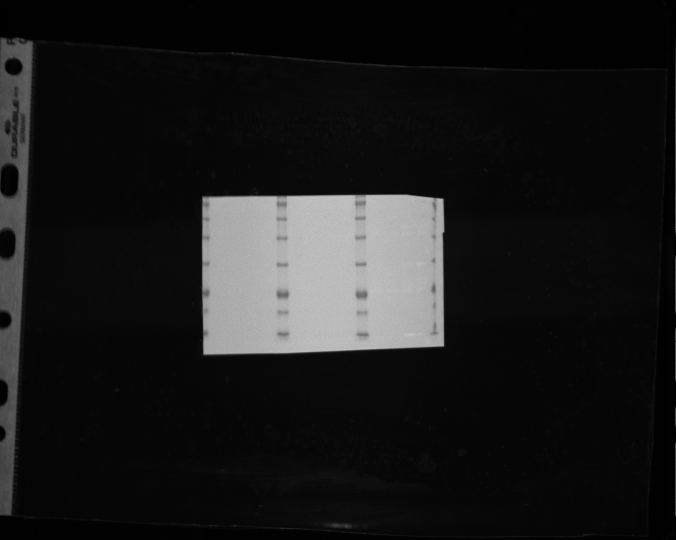

Supplement: Supplementary file 4 — Source data Fig. 1 [file 44318_2026_753_MOESM4_ESM.zip › Figure 1/1E/UFM1 IP FLAG/CHEMI_10072025_131615Membrane.tif]

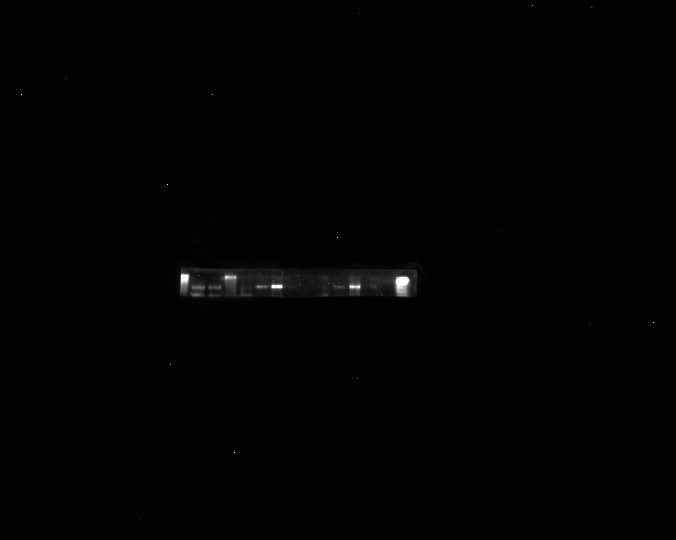

Supplement: Supplementary file 4 — Source data Fig. 1 [file 44318_2026_753_MOESM4_ESM.zip › Figure 1/1G/LTN1/CHEMI_09262025_124547Chemi.tif]

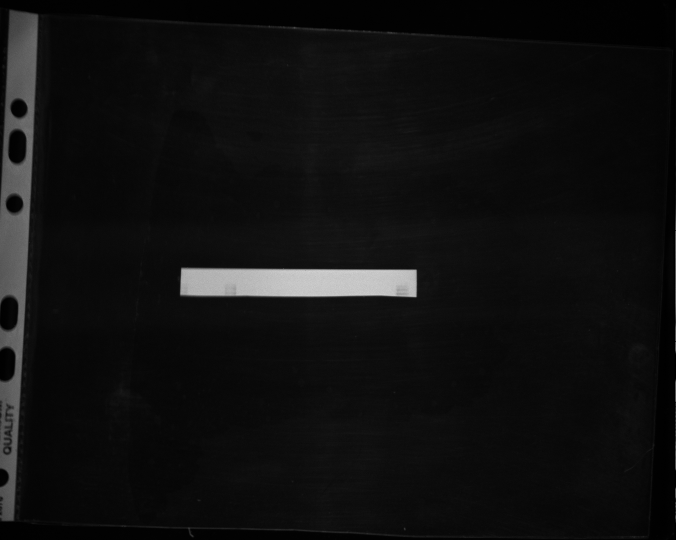

Supplement: Supplementary file 4 — Source data Fig. 1 [file 44318_2026_753_MOESM4_ESM.zip › Figure 1/1G/LTN1/CHEMI_09262025_124547Membrane.tif]

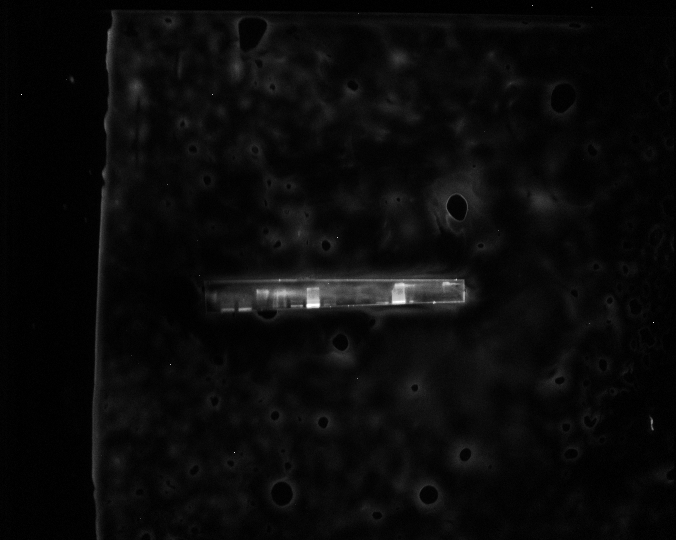

Supplement: Supplementary file 4 — Source data Fig. 1 [file 44318_2026_753_MOESM4_ESM.zip › Figure 1/1G/NEMF/CHEMI_09242025_184728Chemi.tif]

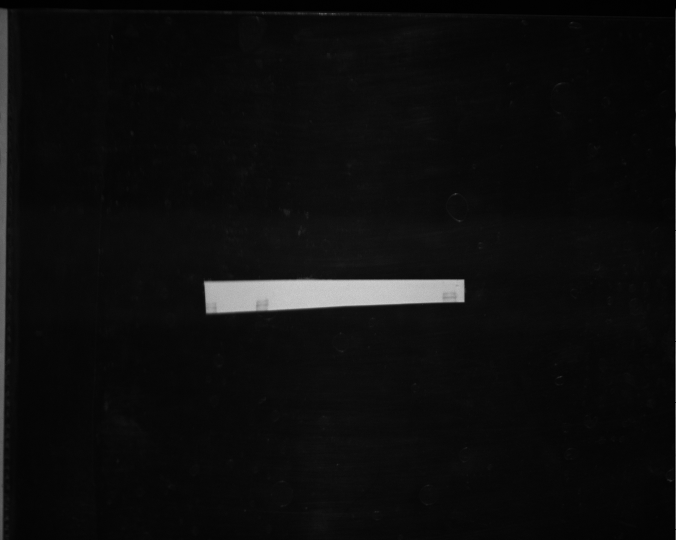

Supplement: Supplementary file 4 — Source data Fig. 1 [file 44318_2026_753_MOESM4_ESM.zip › Figure 1/1G/NEMF/CHEMI_09242025_184728Membrane.tif]

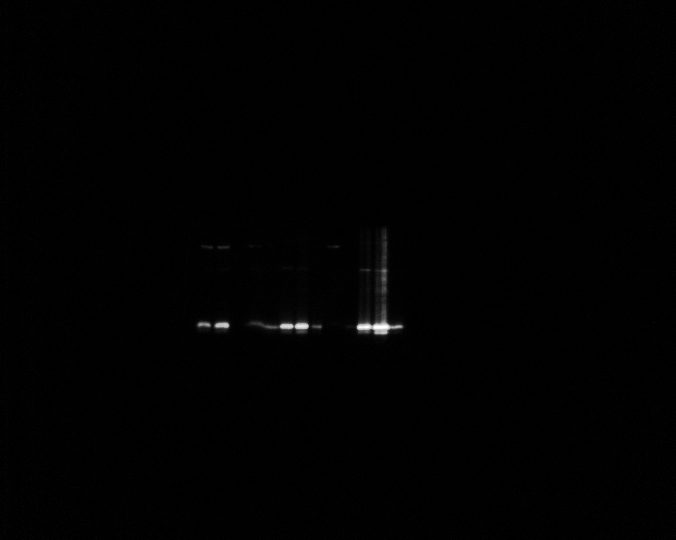

Supplement: Supplementary file 4 — Source data Fig. 1 [file 44318_2026_753_MOESM4_ESM.zip › Figure 1/1G/RPL26/CHEMI_09262025_142947Chemi.tif]

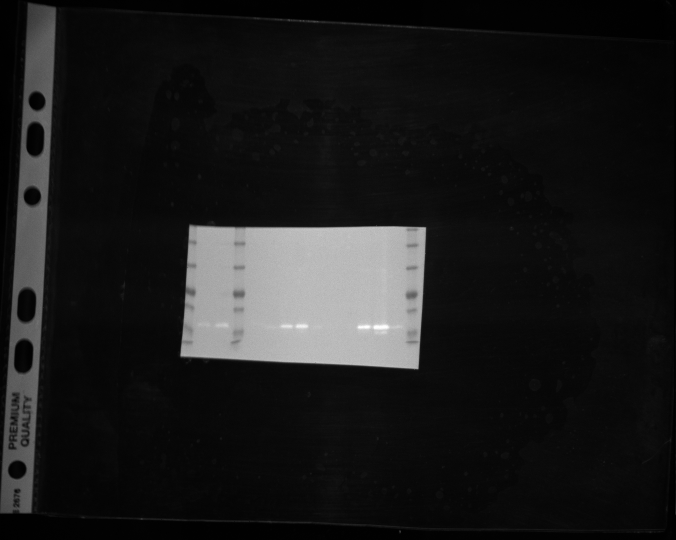

Supplement: Supplementary file 4 — Source data Fig. 1 [file 44318_2026_753_MOESM4_ESM.zip › Figure 1/1G/RPL26/CHEMI_09262025_142947Membrane.tif]

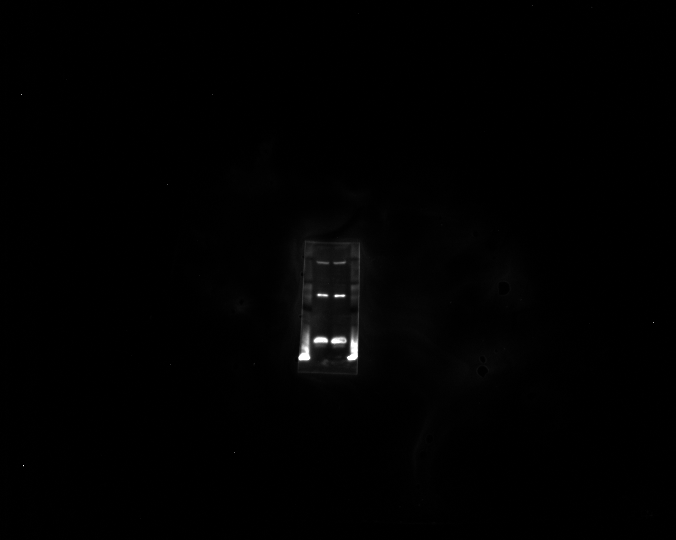

Supplement: Supplementary file 4 — Source data Fig. 1 [file 44318_2026_753_MOESM4_ESM.zip › Figure 1/1G/RPS3 cell lysate/CHEMI_09292025_125858Chemi.tif]

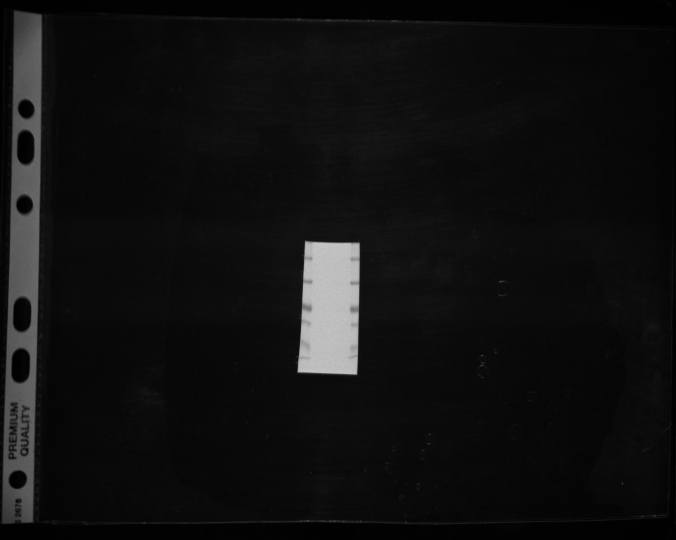

Supplement: Supplementary file 4 — Source data Fig. 1 [file 44318_2026_753_MOESM4_ESM.zip › Figure 1/1G/RPS3 cell lysate/CHEMI_09292025_125858Membrane.tif]

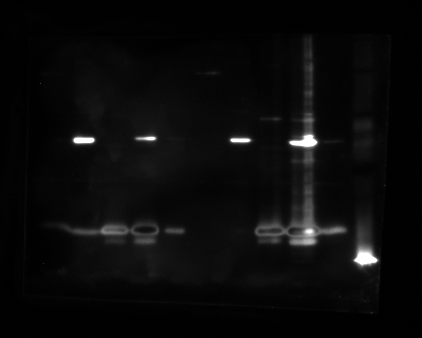

Supplement: Supplementary file 4 — Source data Fig. 1 [file 44318_2026_753_MOESM4_ESM.zip › Figure 1/1G/RPS3 sucrose fractions/CHEMI_09292025_125654Chemi.tif]

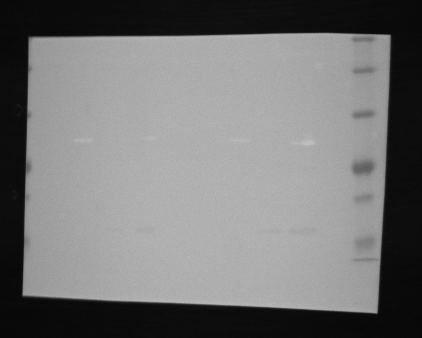

Supplement: Supplementary file 4 — Source data Fig. 1 [file 44318_2026_753_MOESM4_ESM.zip › Figure 1/1G/RPS3 sucrose fractions/CHEMI_09292025_125654Membrane.tif]

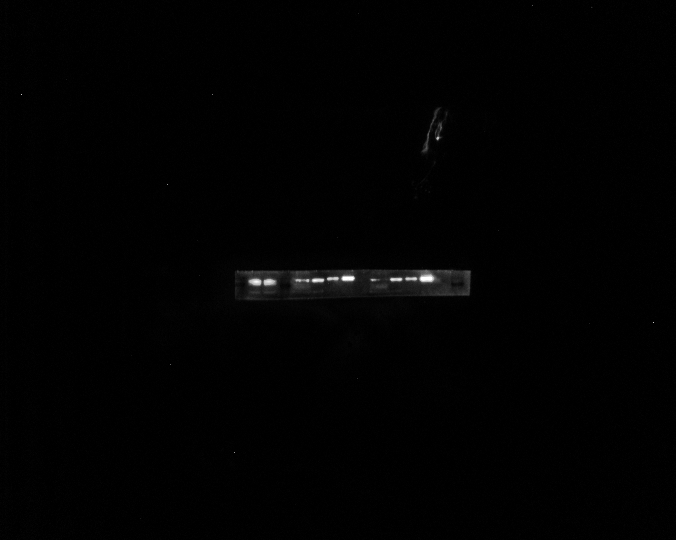

Supplement: Supplementary file 4 — Source data Fig. 1 [file 44318_2026_753_MOESM4_ESM.zip › Figure 1/1G/UFL1/CHEMI_09232025_125616Chemi.tif]

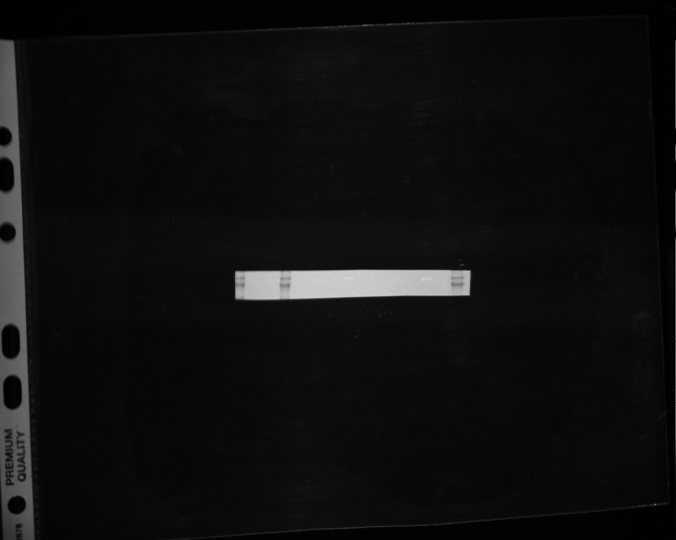

Supplement: Supplementary file 4 — Source data Fig. 1 [file 44318_2026_753_MOESM4_ESM.zip › Figure 1/1G/UFL1/CHEMI_09232025_125616Membrane.tif]

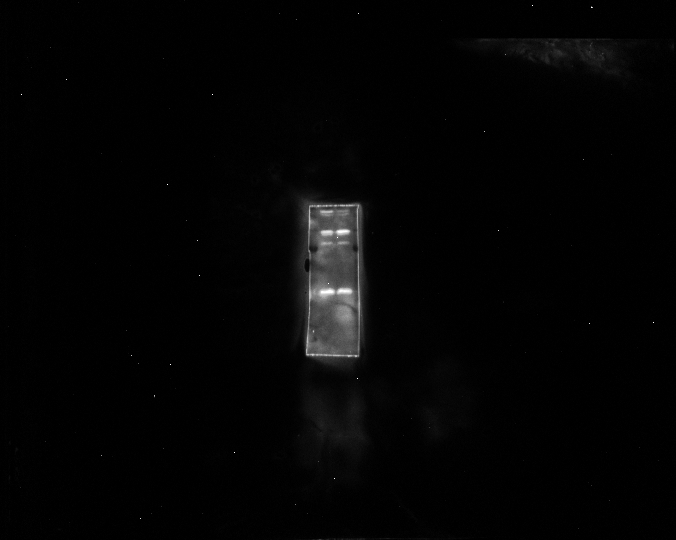

Supplement: Supplementary file 4 — Source data Fig. 1 [file 44318_2026_753_MOESM4_ESM.zip › Figure 1/1G/UFM1 cell lysate/CHEMI_09262025_145219Chemi.tif]

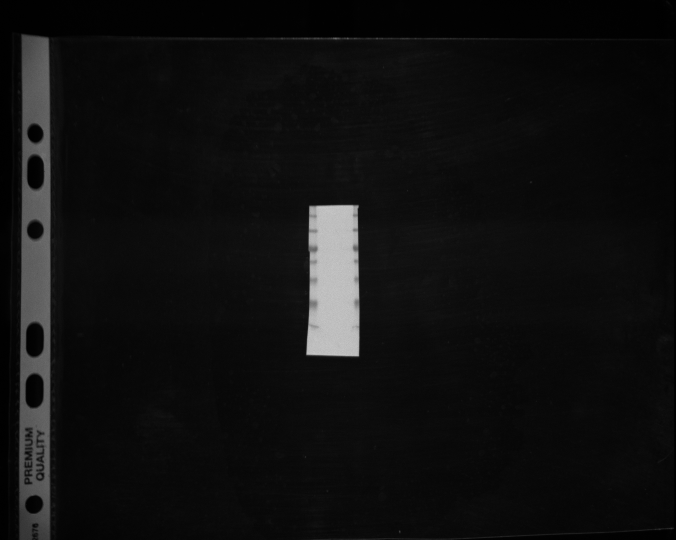

Supplement: Supplementary file 4 — Source data Fig. 1 [file 44318_2026_753_MOESM4_ESM.zip › Figure 1/1G/UFM1 cell lysate/CHEMI_09262025_145219Membrane.tif]

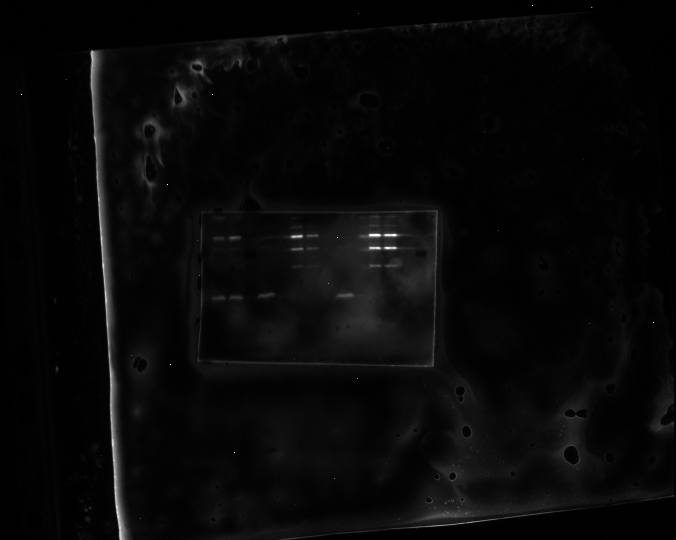

Supplement: Supplementary file 4 — Source data Fig. 1 [file 44318_2026_753_MOESM4_ESM.zip › Figure 1/1G/UFM1 sucrose fractions/CHEMI_09262025_144447Chemi.tif]

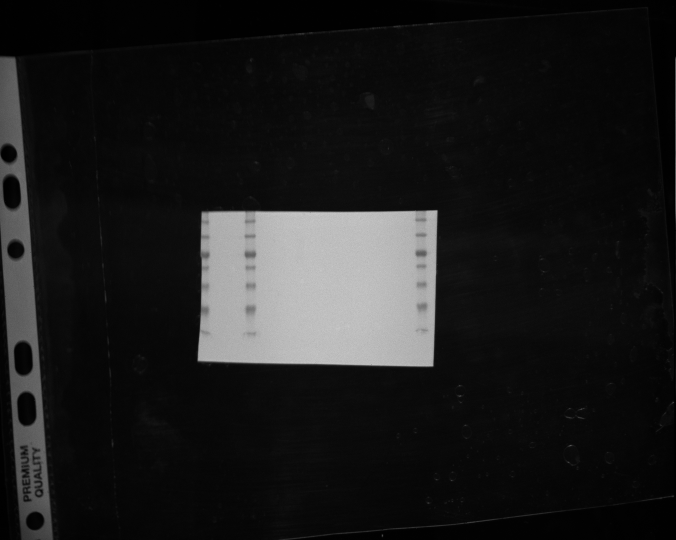

Supplement: Supplementary file 4 — Source data Fig. 1 [file 44318_2026_753_MOESM4_ESM.zip › Figure 1/1G/UFM1 sucrose fractions/CHEMI_09262025_144447Membrane.tif]

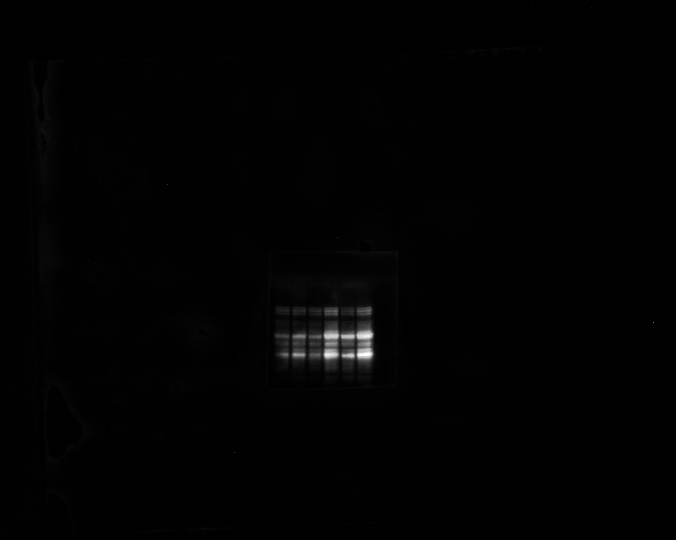

Supplement: Supplementary file 5 — Source data Fig. 2 [file 44318_2026_753_MOESM5_ESM.zip › Figure 2/2D/FLAG/CHEMI_11222024_130930Chemi.tif]

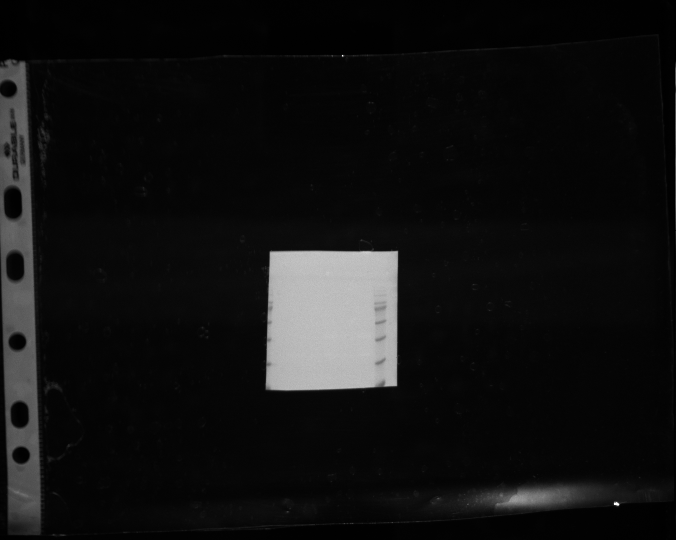

Supplement: Supplementary file 5 — Source data Fig. 2 [file 44318_2026_753_MOESM5_ESM.zip › Figure 2/2D/FLAG/CHEMI_11222024_130930Membrane.tif]

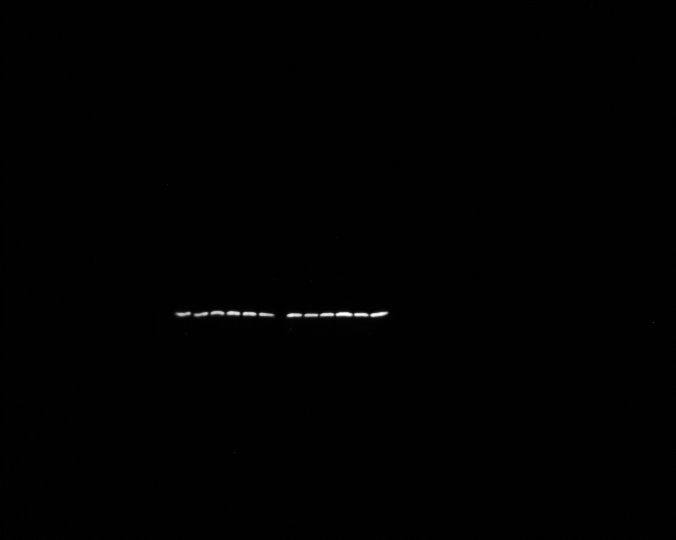

Supplement: Supplementary file 5 — Source data Fig. 2 [file 44318_2026_753_MOESM5_ESM.zip › Figure 2/2D/RPS10/CHEMI_11222024_131724Chemi.tif]

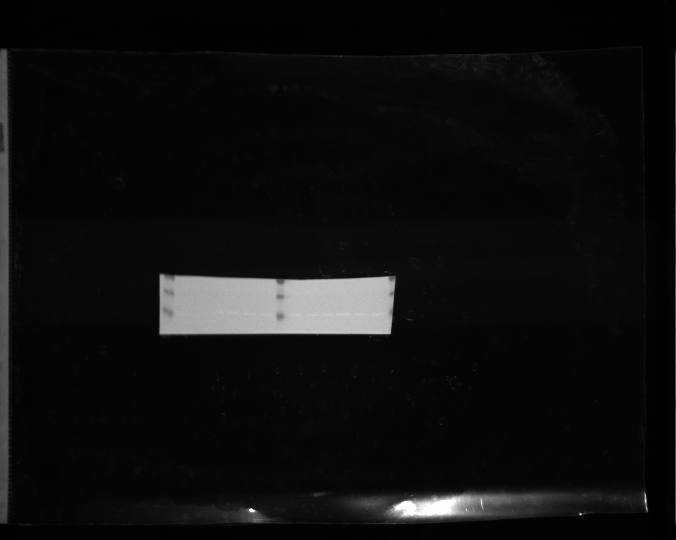

Supplement: Supplementary file 5 — Source data Fig. 2 [file 44318_2026_753_MOESM5_ESM.zip › Figure 2/2D/RPS10/CHEMI_11222024_131724Membrane.tif]

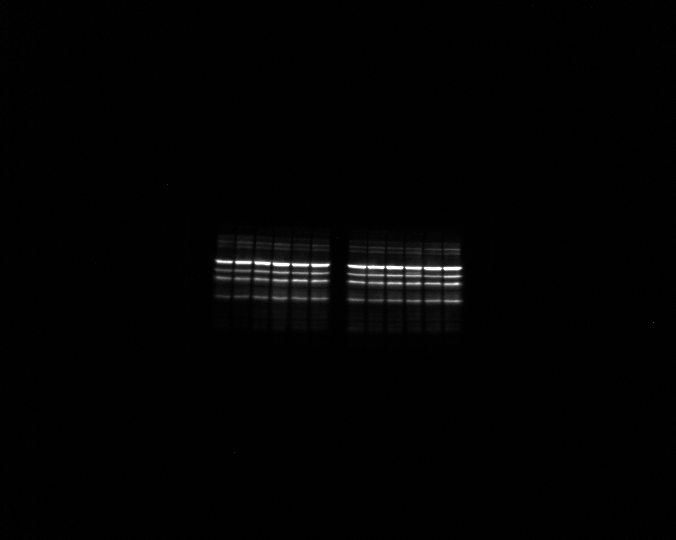

Supplement: Supplementary file 5 — Source data Fig. 2 [file 44318_2026_753_MOESM5_ESM.zip › Figure 2/2F/FLAG/CHEMI_11222024_130157Chemi.tif]

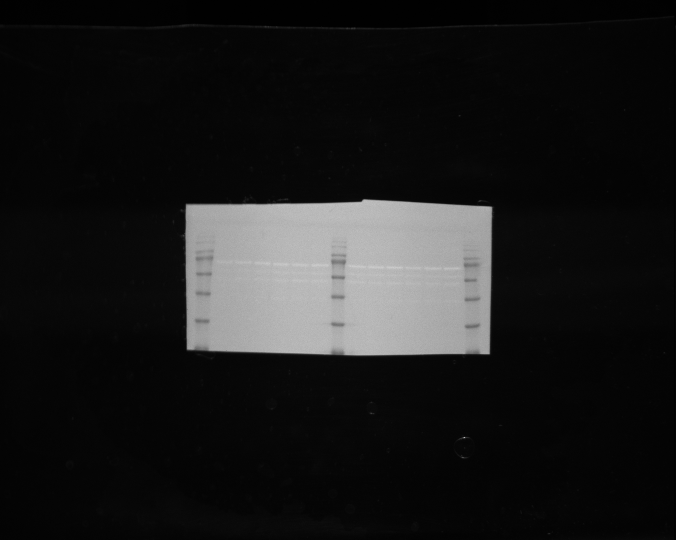

Supplement: Supplementary file 5 — Source data Fig. 2 [file 44318_2026_753_MOESM5_ESM.zip › Figure 2/2F/FLAG/CHEMI_11222024_130157Membrane.tif]

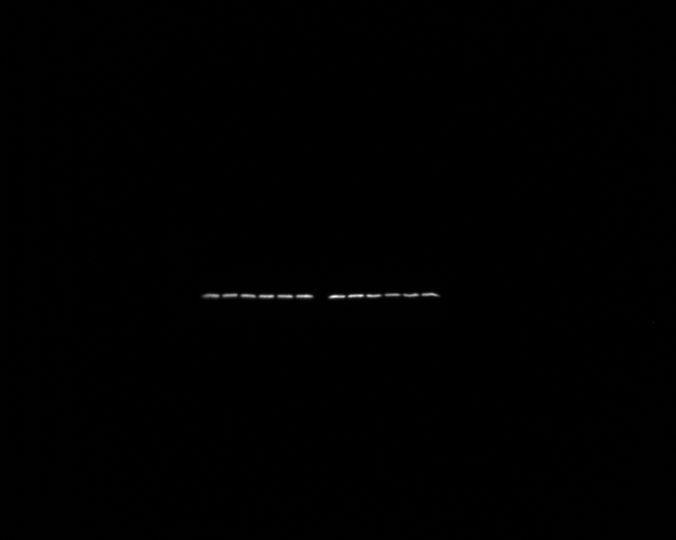

Supplement: Supplementary file 5 — Source data Fig. 2 [file 44318_2026_753_MOESM5_ESM.zip › Figure 2/2F/RPS10/CHEMI_11222024_131223Chemi.tif]

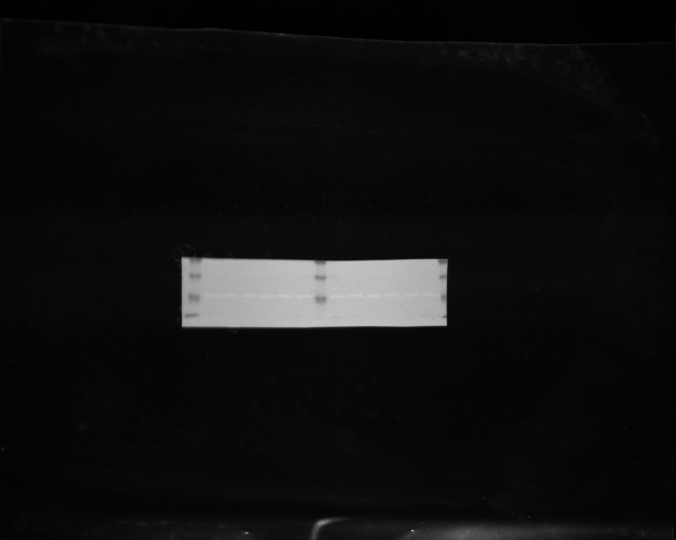

Supplement: Supplementary file 5 — Source data Fig. 2 [file 44318_2026_753_MOESM5_ESM.zip › Figure 2/2F/RPS10/CHEMI_11222024_131223Membrane.tif]

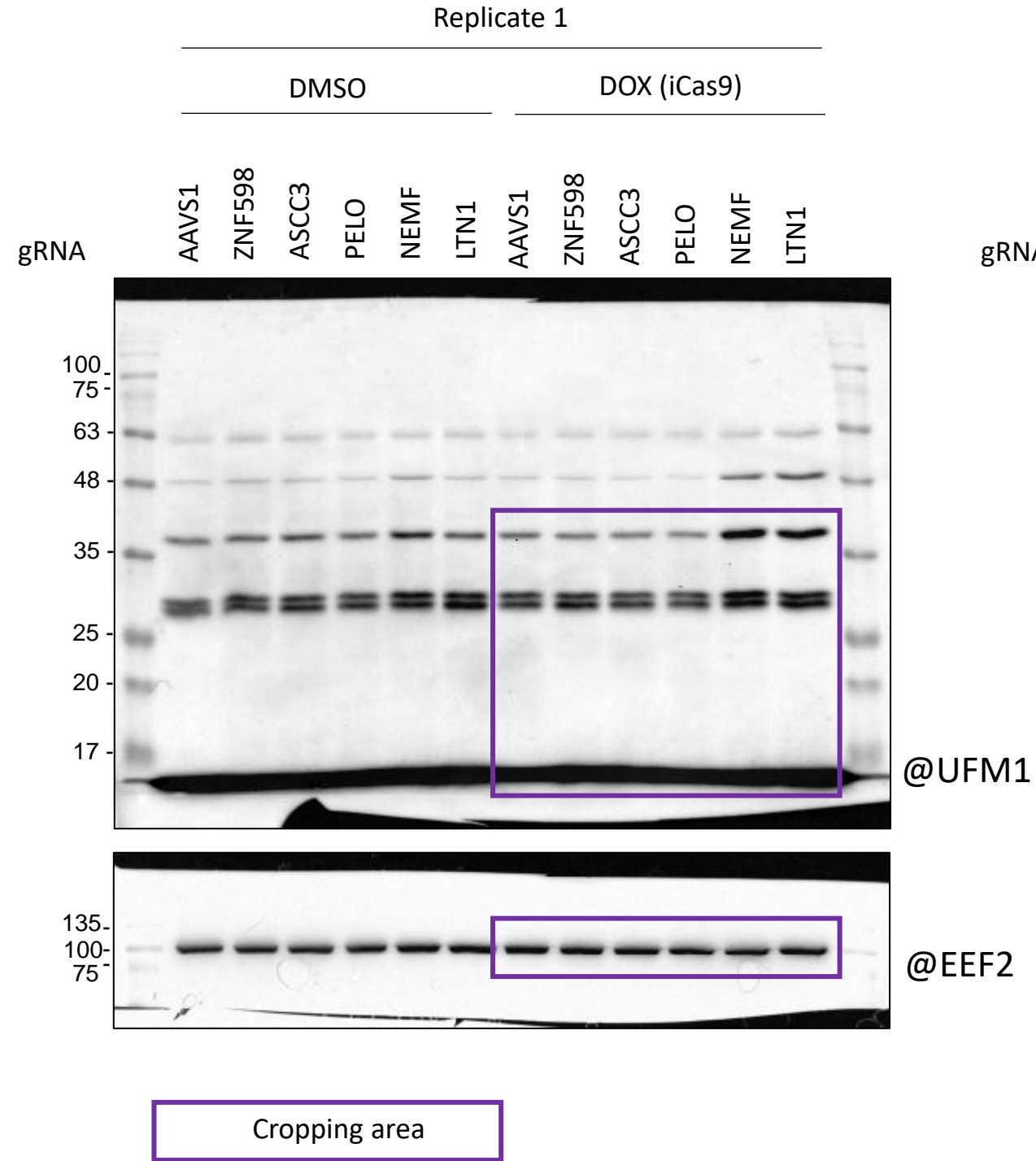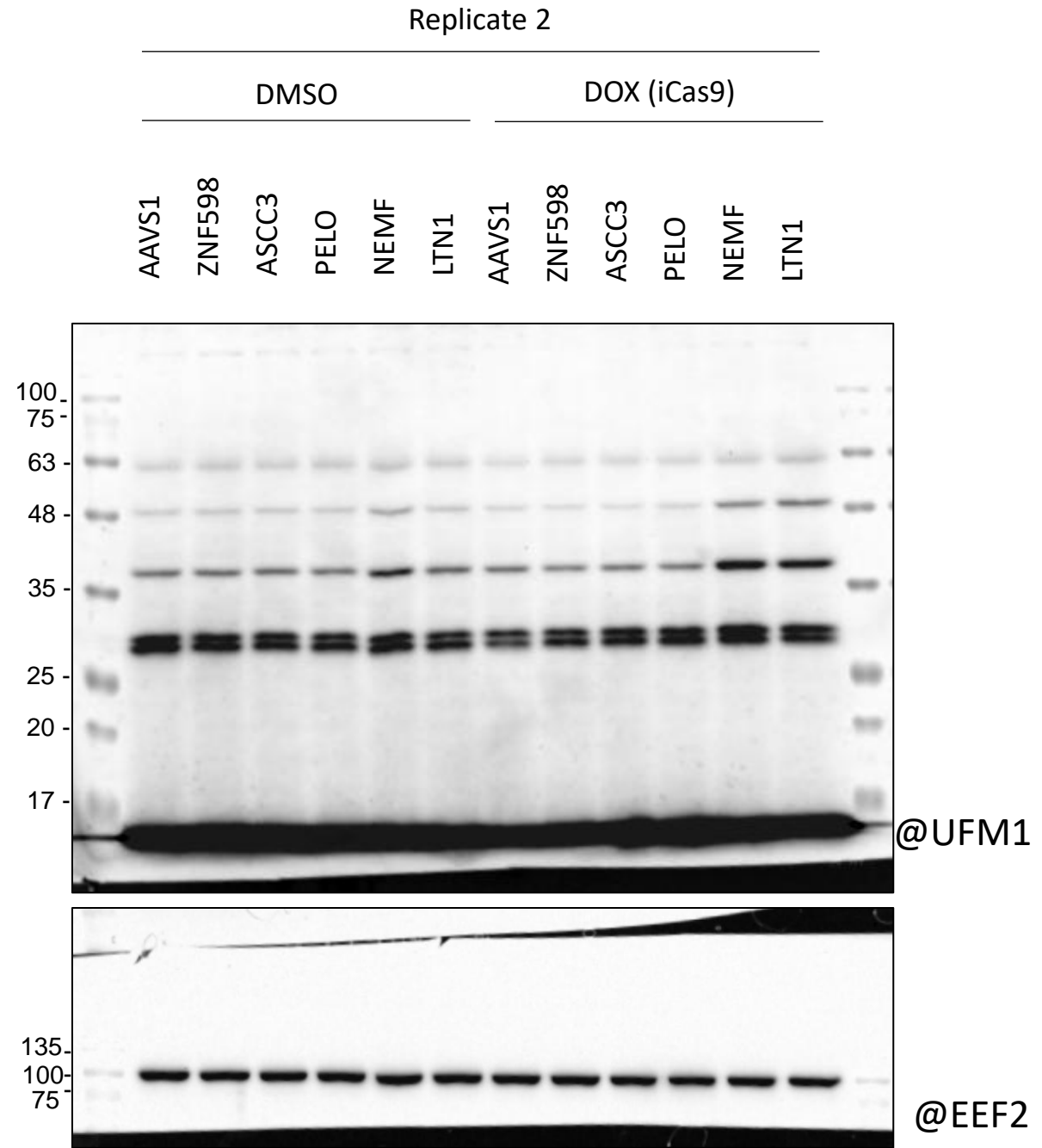

Replicate 3

DMSO

DOX (iCas9)

gRNA

AAVS1  
ZNF598  
ASCC3  
PELO  
NEMF  
LTN1  
AAVS1  
ZNF598  
ASCC3  
PELO  
NEMF  
LTN1

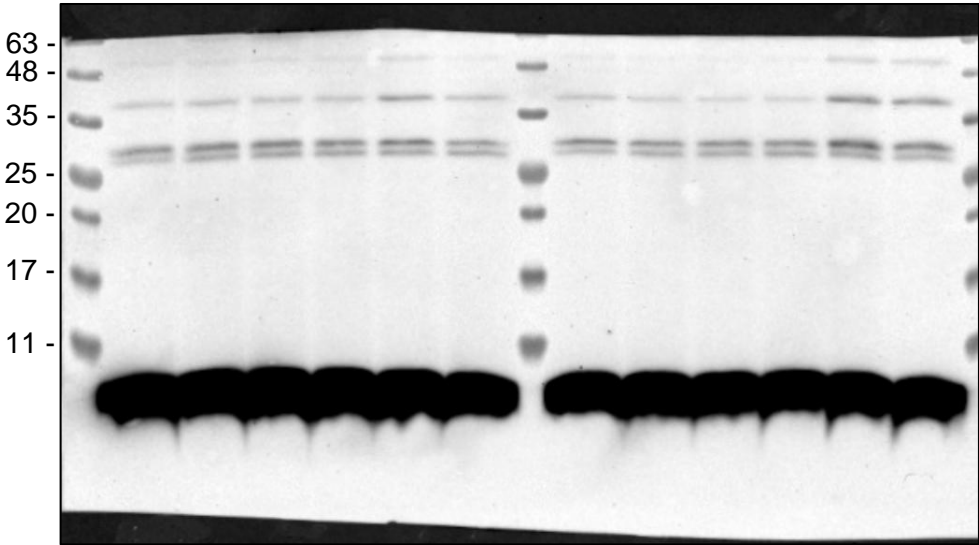

@UFM1

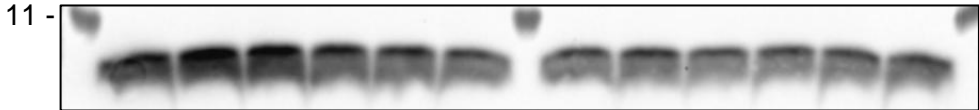

@UFM1

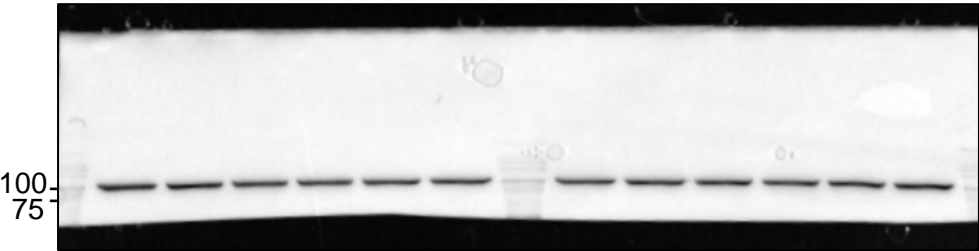

@EEF2

Supplement: Supplementary file 6 — Source data Fig. 3 [file 44318_2026_753_MOESM6_ESM.zip › Figure 3/3A/Fig3A Western Blot labeled cropping.pdf]

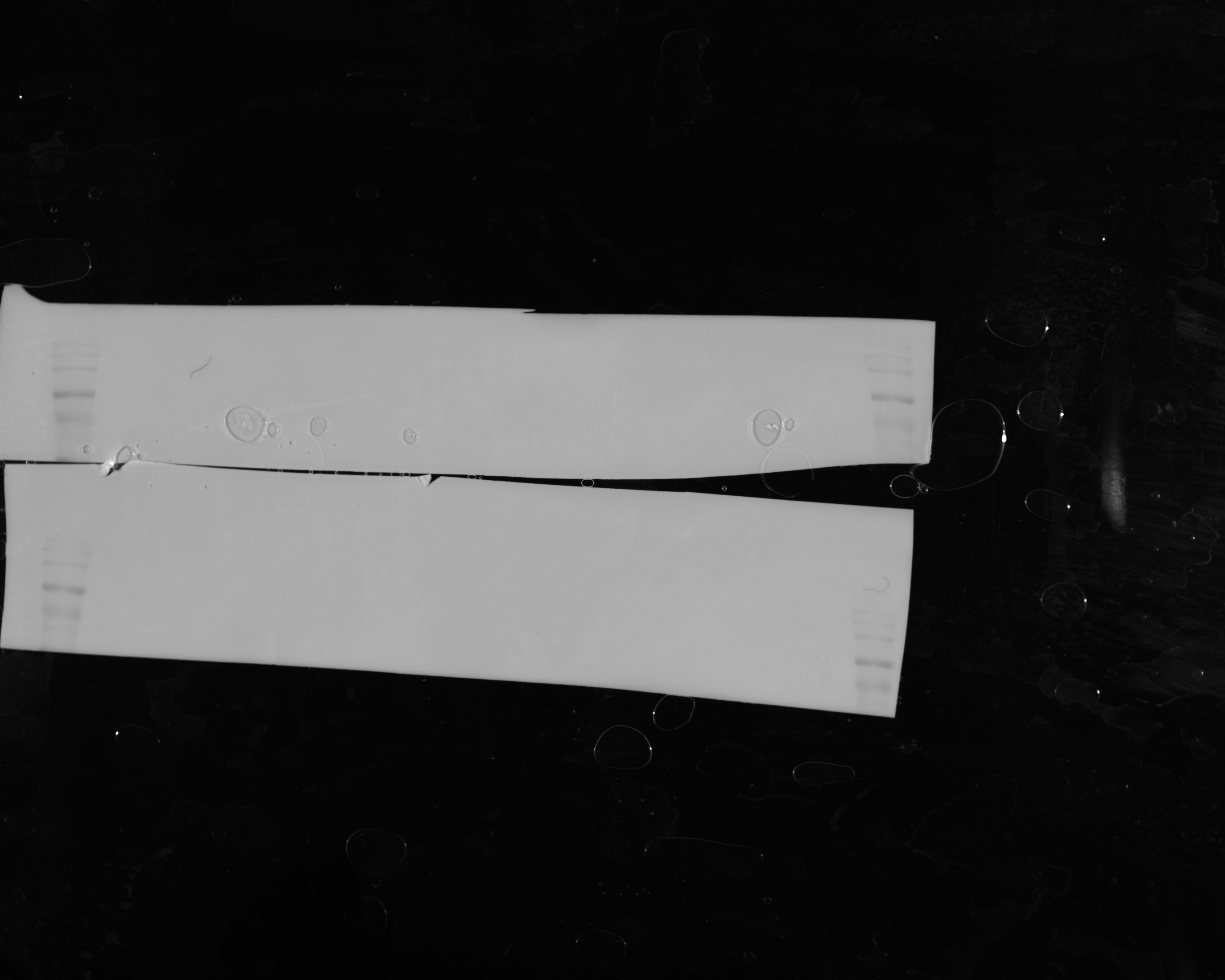

Supplement: Supplementary file 6 — Source data Fig. 3 [file 44318_2026_753_MOESM6_ESM.zip › Figure 3/3A/Membrane EEF2 replicates 1 upper and 2 lower.tif]

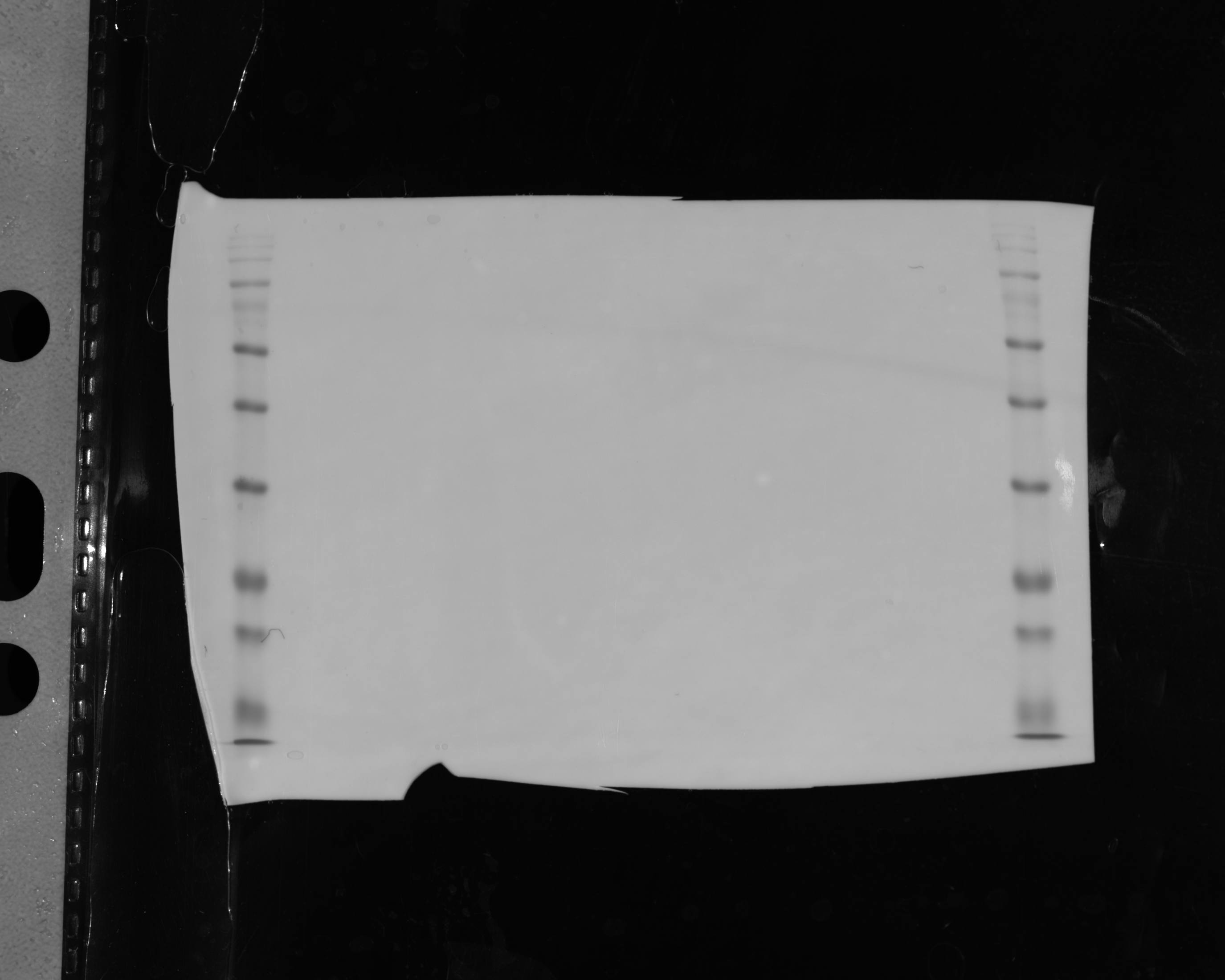

Supplement: Supplementary file 6 — Source data Fig. 3 [file 44318_2026_753_MOESM6_ESM.zip › Figure 3/3A/Membrane UFM1.tif]

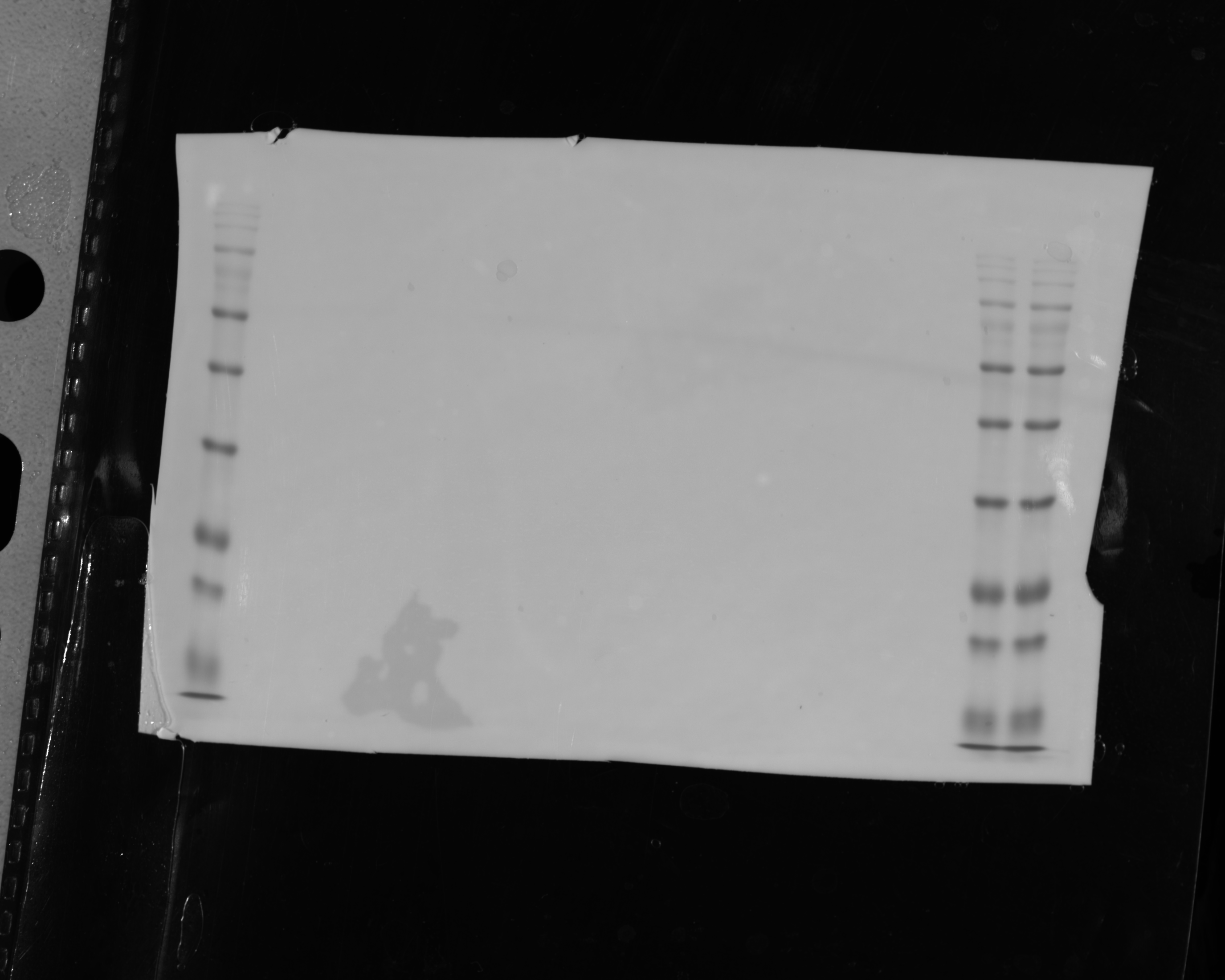

Supplement: Supplementary file 6 — Source data Fig. 3 [file 44318_2026_753_MOESM6_ESM.zip › Figure 3/3A/replicate 2/Membrane UFM1 replicate 2.tif]

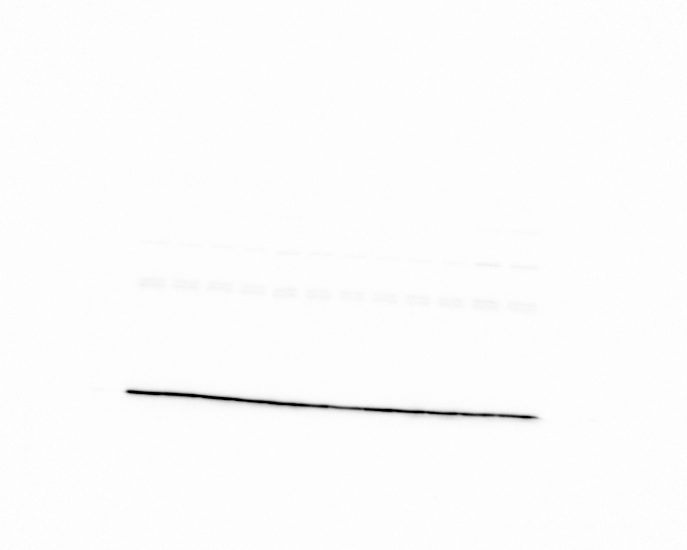

Supplement: Supplementary file 6 — Source data Fig. 3 [file 44318_2026_753_MOESM6_ESM.zip › Figure 3/3A/replicate 2/WB UFM1 lower exposure replicate 2.tif]

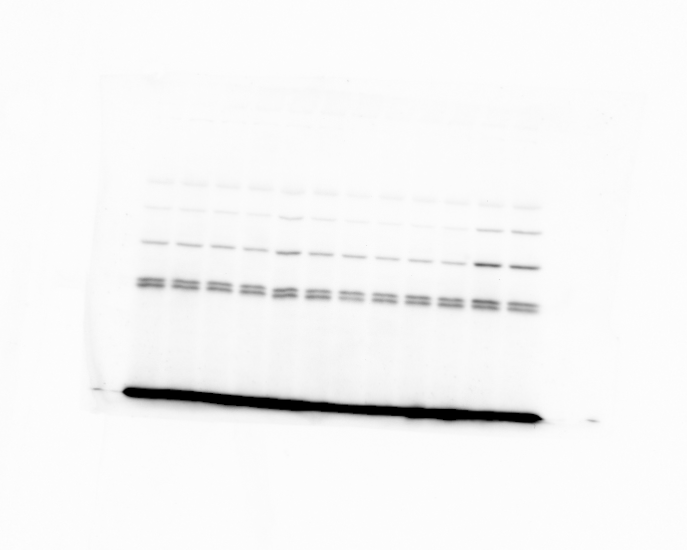

Supplement: Supplementary file 6 — Source data Fig. 3 [file 44318_2026_753_MOESM6_ESM.zip › Figure 3/3A/replicate 2/WB UFM1 replicate 2.tif]

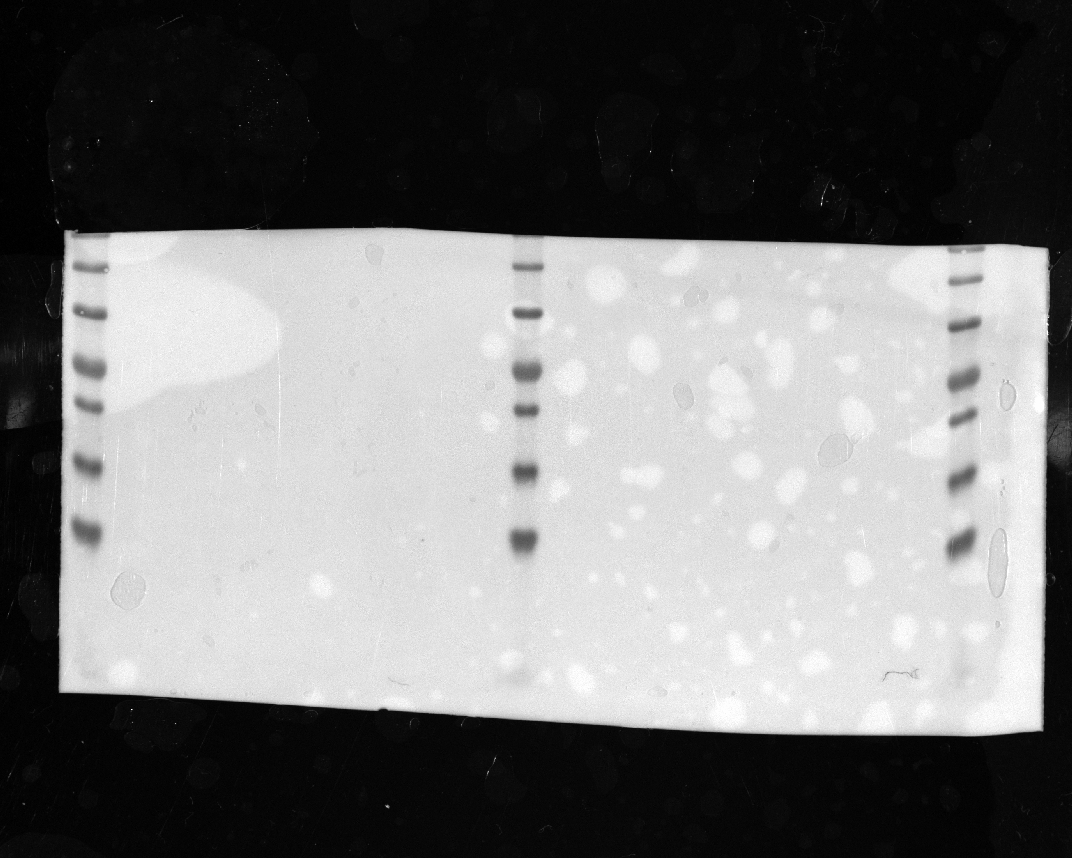

Supplement: Supplementary file 6 — Source data Fig. 3 [file 44318_2026_753_MOESM6_ESM.zip › Figure 3/3A/replicate 3/Membrane UFM1 replicate 3.tif]

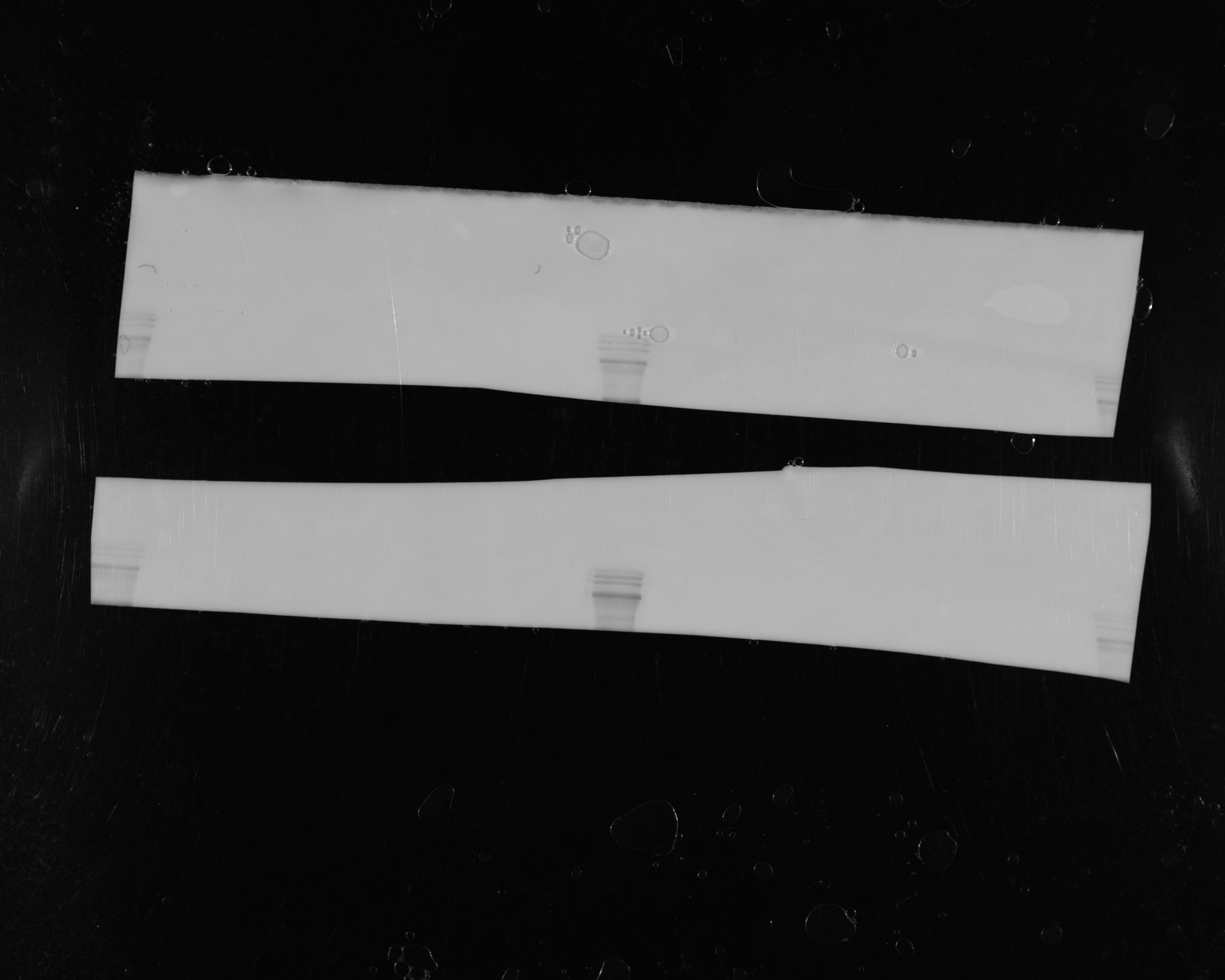

Supplement: Supplementary file 6 — Source data Fig. 3 [file 44318_2026_753_MOESM6_ESM.zip › Figure 3/3A/replicate 3/Membrane WB EEF2 replicate 3 upper.tif]

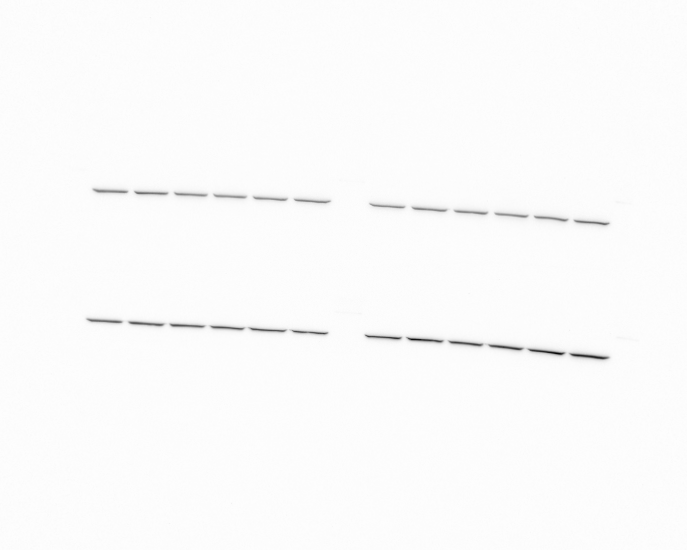

Supplement: Supplementary file 6 — Source data Fig. 3 [file 44318_2026_753_MOESM6_ESM.zip › Figure 3/3A/replicate 3/WB EEF2 replicate 3 upper.tif]

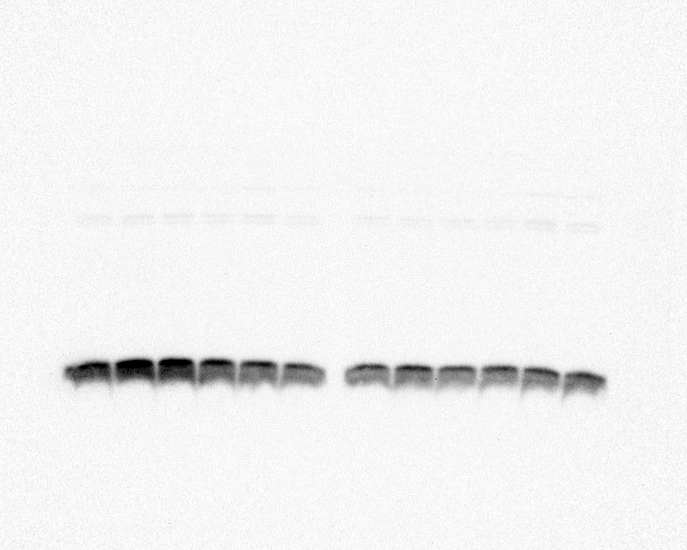

Supplement: Supplementary file 6 — Source data Fig. 3 [file 44318_2026_753_MOESM6_ESM.zip › Figure 3/3A/replicate 3/WB UFM1 lower exposure replicate 3.tif]

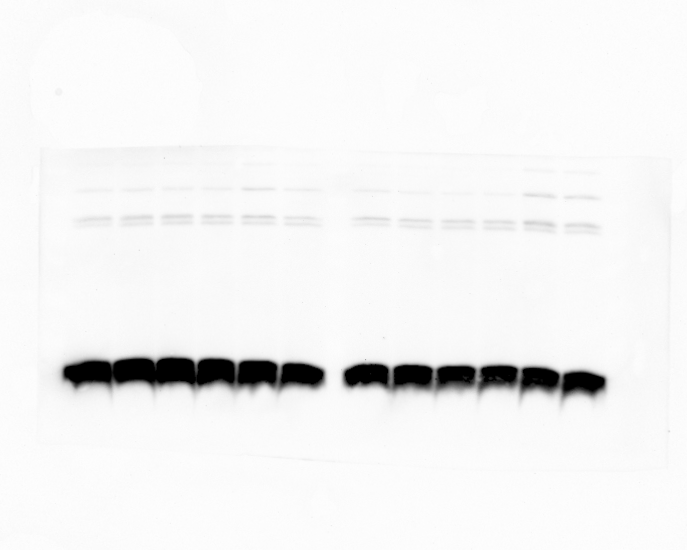

Supplement: Supplementary file 6 — Source data Fig. 3 [file 44318_2026_753_MOESM6_ESM.zip › Figure 3/3A/replicate 3/WB UFM1 replicate 3.tif]

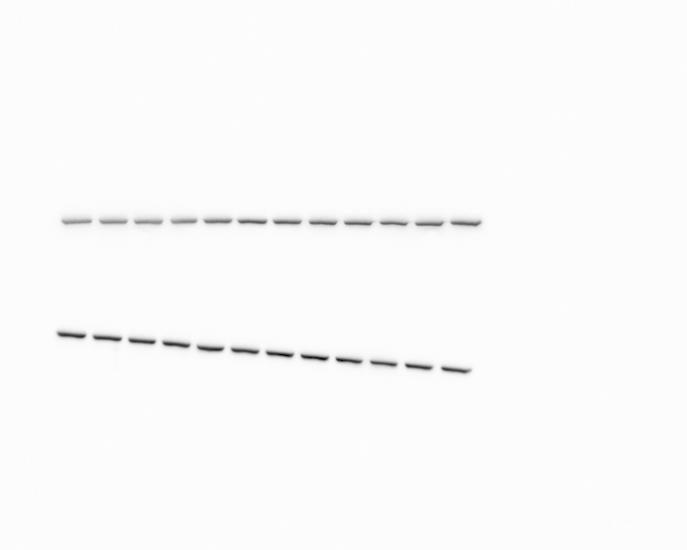

Supplement: Supplementary file 6 — Source data Fig. 3 [file 44318_2026_753_MOESM6_ESM.zip › Figure 3/3A/WB EEF2 replicates 1 upper and 2 lower.tif]

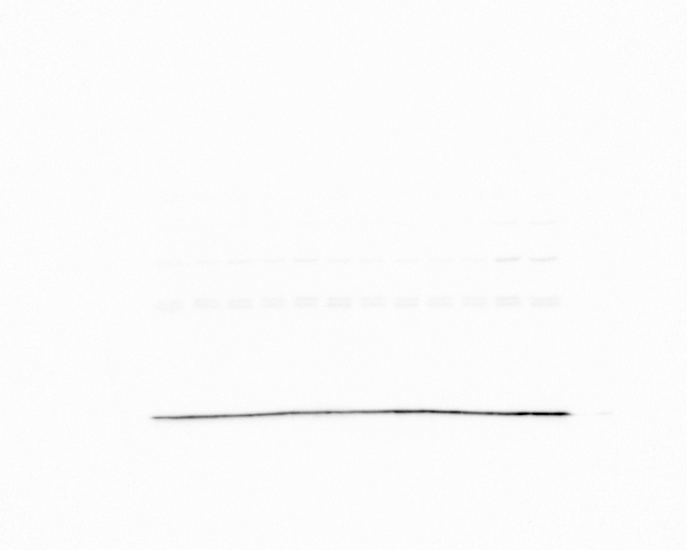

Supplement: Supplementary file 6 — Source data Fig. 3 [file 44318_2026_753_MOESM6_ESM.zip › Figure 3/3A/WB UFM1 lower exposure.tif]

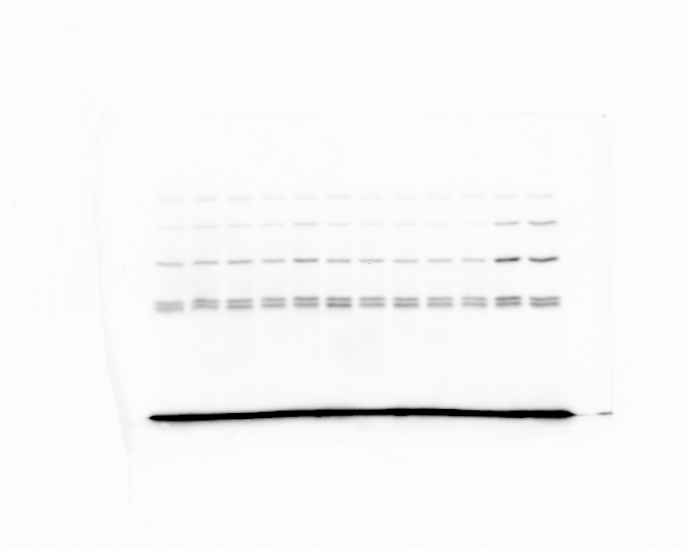

Supplement: Supplementary file 6 — Source data Fig. 3 [file 44318_2026_753_MOESM6_ESM.zip › Figure 3/3A/WB UFM1.tif]

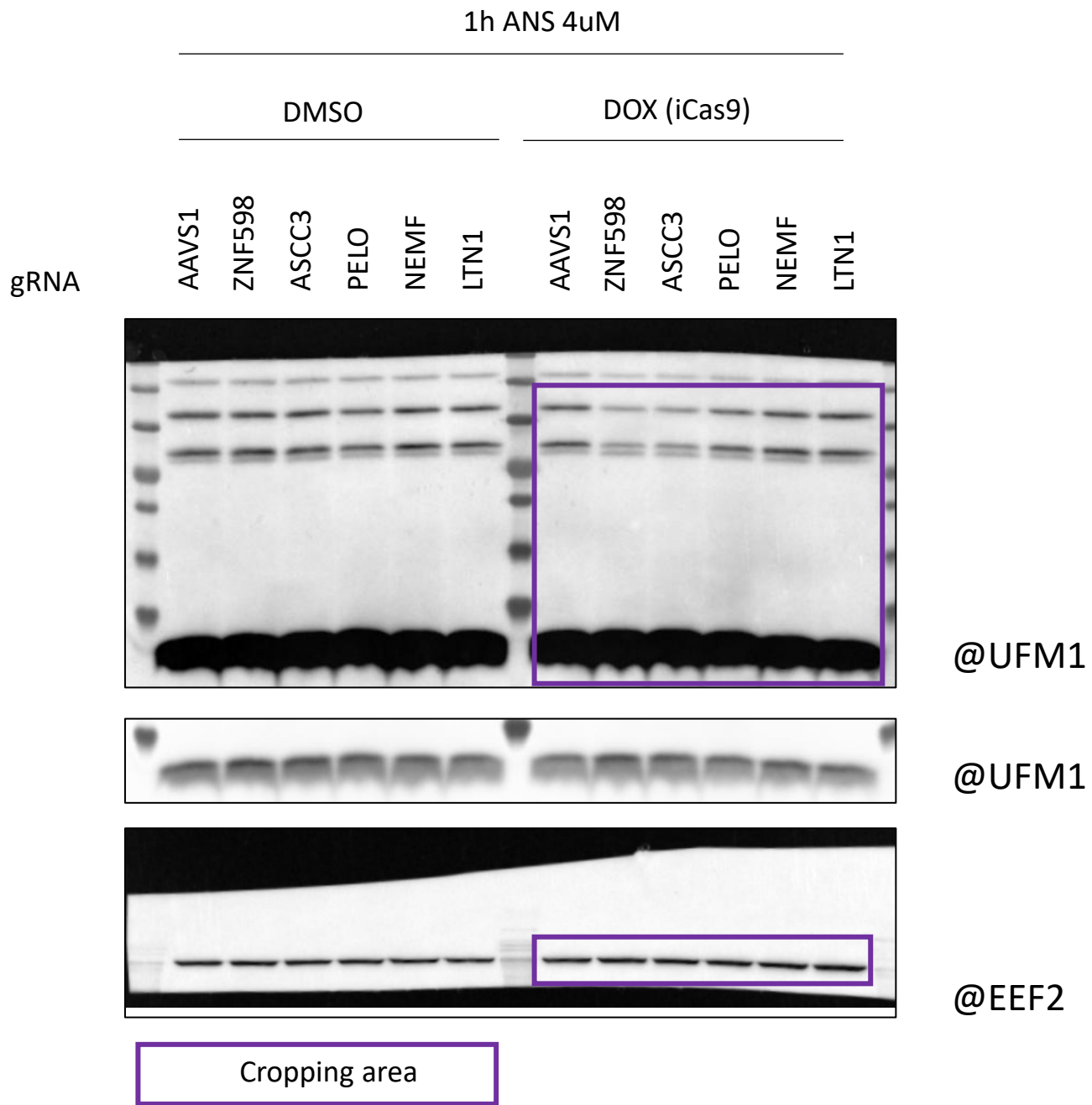

Supplement: Supplementary file 6 — Source data Fig. 3 [file 44318_2026_753_MOESM6_ESM.zip › Figure 3/3C/Fig3C Western Blot labeling replicate 1.pdf]

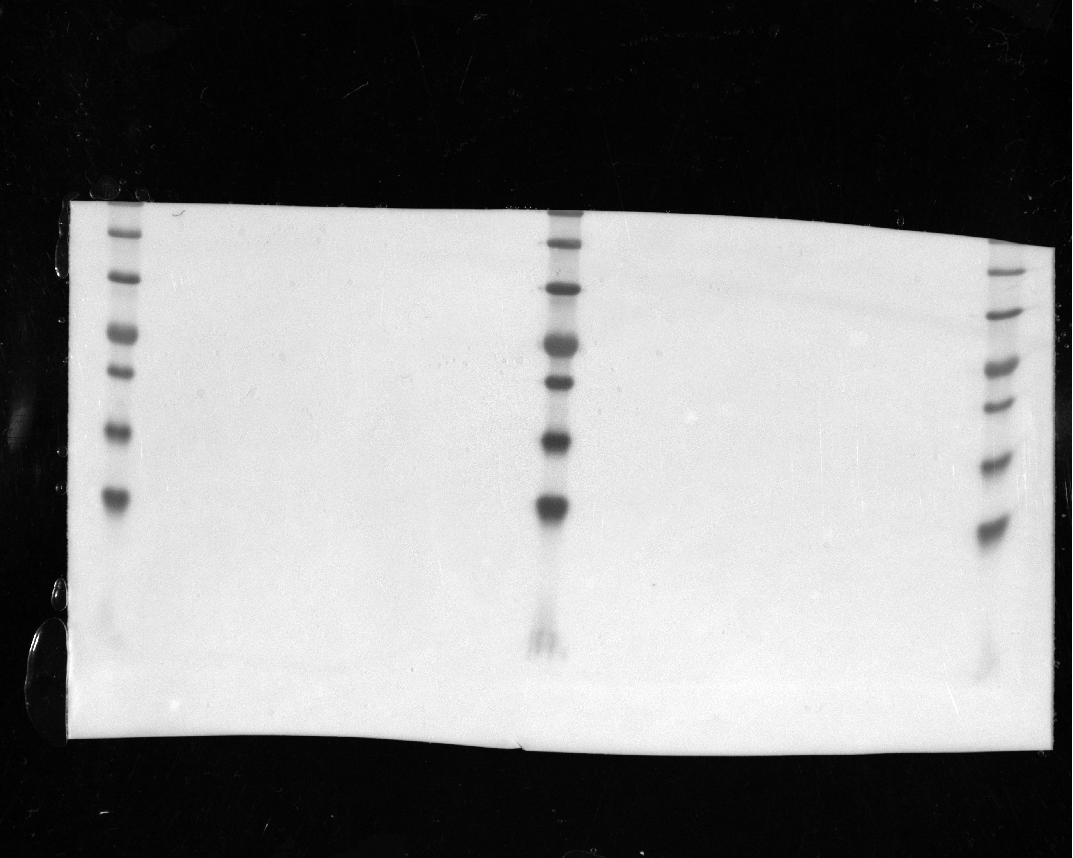

Supplement: Supplementary file 6 — Source data Fig. 3 [file 44318_2026_753_MOESM6_ESM.zip › Figure 3/3C/Membrane UFM1.tif]

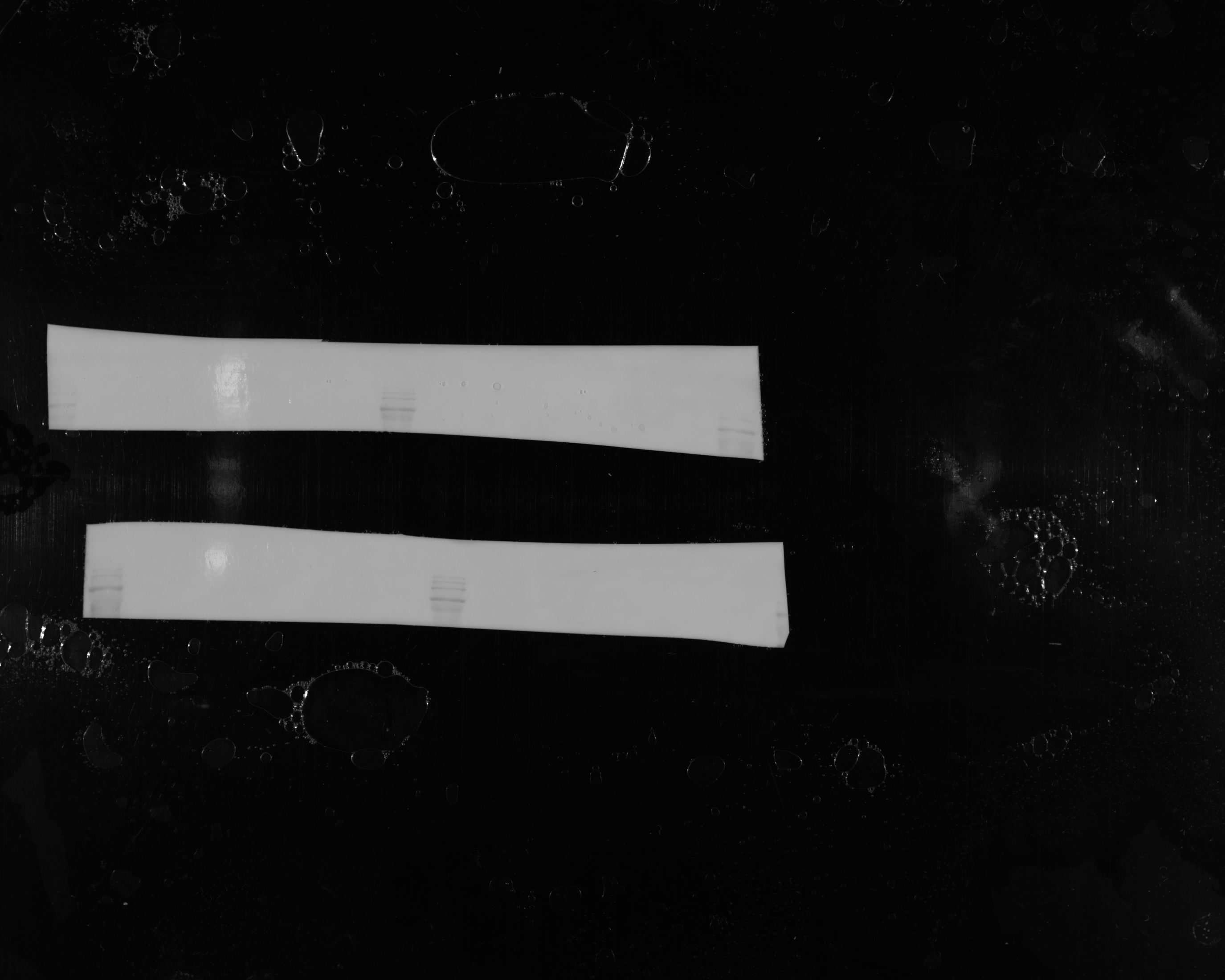

Supplement: Supplementary file 6 — Source data Fig. 3 [file 44318_2026_753_MOESM6_ESM.zip › Figure 3/3C/replicate 2/Membrane EEF2 replicate 2 upper.tif]

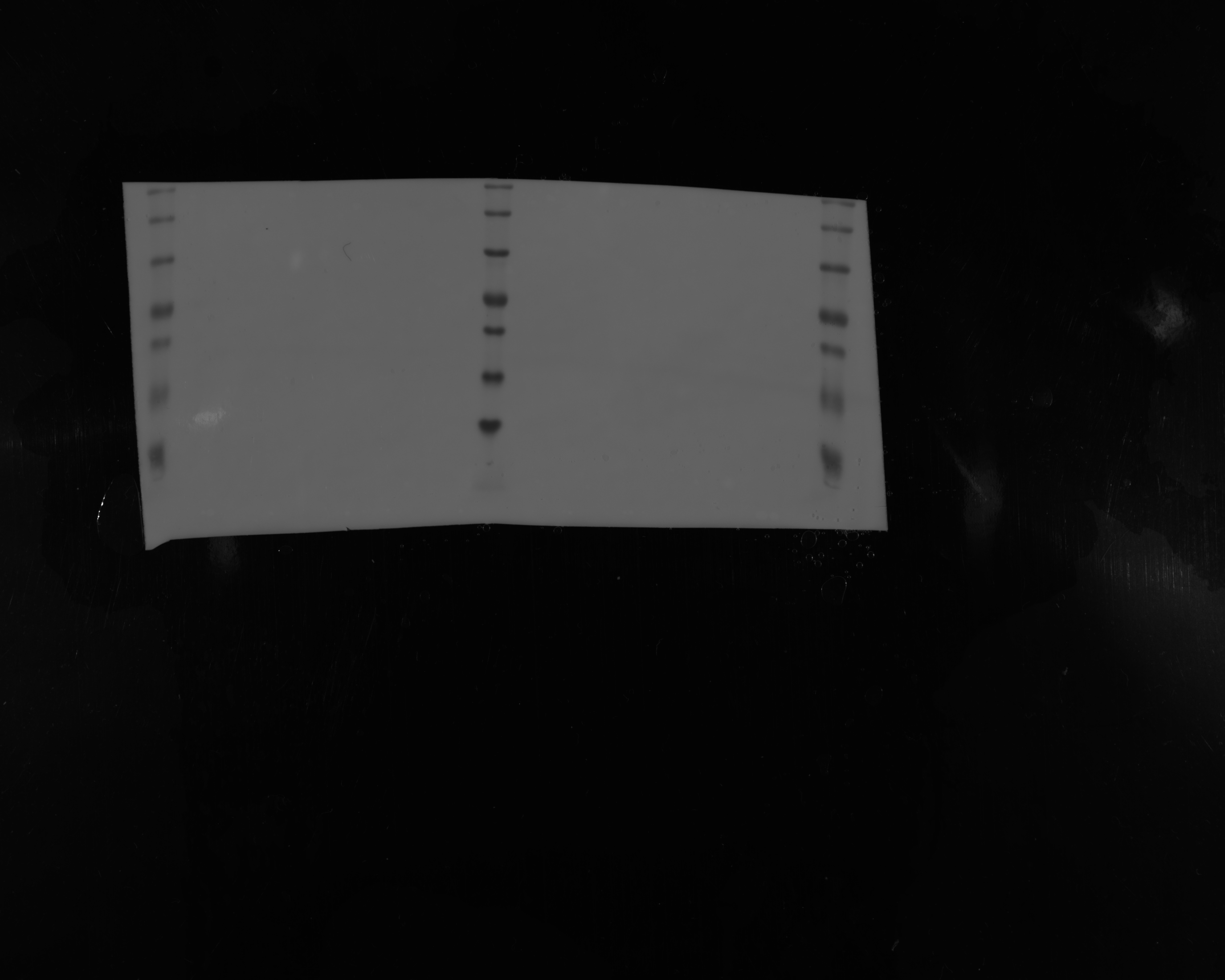

Supplement: Supplementary file 6 — Source data Fig. 3 [file 44318_2026_753_MOESM6_ESM.zip › Figure 3/3C/replicate 2/Membrane UFM1 replicate 2.tif]

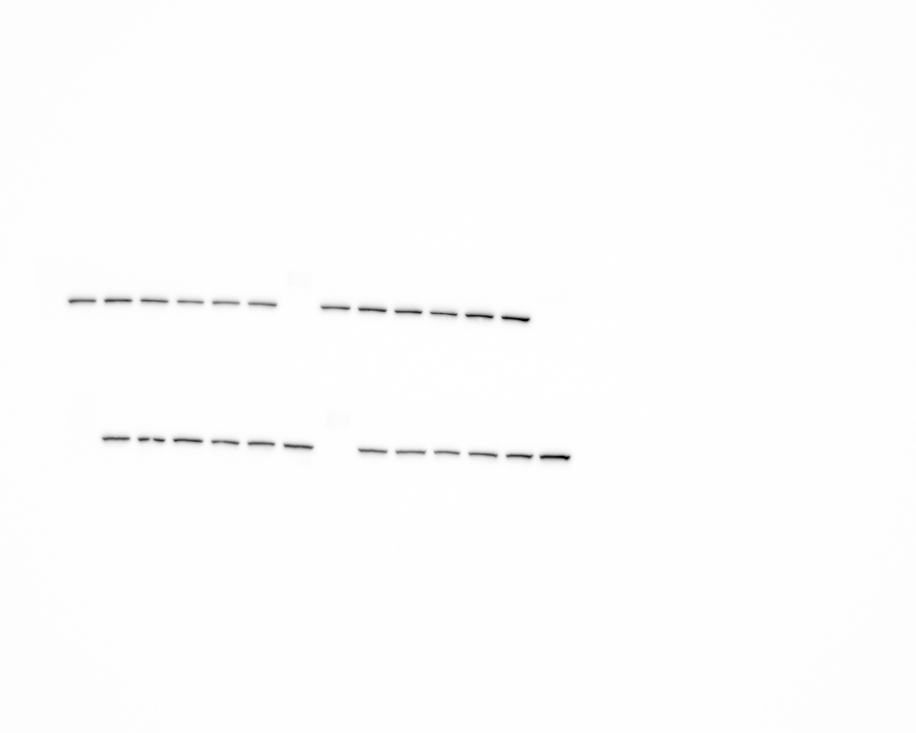

Supplement: Supplementary file 6 — Source data Fig. 3 [file 44318_2026_753_MOESM6_ESM.zip › Figure 3/3C/replicate 2/WB EEF2 replicate 2 upper.tif]

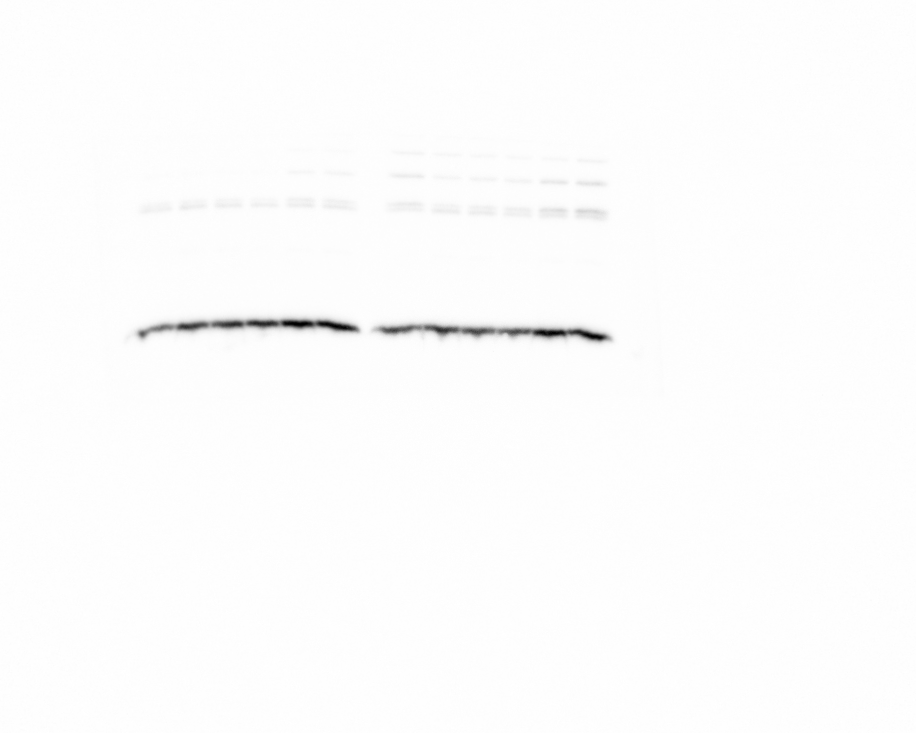

Supplement: Supplementary file 6 — Source data Fig. 3 [file 44318_2026_753_MOESM6_ESM.zip › Figure 3/3C/replicate 2/WB UFM1 lower exposure replicate 2.tif]

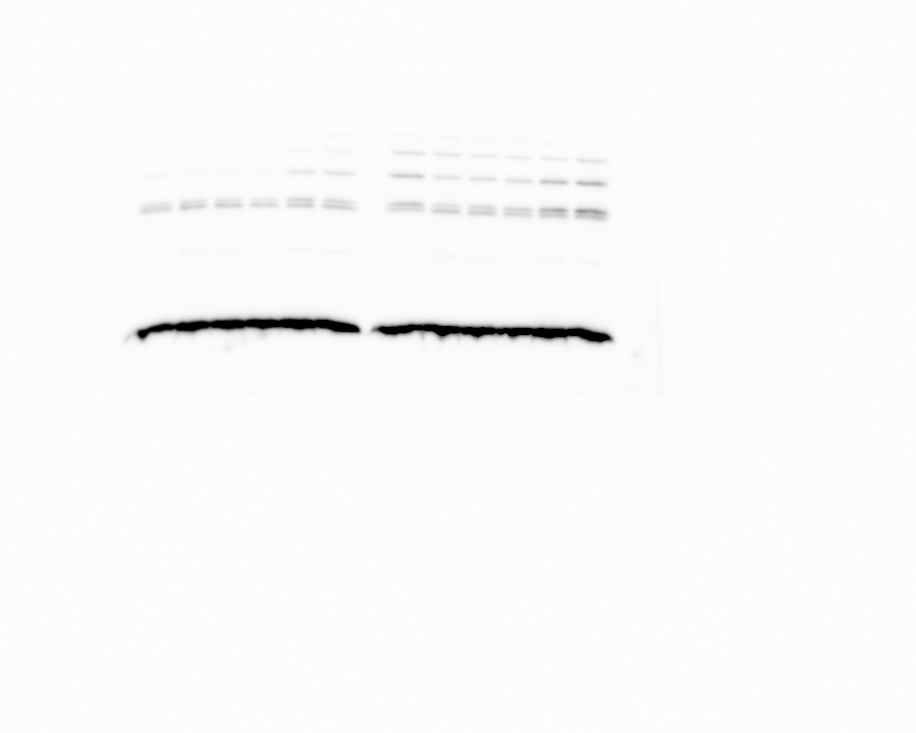

Supplement: Supplementary file 6 — Source data Fig. 3 [file 44318_2026_753_MOESM6_ESM.zip › Figure 3/3C/replicate 2/WB UFM1 replicate 2.tif]

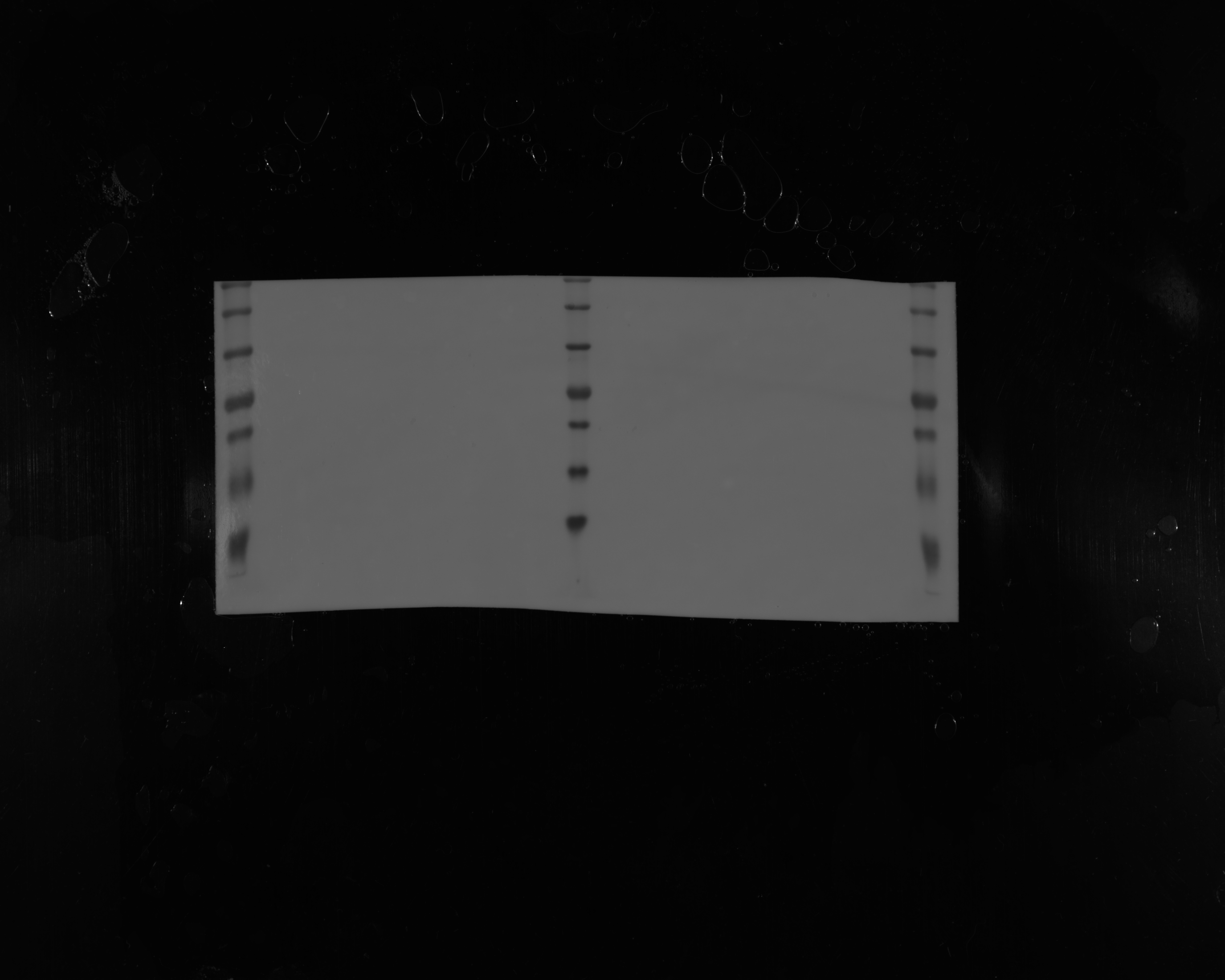

Supplement: Supplementary file 6 — Source data Fig. 3 [file 44318_2026_753_MOESM6_ESM.zip › Figure 3/3C/replicate 3/Membrane UFM1 replicate 3.tif]

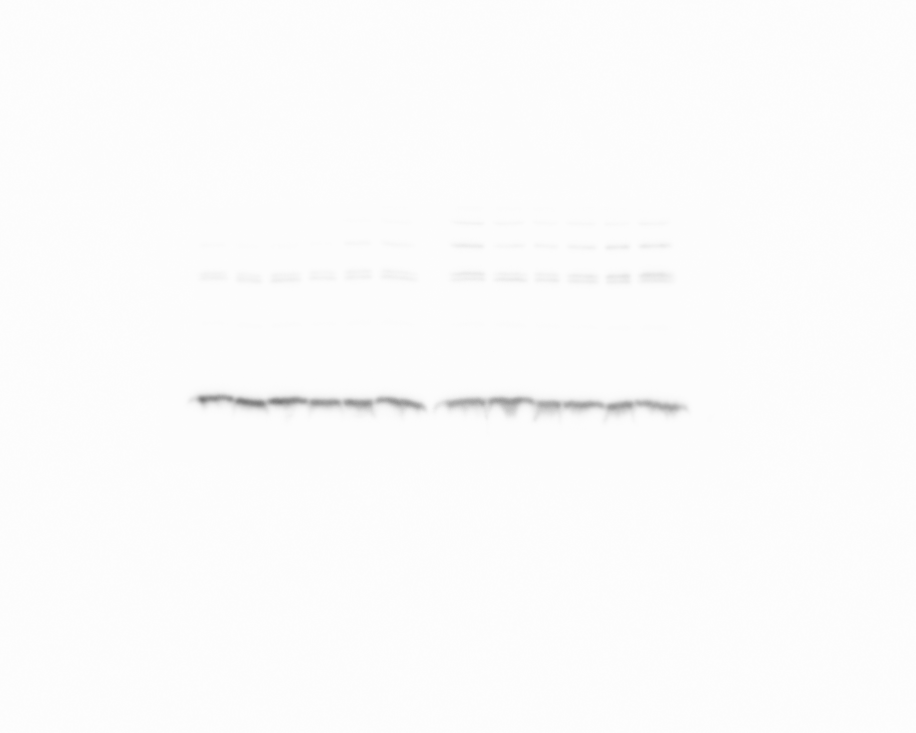

Supplement: Supplementary file 6 — Source data Fig. 3 [file 44318_2026_753_MOESM6_ESM.zip › Figure 3/3C/replicate 3/WB UFM1 lower exposure replicate 3.tif]

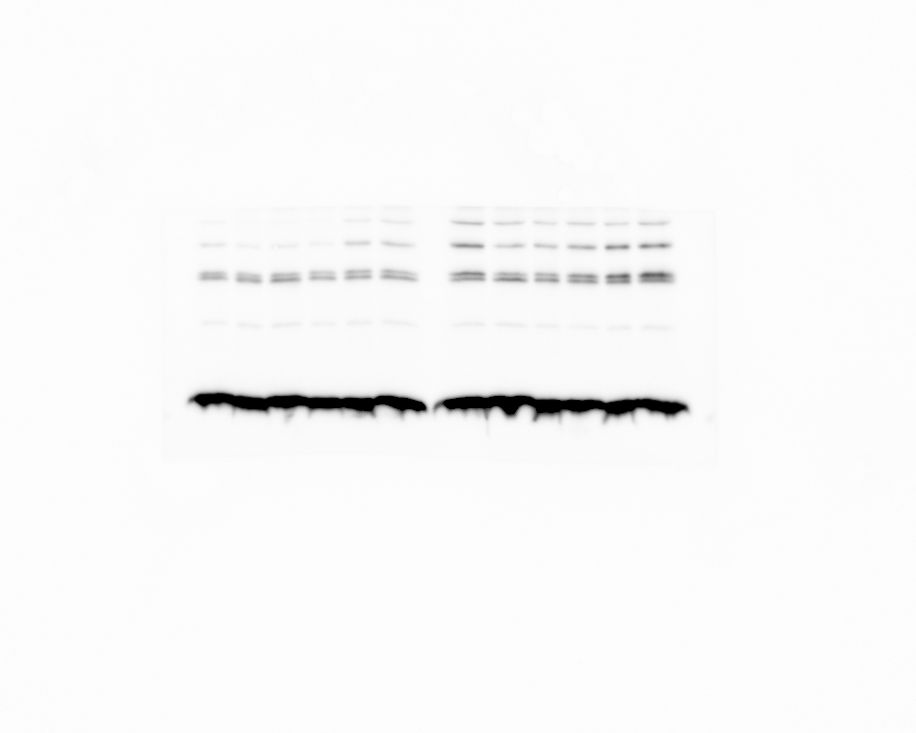

Supplement: Supplementary file 6 — Source data Fig. 3 [file 44318_2026_753_MOESM6_ESM.zip › Figure 3/3C/replicate 3/WB UFM1 replicate 3.tif]

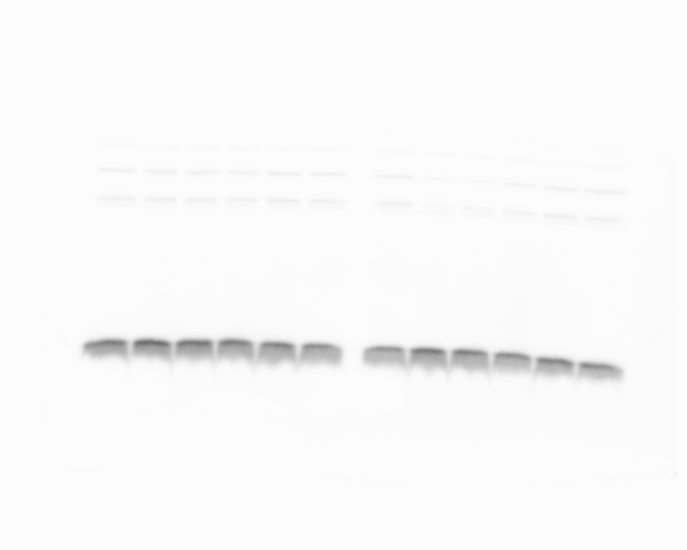

Supplement: Supplementary file 6 — Source data Fig. 3 [file 44318_2026_753_MOESM6_ESM.zip › Figure 3/3C/WB UFM1 lower exposure.tif]

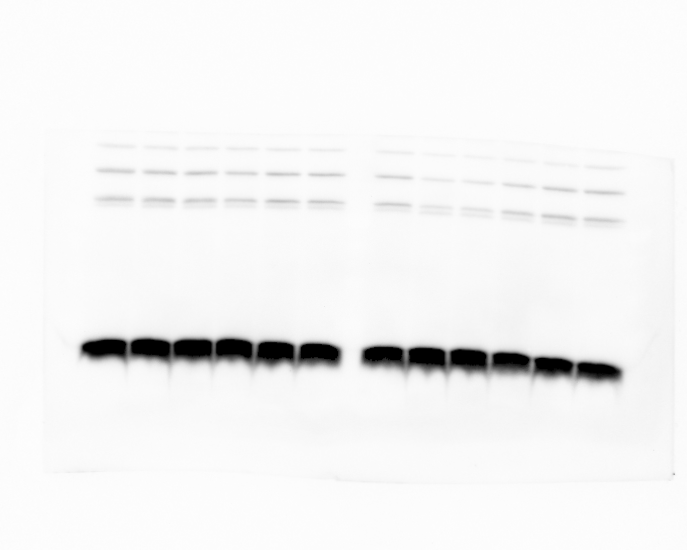

Supplement: Supplementary file 6 — Source data Fig. 3 [file 44318_2026_753_MOESM6_ESM.zip › Figure 3/3C/WB UFM1.tif]

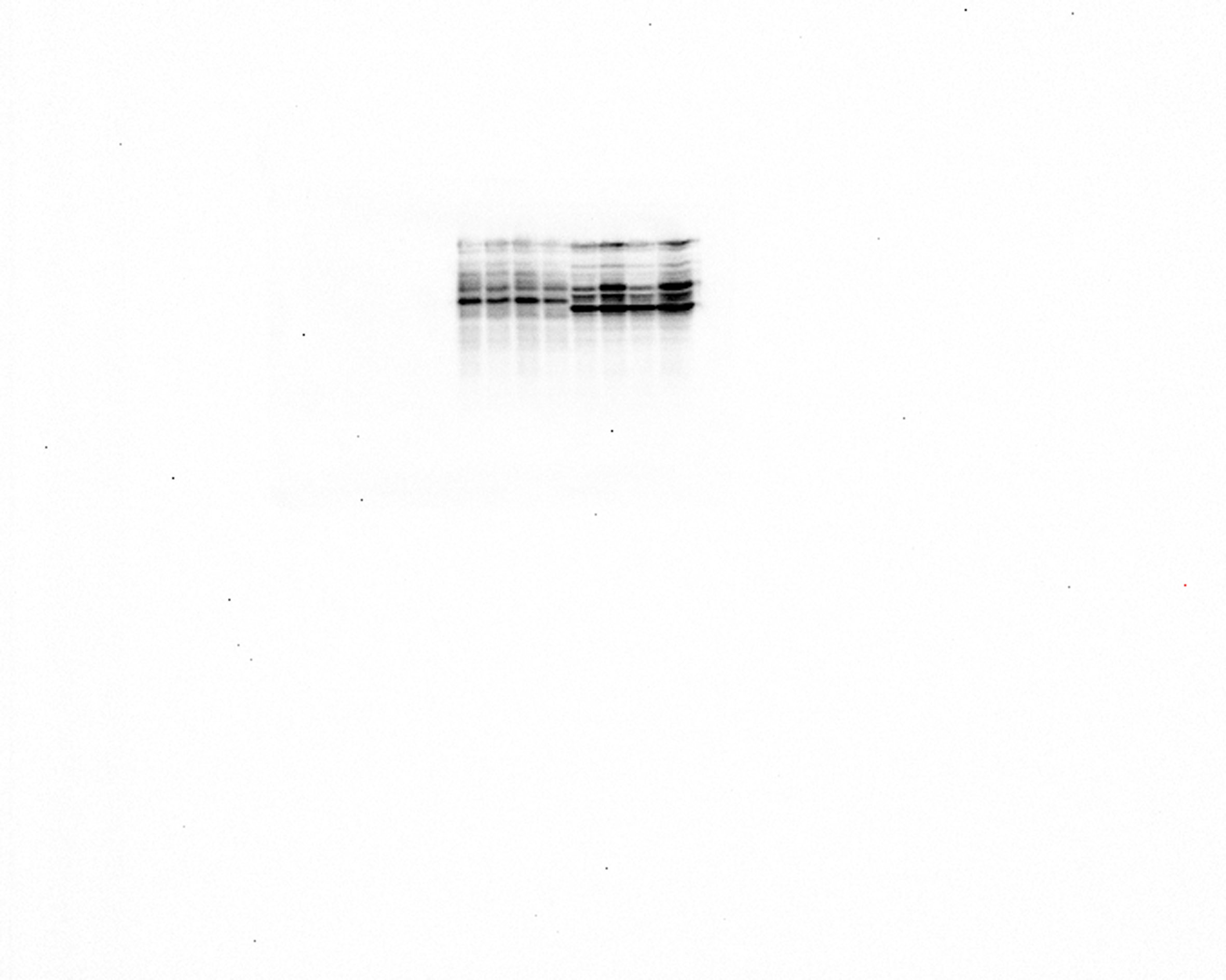

Supplement: Supplementary file 6 — Source data Fig. 3 [file 44318_2026_753_MOESM6_ESM.zip › Figure 3/3E/FLAG/CHEMI_03282023_152900_(Chemi).tif]

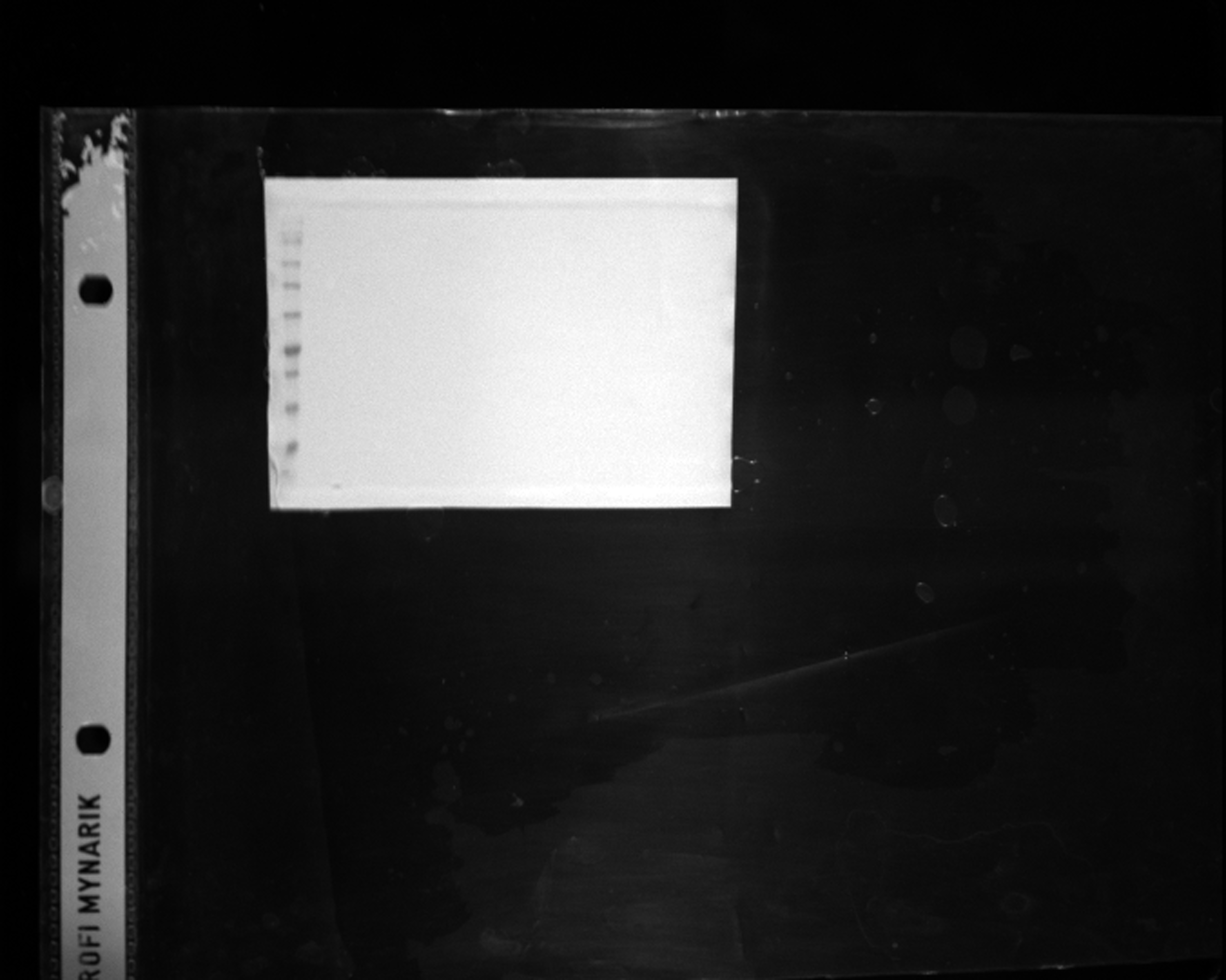

Supplement: Supplementary file 6 — Source data Fig. 3 [file 44318_2026_753_MOESM6_ESM.zip › Figure 3/3E/FLAG/CHEMI_03282023_152900_(Membrane).tif]

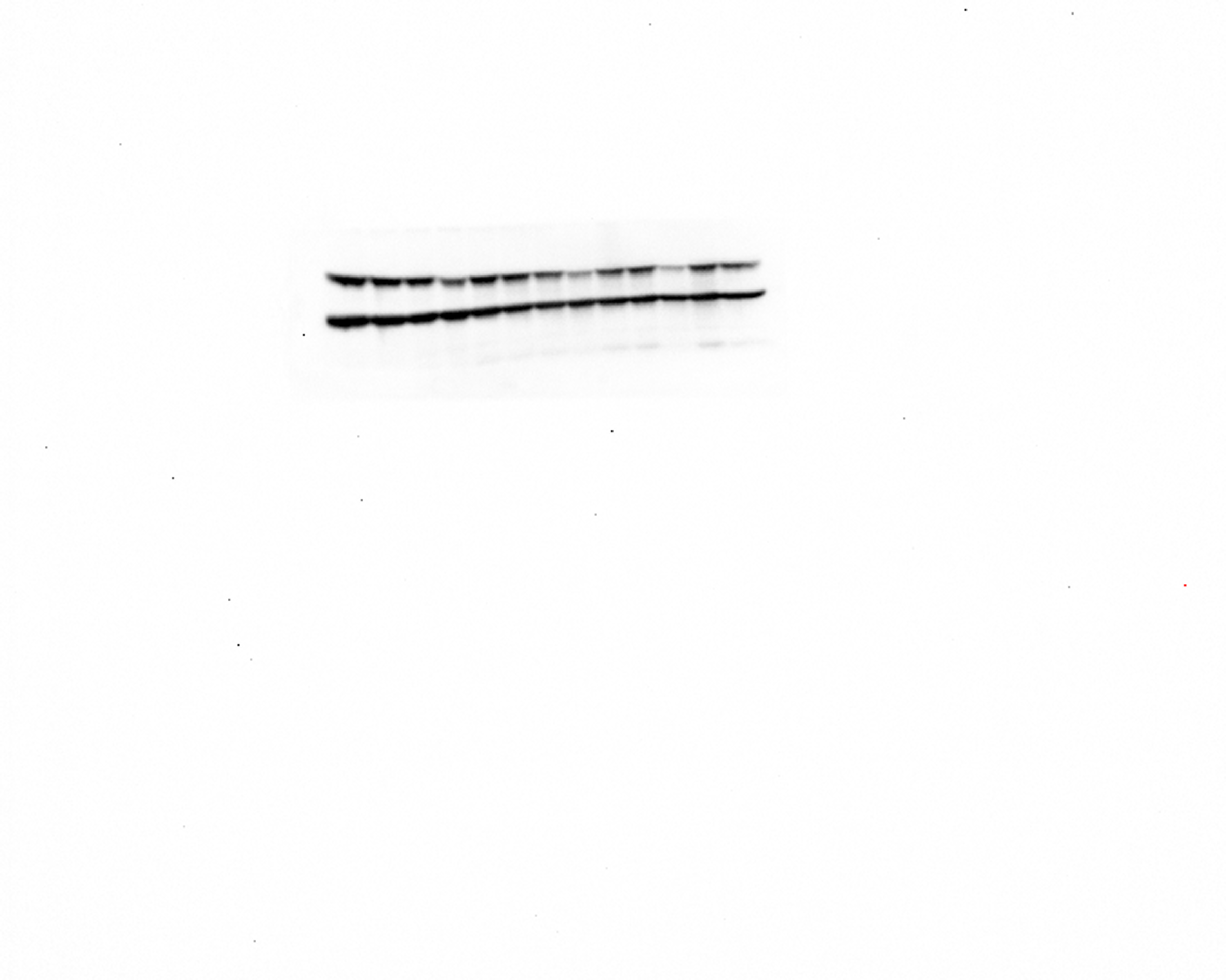

Supplement: Supplementary file 6 — Source data Fig. 3 [file 44318_2026_753_MOESM6_ESM.zip › Figure 3/3E/GAPDH/CHEMI_03292023_132407_(Chemi).tif]

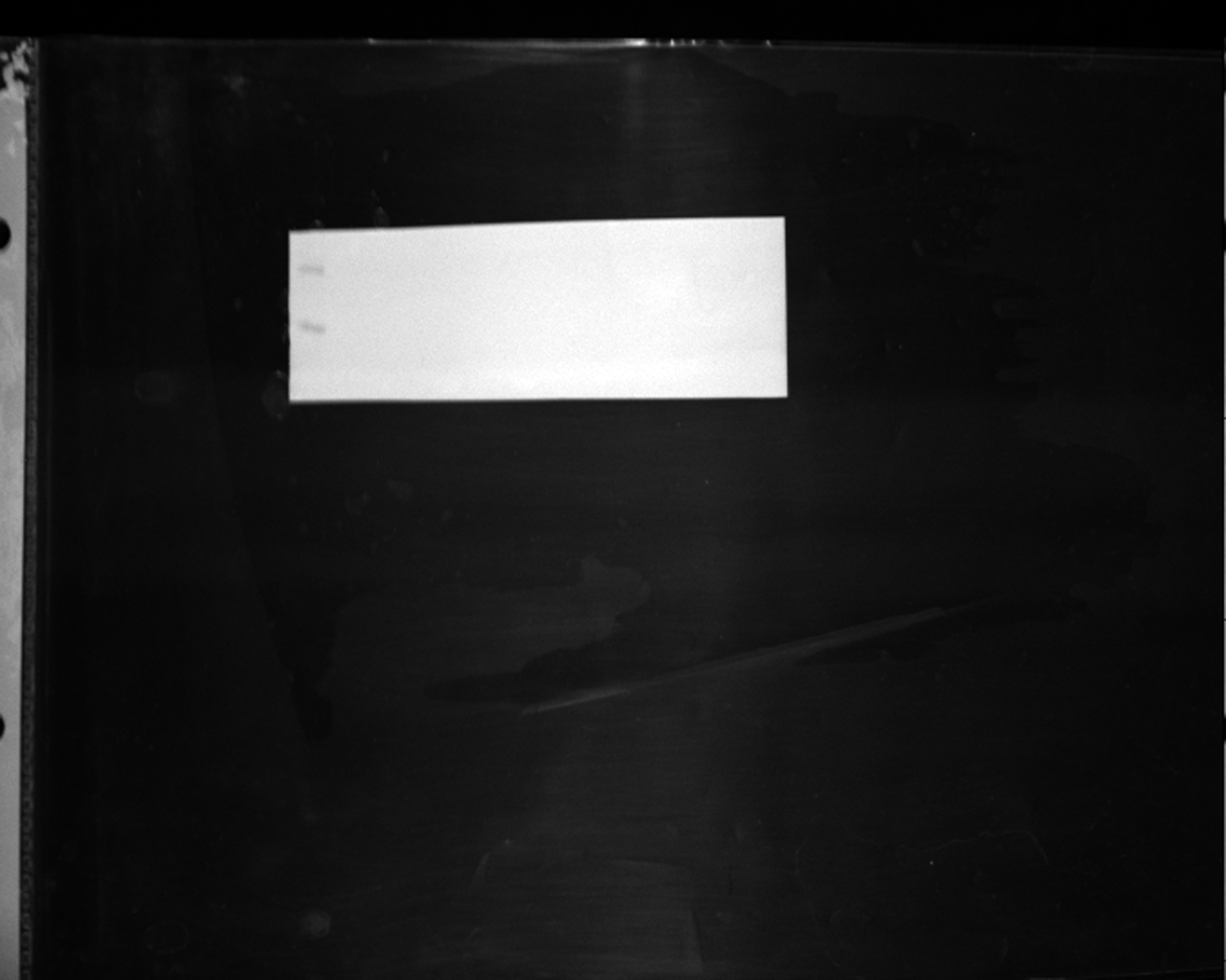

Supplement: Supplementary file 6 — Source data Fig. 3 [file 44318_2026_753_MOESM6_ESM.zip › Figure 3/3E/GAPDH/CHEMI_03292023_132407_(Membrane).tif]

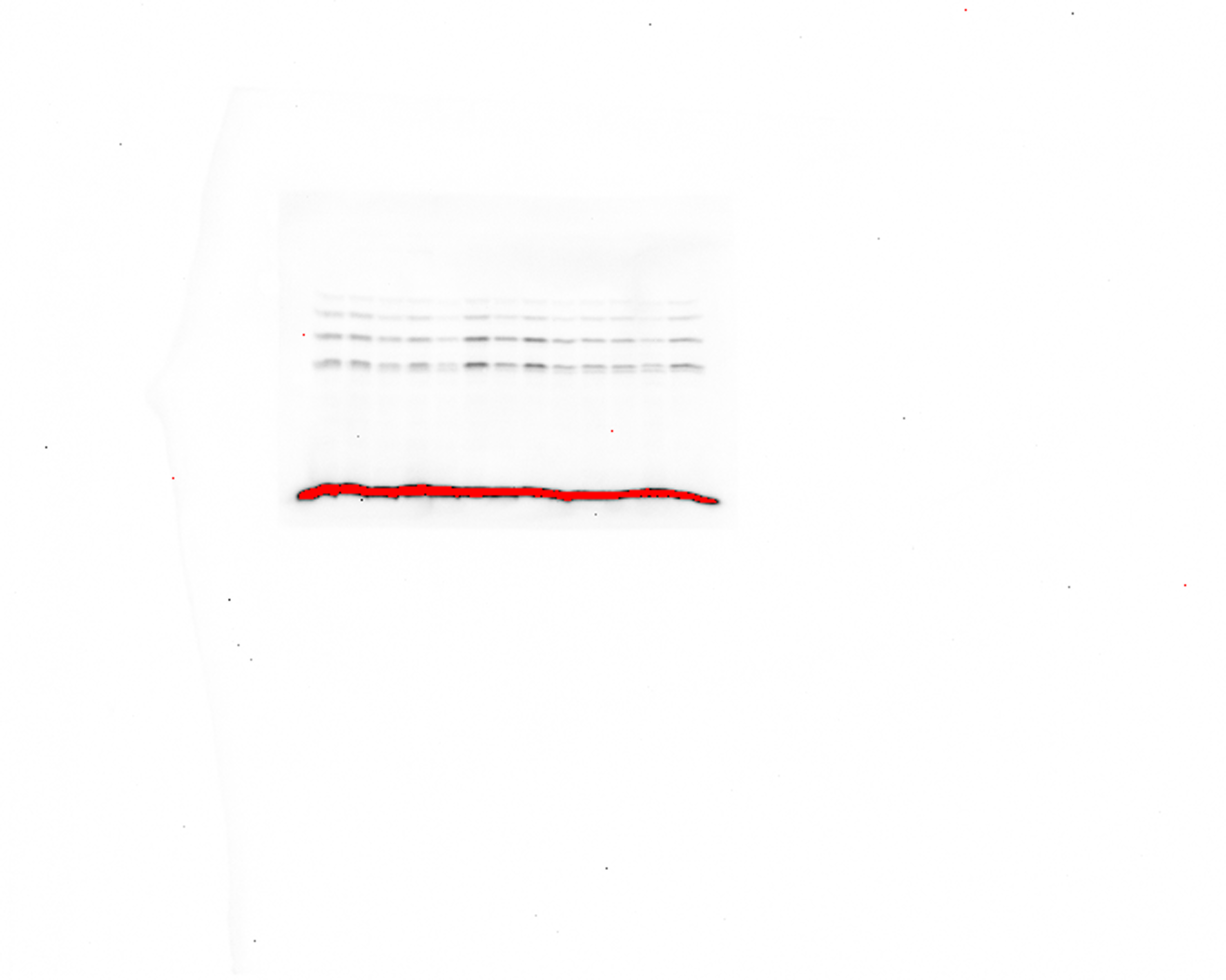

Supplement: Supplementary file 6 — Source data Fig. 3 [file 44318_2026_753_MOESM6_ESM.zip › Figure 3/3E/UFM1/CHEMI_03282023_153658_(Chemi).tif]

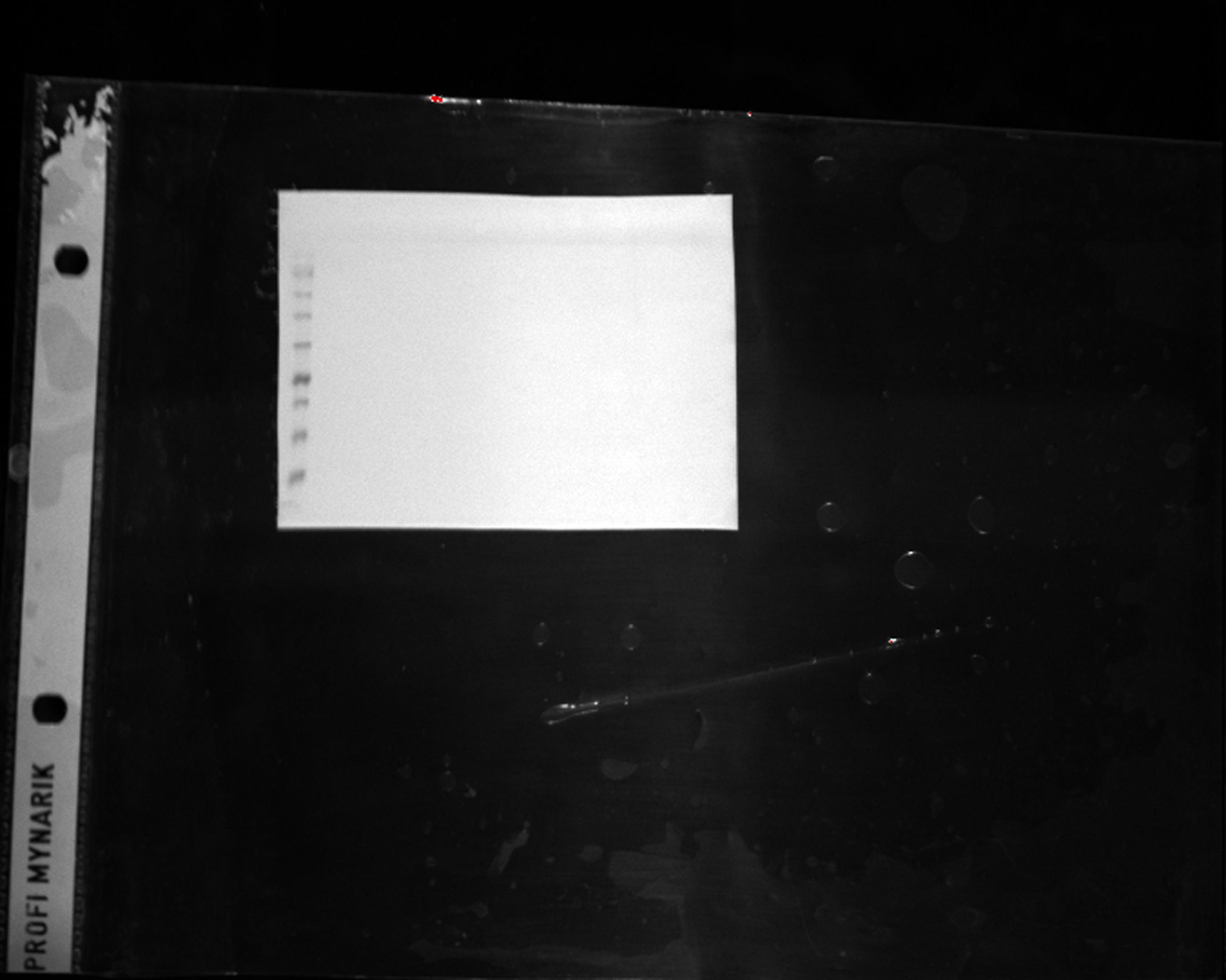

Supplement: Supplementary file 6 — Source data Fig. 3 [file 44318_2026_753_MOESM6_ESM.zip › Figure 3/3E/UFM1/CHEMI_03282023_153658_(Membrane).tif]

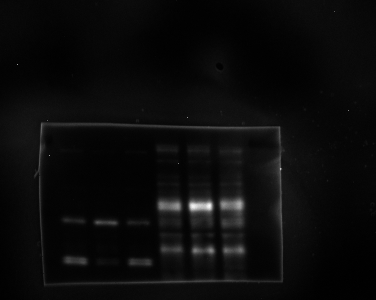

Supplement: Supplementary file 6 — Source data Fig. 3 [file 44318_2026_753_MOESM6_ESM.zip › Figure 3/3H/GFP/CHEMI_10082024_152737Chemi.tif]

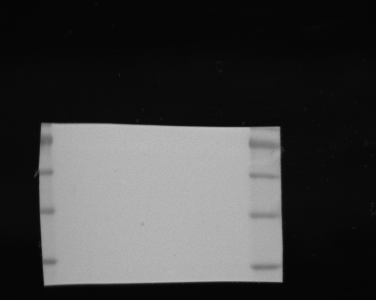

Supplement: Supplementary file 6 — Source data Fig. 3 [file 44318_2026_753_MOESM6_ESM.zip › Figure 3/3H/GFP/CHEMI_10082024_152737Membrane.tif]

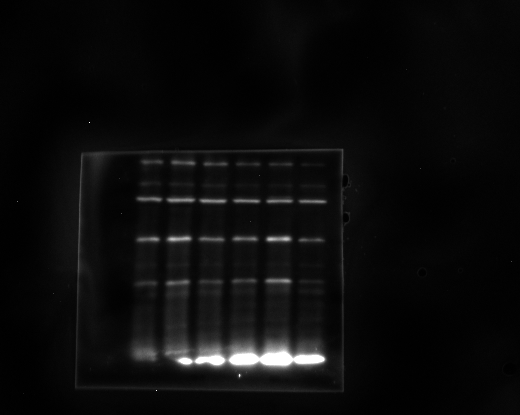

Supplement: Supplementary file 6 — Source data Fig. 3 [file 44318_2026_753_MOESM6_ESM.zip › Figure 3/3H/RPL26 long exposure/CHEMI_10082024_152303Chemi.tif]

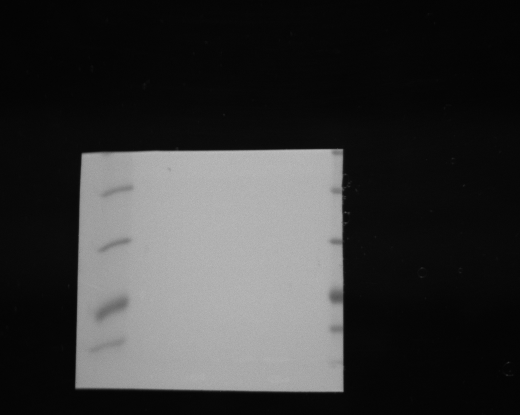

Supplement: Supplementary file 6 — Source data Fig. 3 [file 44318_2026_753_MOESM6_ESM.zip › Figure 3/3H/RPL26 long exposure/CHEMI_10082024_152303Membrane.tif]

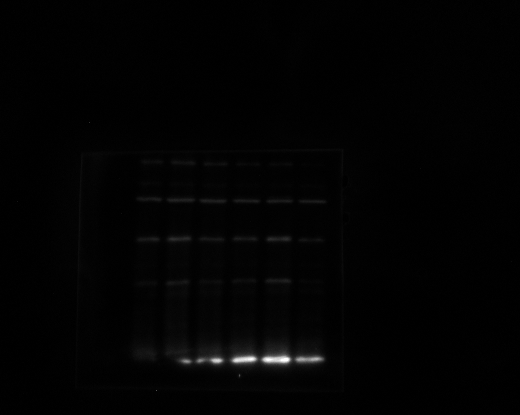

Supplement: Supplementary file 6 — Source data Fig. 3 [file 44318_2026_753_MOESM6_ESM.zip › Figure 3/3H/RPL26 short exposure/CHEMI_10082024_152223Chemi.tif]

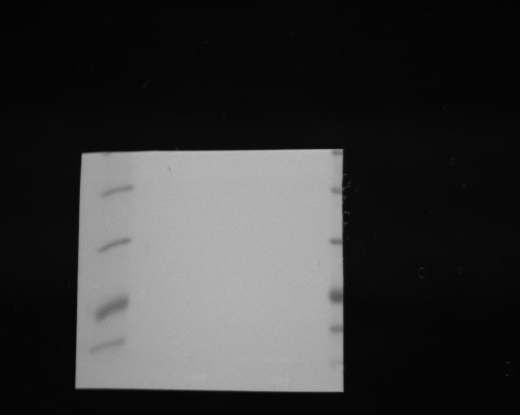

Supplement: Supplementary file 6 — Source data Fig. 3 [file 44318_2026_753_MOESM6_ESM.zip › Figure 3/3H/RPL26 short exposure/CHEMI_10082024_152223Membrane.tif]

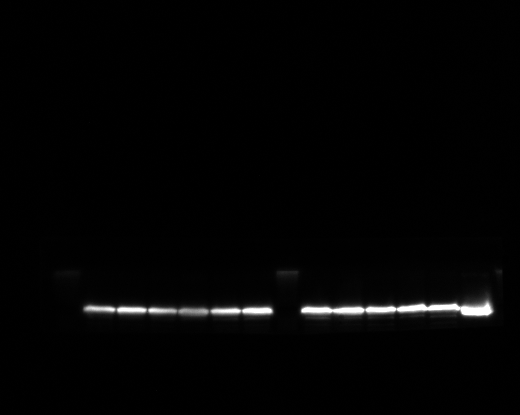

Supplement: Supplementary file 6 — Source data Fig. 3 [file 44318_2026_753_MOESM6_ESM.zip › Figure 3/3J/eEF2/CHEMI_07222025_164305Chemi.tif]

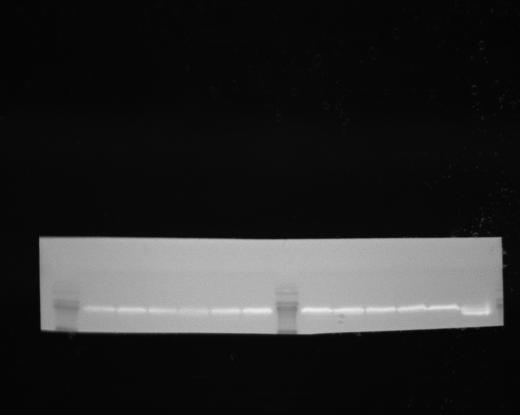

Supplement: Supplementary file 6 — Source data Fig. 3 [file 44318_2026_753_MOESM6_ESM.zip › Figure 3/3J/eEF2/CHEMI_07222025_164305Membrane.tif]

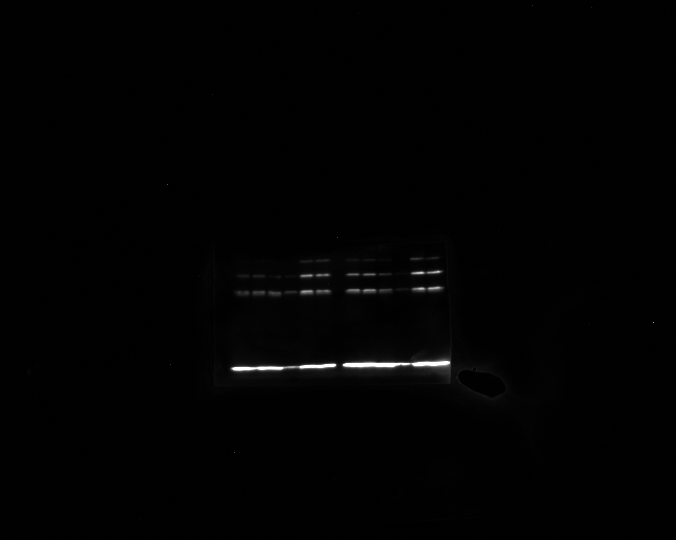

Supplement: Supplementary file 6 — Source data Fig. 3 [file 44318_2026_753_MOESM6_ESM.zip › Figure 3/3J/UFM1/CHEMI_07222025_164055Chemi.tif]

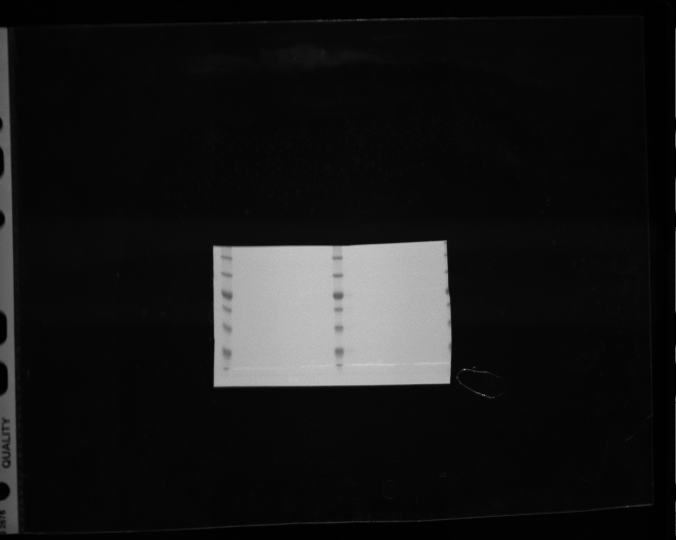

Supplement: Supplementary file 6 — Source data Fig. 3 [file 44318_2026_753_MOESM6_ESM.zip › Figure 3/3J/UFM1/CHEMI_07222025_164055Membrane.tif]

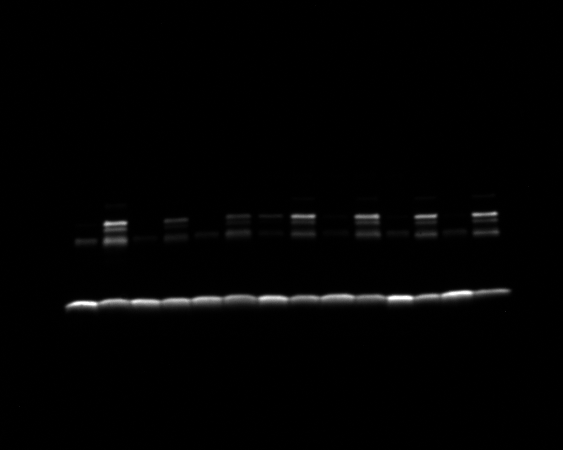

Supplement: Supplementary file 6 — Source data Fig. 3 [file 44318_2026_753_MOESM6_ESM.zip › Figure 3/3L/UFM1/CHEMI_12102024_122007_(Chemi)_raw.tif]

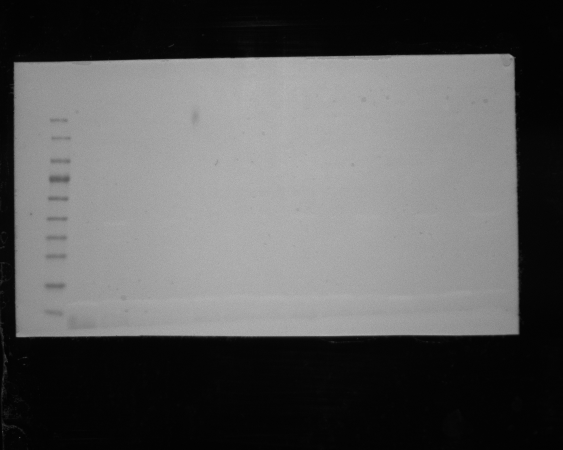

Supplement: Supplementary file 6 — Source data Fig. 3 [file 44318_2026_753_MOESM6_ESM.zip › Figure 3/3L/UFM1/CHEMI_12102024_122007_(Membrane)_raw.tif]

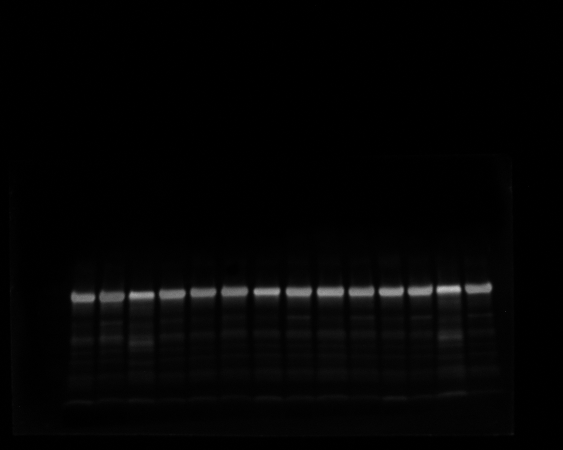

Supplement: Supplementary file 6 — Source data Fig. 3 [file 44318_2026_753_MOESM6_ESM.zip › Figure 3/3L/UGPase/CHEMI_12112024_173716_(Chemi)_raw.tif]

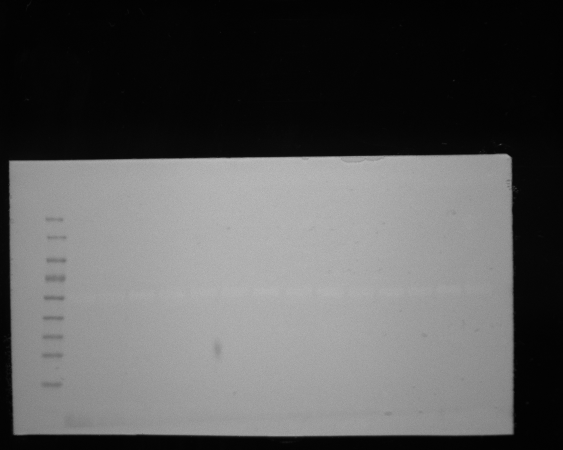

Supplement: Supplementary file 6 — Source data Fig. 3 [file 44318_2026_753_MOESM6_ESM.zip › Figure 3/3L/UGPase/CHEMI_12112024_173716_(Membrane)_raw.tif]

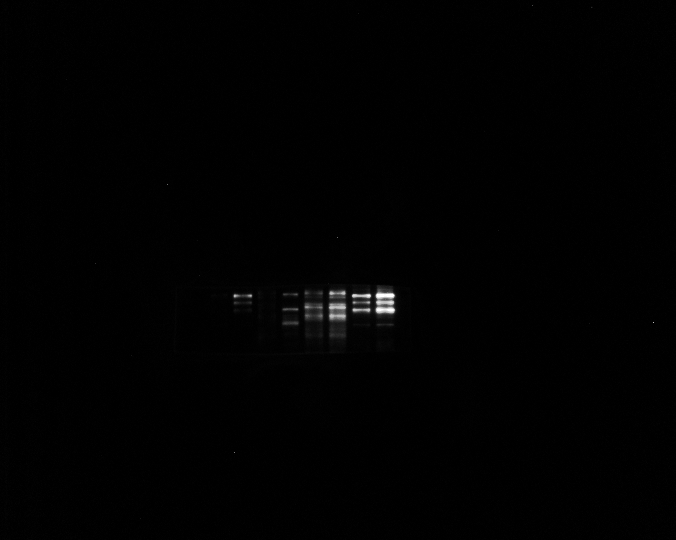

Supplement: Supplementary file 7 — Source data Fig. 4 [file 44318_2026_753_MOESM7_ESM.zip › Figure 4/4D/GFP eluate/CHEMI_10302024_145751_(Chemi)_raw.tif]

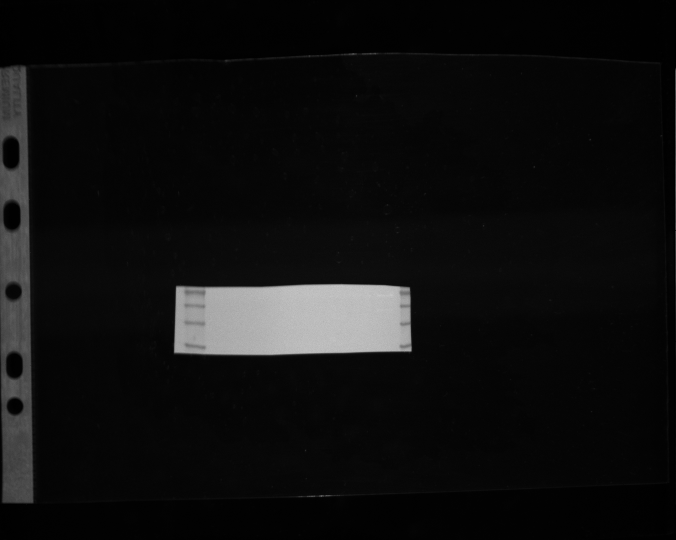

Supplement: Supplementary file 7 — Source data Fig. 4 [file 44318_2026_753_MOESM7_ESM.zip › Figure 4/4D/GFP eluate/CHEMI_10302024_145751_(Membrane)_raw.tif]

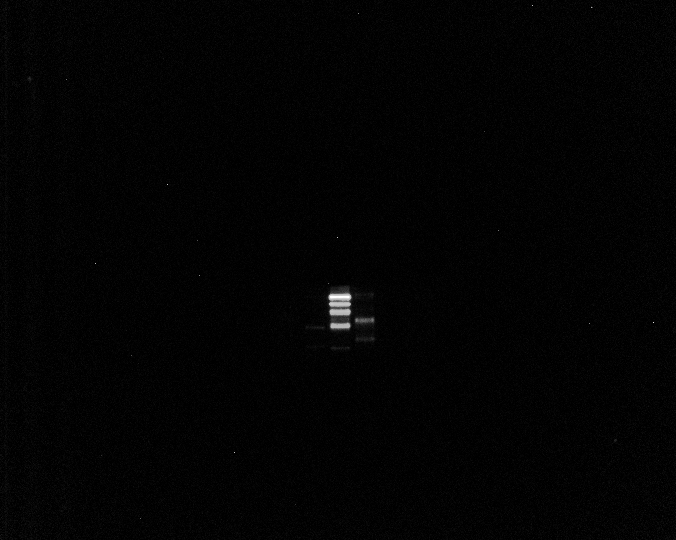

Supplement: Supplementary file 7 — Source data Fig. 4 [file 44318_2026_753_MOESM7_ESM.zip › Figure 4/4D/GFP lysate/CHEMI_10302024_160333_(Chemi)_raw.tif]

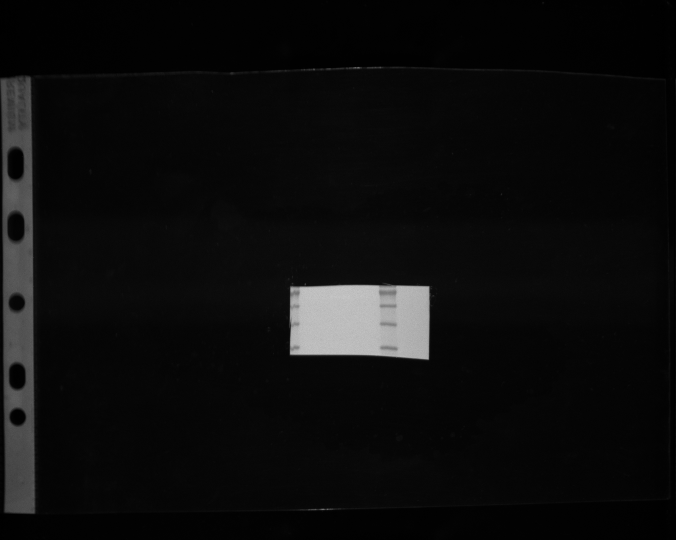

Supplement: Supplementary file 7 — Source data Fig. 4 [file 44318_2026_753_MOESM7_ESM.zip › Figure 4/4D/GFP lysate/CHEMI_10302024_160333_(Membrane)_raw.tif]

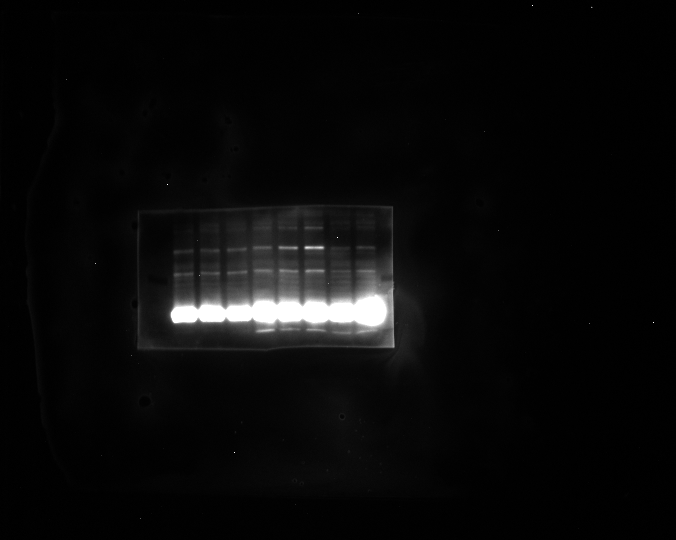

Supplement: Supplementary file 7 — Source data Fig. 4 [file 44318_2026_753_MOESM7_ESM.zip › Figure 4/4D/RPL26 eluate/High exposure/CHEMI_10302024_150458_(Chemi)_raw.tif]

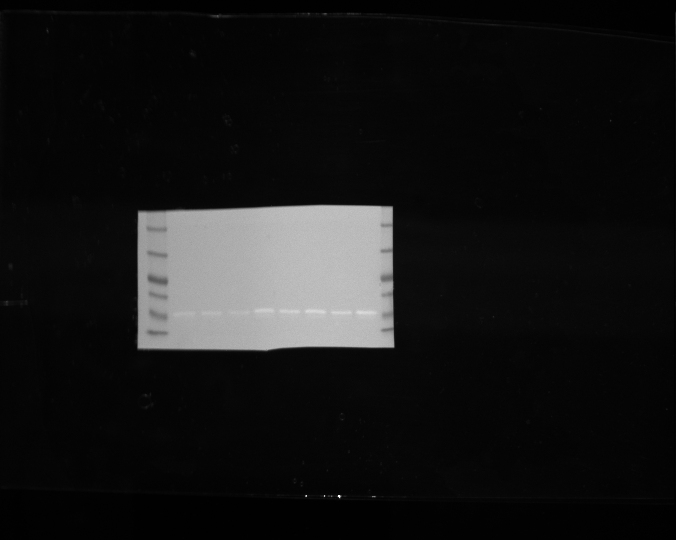

Supplement: Supplementary file 7 — Source data Fig. 4 [file 44318_2026_753_MOESM7_ESM.zip › Figure 4/4D/RPL26 eluate/High exposure/CHEMI_10302024_150458_(Membrane)_raw.tif]
