# Supplementary figures and images for: The coordinated action of UFMylation and the RQC pathways clears arrested polypeptides at the ER (part 2 of 2)
Source: EMBO J. 2026 Mar 25;45(9):3252–75. doi: 10.1038/s44318-026-00753-9 (PMC13144351; doi:10.1038/s44318-026-00753-9)

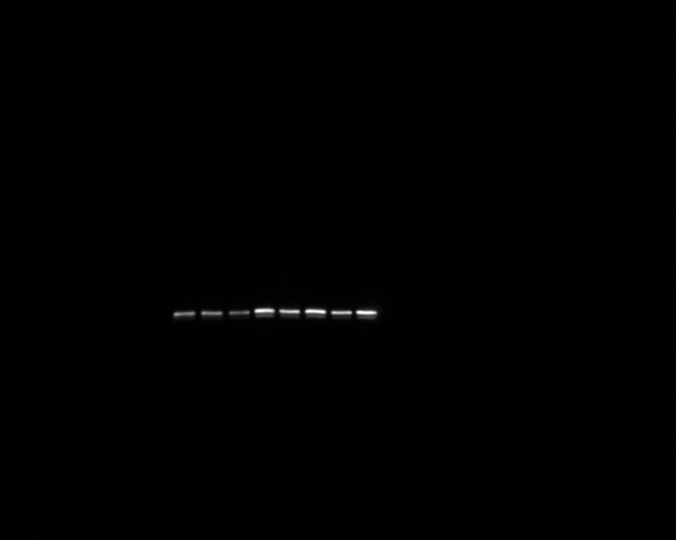

Supplement: Supplementary file 7 — Source data Fig. 4 [file 44318_2026_753_MOESM7_ESM.zip › Figure 4/4D/RPL26 eluate/Low exposure/CHEMI_10302024_150312_(Chemi)_raw.tif]

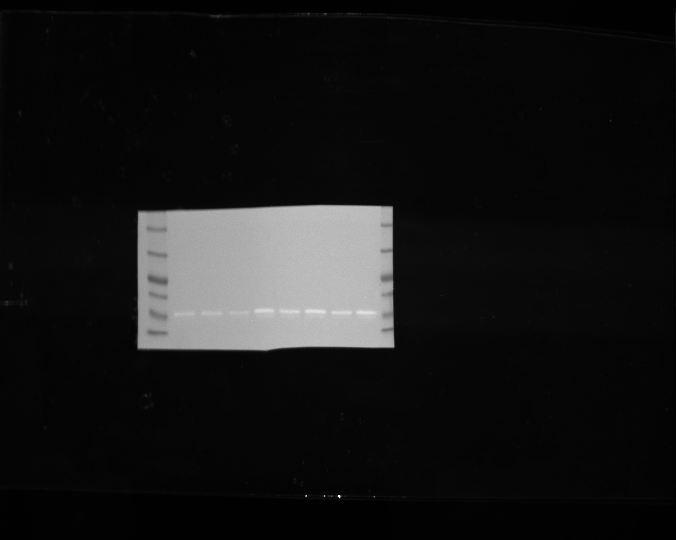

Supplement: Supplementary file 7 — Source data Fig. 4 [file 44318_2026_753_MOESM7_ESM.zip › Figure 4/4D/RPL26 eluate/Low exposure/CHEMI_10302024_150312_(Membrane)_raw.tif]

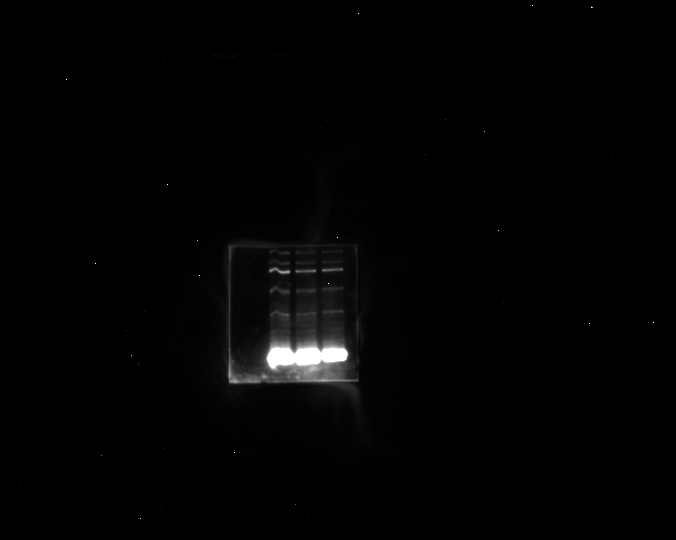

Supplement: Supplementary file 7 — Source data Fig. 4 [file 44318_2026_753_MOESM7_ESM.zip › Figure 4/4D/RPL26 lysate/High exposure/CHEMI_10302024_145036_(Chemi)_raw.tif]

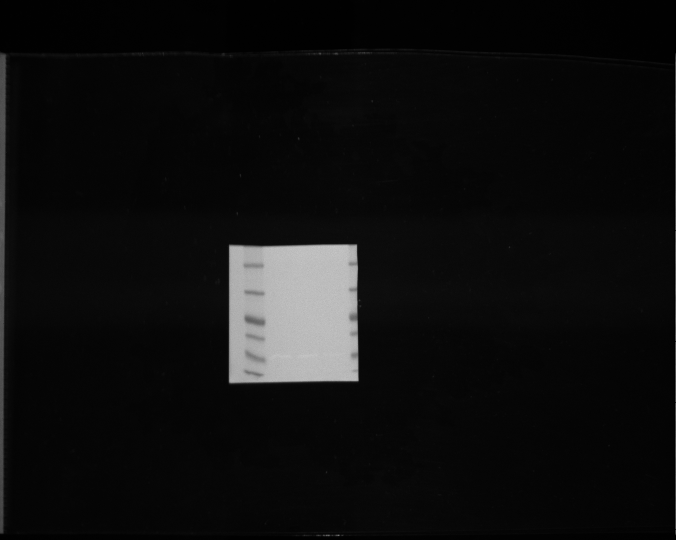

Supplement: Supplementary file 7 — Source data Fig. 4 [file 44318_2026_753_MOESM7_ESM.zip › Figure 4/4D/RPL26 lysate/High exposure/CHEMI_10302024_145036_(Membrane)_raw.tif]

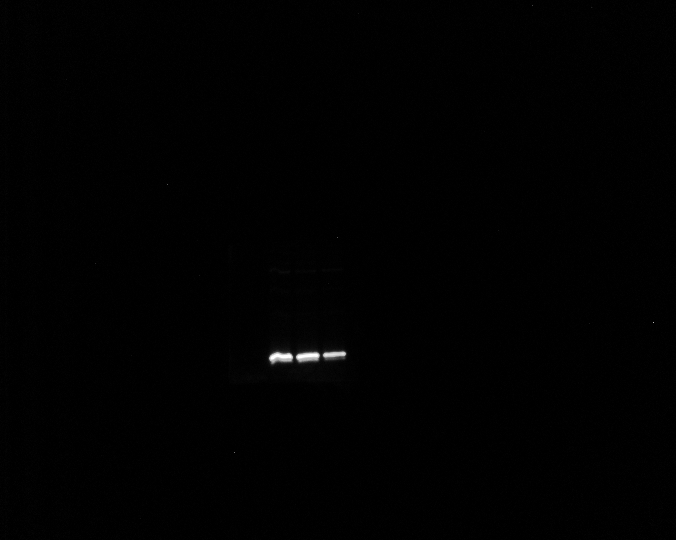

Supplement: Supplementary file 7 — Source data Fig. 4 [file 44318_2026_753_MOESM7_ESM.zip › Figure 4/4D/RPL26 lysate/Low exposure/CHEMI_10302024_144607_(Chemi)_raw.tif]

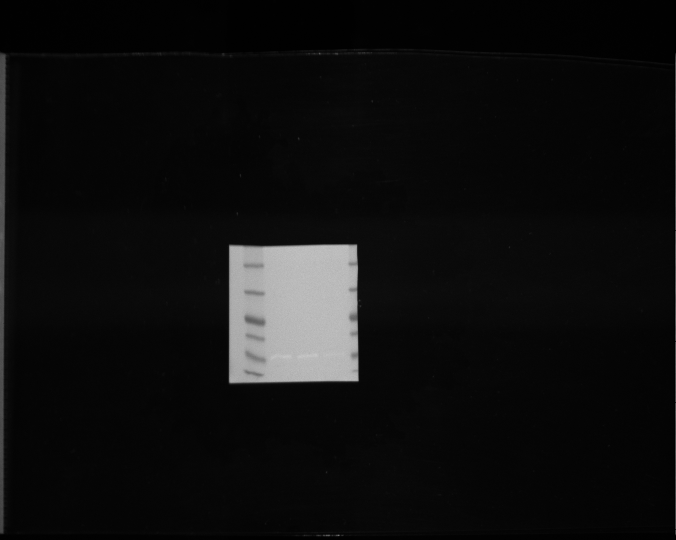

Supplement: Supplementary file 7 — Source data Fig. 4 [file 44318_2026_753_MOESM7_ESM.zip › Figure 4/4D/RPL26 lysate/Low exposure/CHEMI_10302024_144607_(Membrane)_raw.tif]

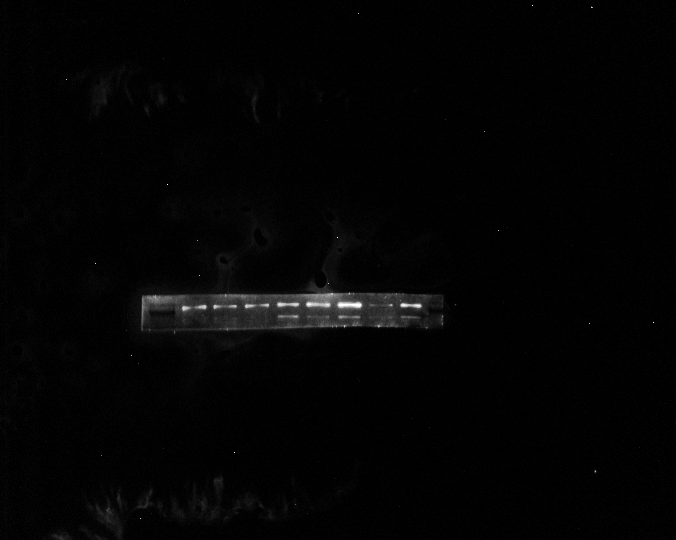

Supplement: Supplementary file 7 — Source data Fig. 4 [file 44318_2026_753_MOESM7_ESM.zip › Figure 4/4D/UFL1 eluate/CHEMI_10302024_151646_(Chemi)_raw.tif]

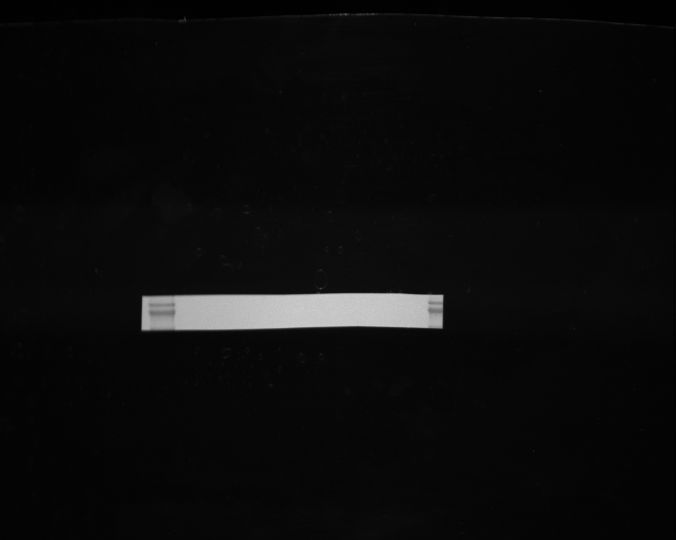

Supplement: Supplementary file 7 — Source data Fig. 4 [file 44318_2026_753_MOESM7_ESM.zip › Figure 4/4D/UFL1 eluate/CHEMI_10302024_151646_(Membrane)_raw.tif]

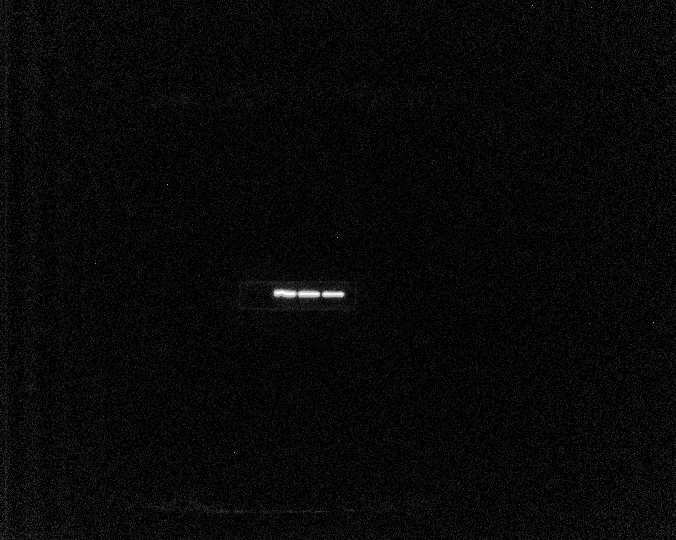

Supplement: Supplementary file 7 — Source data Fig. 4 [file 44318_2026_753_MOESM7_ESM.zip › Figure 4/4D/UFL1 lysate/CHEMI_10302024_154432_(Chemi)_raw.tif]

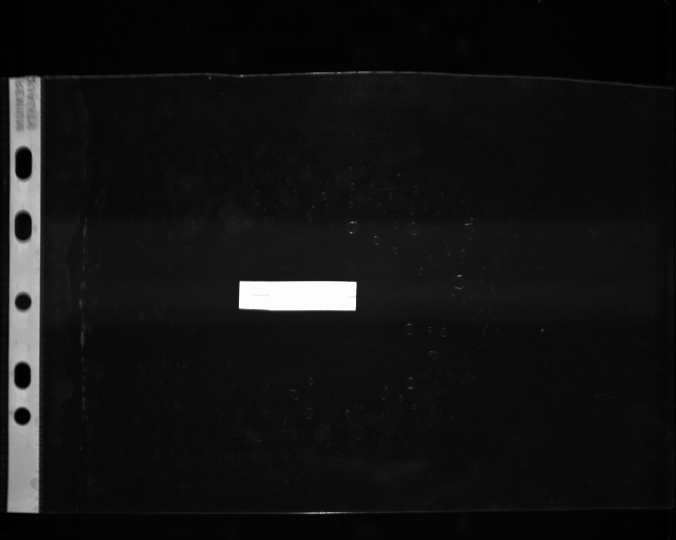

Supplement: Supplementary file 7 — Source data Fig. 4 [file 44318_2026_753_MOESM7_ESM.zip › Figure 4/4D/UFL1 lysate/CHEMI_10302024_154432_(Membrane)_raw.tif]

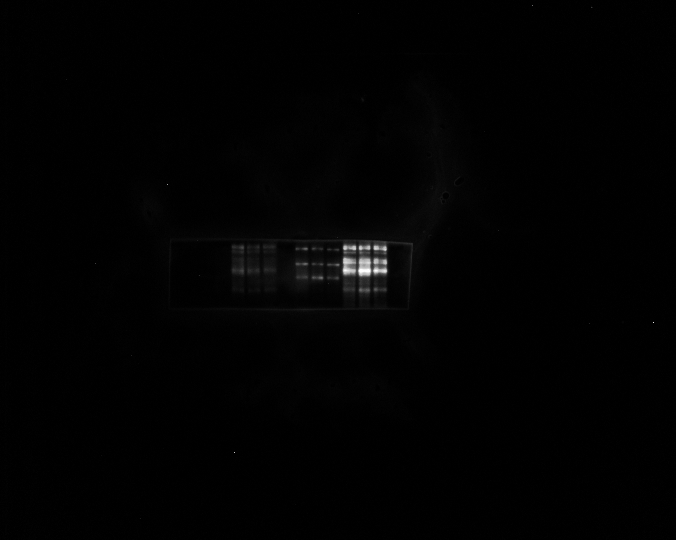

Supplement: Supplementary file 7 — Source data Fig. 4 [file 44318_2026_753_MOESM7_ESM.zip › Figure 4/4F/GFP eluate/CHEMI_10082024_151805_(Chemi)_raw.tif]

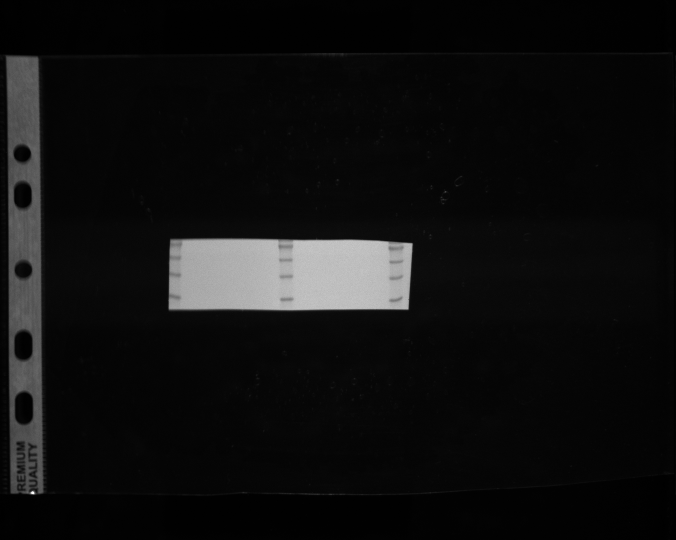

Supplement: Supplementary file 7 — Source data Fig. 4 [file 44318_2026_753_MOESM7_ESM.zip › Figure 4/4F/GFP eluate/CHEMI_10082024_151805_(Membrane)_raw.tif]

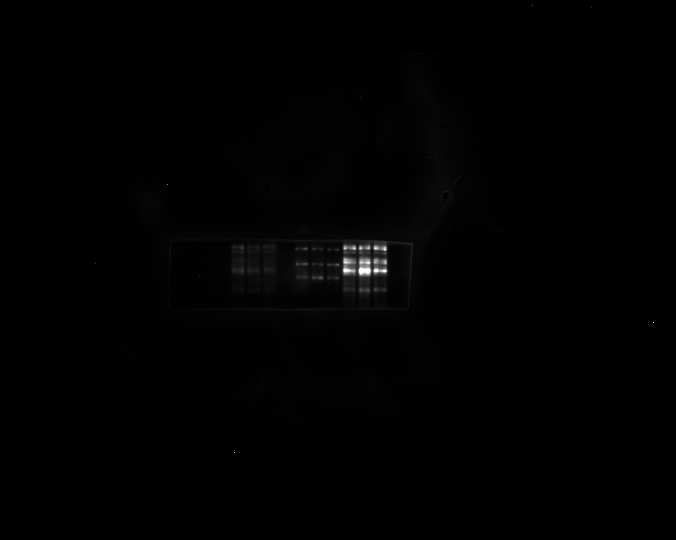

Supplement: Supplementary file 7 — Source data Fig. 4 [file 44318_2026_753_MOESM7_ESM.zip › Figure 4/4F/GFP pellet/CHEMI_10082024_151822_(Chemi)_raw.tif]

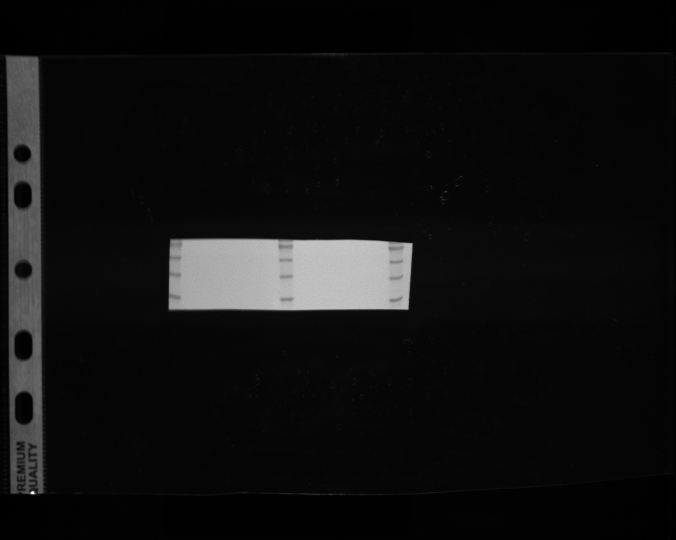

Supplement: Supplementary file 7 — Source data Fig. 4 [file 44318_2026_753_MOESM7_ESM.zip › Figure 4/4F/GFP pellet/CHEMI_10082024_151822_(Membrane)_raw.tif]

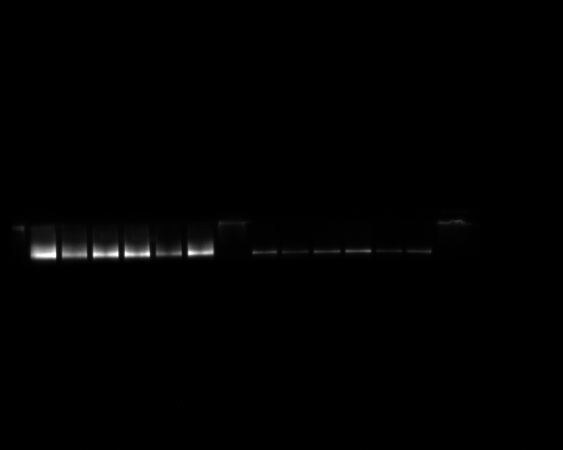

Supplement: Supplementary file 7 — Source data Fig. 4 [file 44318_2026_753_MOESM7_ESM.zip › Figure 4/4F/LTN1/CHEMI_10082024_160849_(Chemi)_raw.tif]

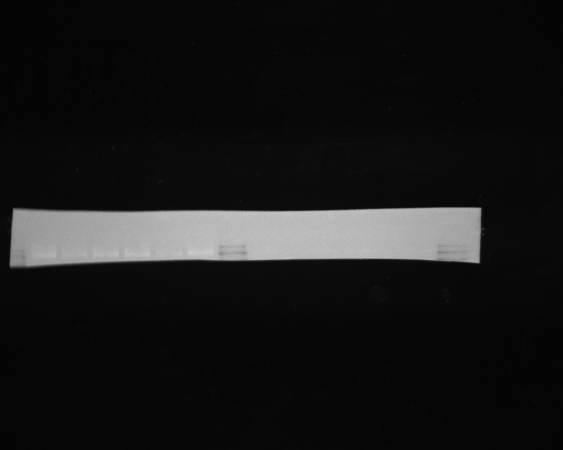

Supplement: Supplementary file 7 — Source data Fig. 4 [file 44318_2026_753_MOESM7_ESM.zip › Figure 4/4F/LTN1/CHEMI_10082024_160849_(Membrane)_raw.tif]

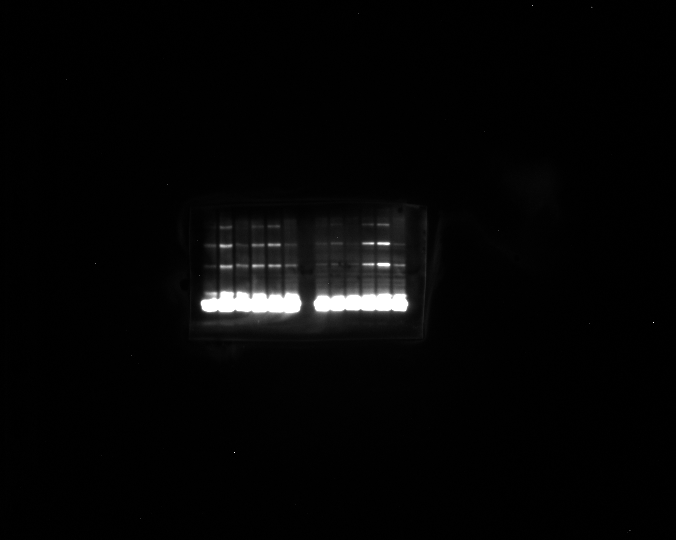

Supplement: Supplementary file 7 — Source data Fig. 4 [file 44318_2026_753_MOESM7_ESM.zip › Figure 4/4F/RPL26/High exposure/CHEMI_10082024_151529_(Chemi)_raw.tif]

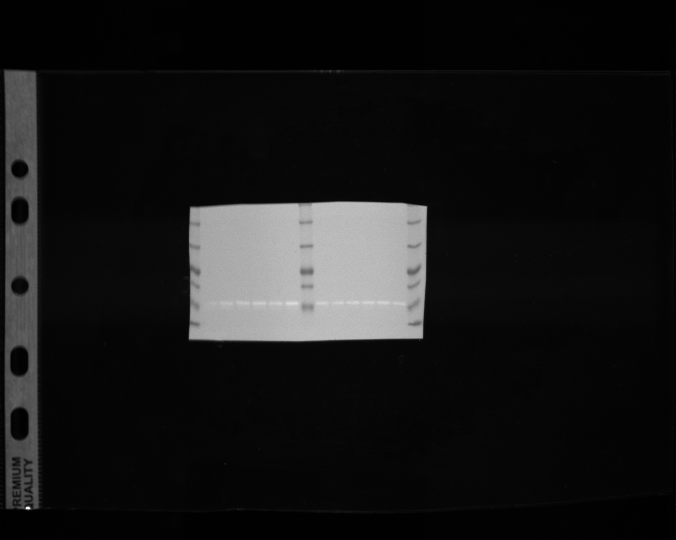

Supplement: Supplementary file 7 — Source data Fig. 4 [file 44318_2026_753_MOESM7_ESM.zip › Figure 4/4F/RPL26/High exposure/CHEMI_10082024_151529_(Membrane)_raw.tif]

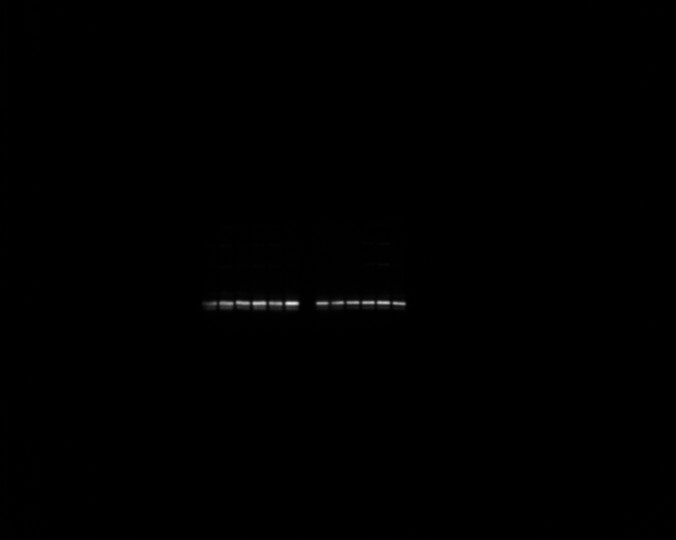

Supplement: Supplementary file 7 — Source data Fig. 4 [file 44318_2026_753_MOESM7_ESM.zip › Figure 4/4F/RPL26/Low exposure/CHEMI_10082024_151503_(Chemi)_raw.tif]

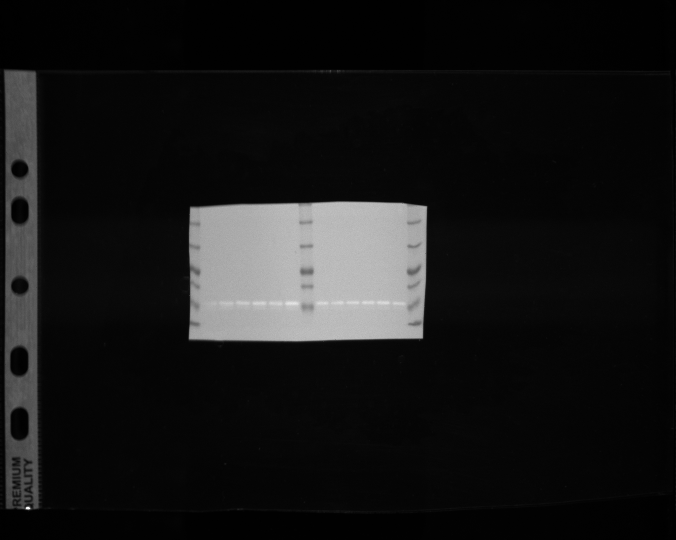

Supplement: Supplementary file 7 — Source data Fig. 4 [file 44318_2026_753_MOESM7_ESM.zip › Figure 4/4F/RPL26/Low exposure/CHEMI_10082024_151503_(Membrane)_raw.tif]

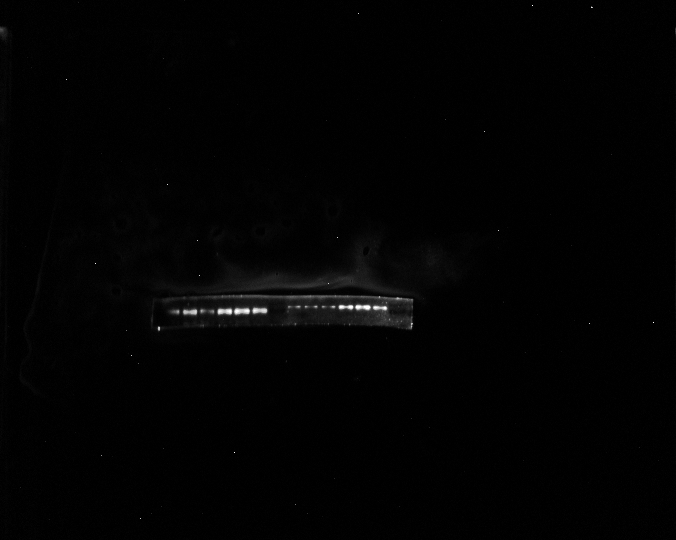

Supplement: Supplementary file 7 — Source data Fig. 4 [file 44318_2026_753_MOESM7_ESM.zip › Figure 4/4F/UFL1/CHEMI_10082024_161146_(Chemi)_raw.tif]

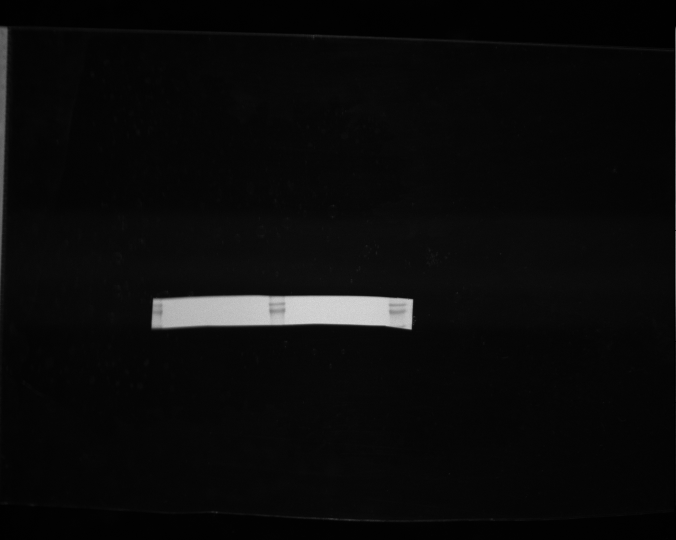

Supplement: Supplementary file 7 — Source data Fig. 4 [file 44318_2026_753_MOESM7_ESM.zip › Figure 4/4F/UFL1/CHEMI_10082024_161146_(Membrane)_raw.tif]

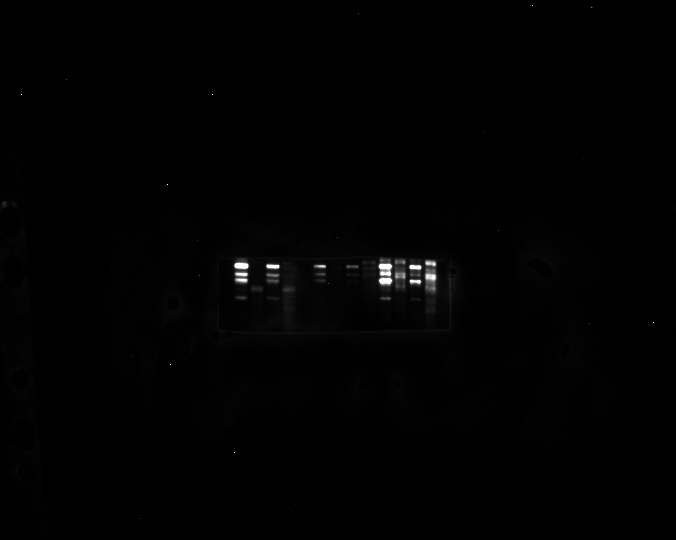

Supplement: Supplementary file 7 — Source data Fig. 4 [file 44318_2026_753_MOESM7_ESM.zip › Figure 4/4I/GFP/CHEMI_10232025_175120Chemi.tif]

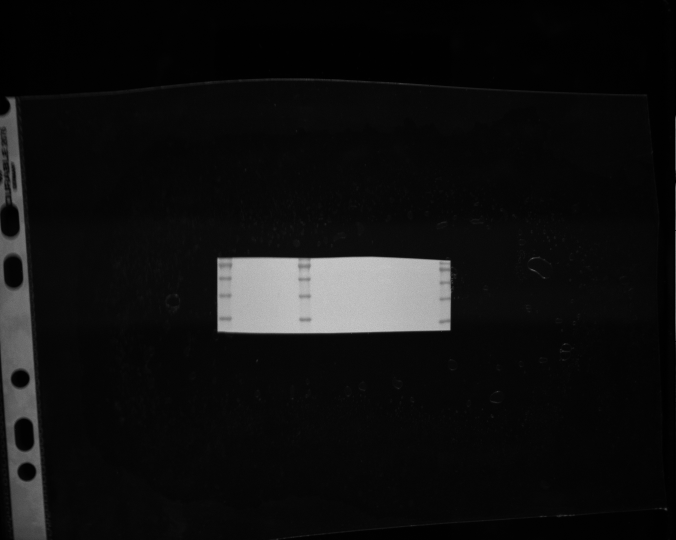

Supplement: Supplementary file 7 — Source data Fig. 4 [file 44318_2026_753_MOESM7_ESM.zip › Figure 4/4I/GFP/CHEMI_10232025_175120Membrane.tif]

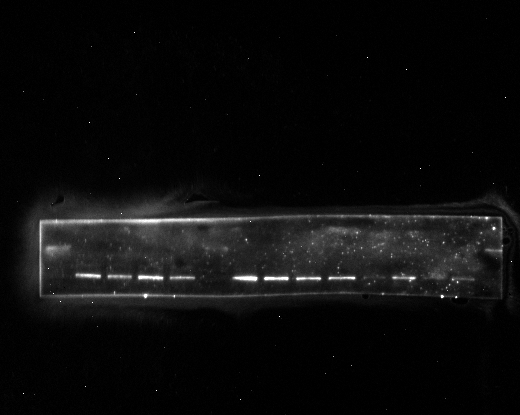

Supplement: Supplementary file 7 — Source data Fig. 4 [file 44318_2026_753_MOESM7_ESM.zip › Figure 4/4I/NEMF/CHEMI_10232025_173821Chemi.tif]

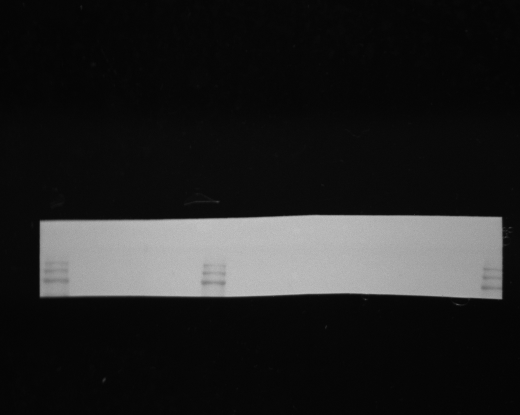

Supplement: Supplementary file 7 — Source data Fig. 4 [file 44318_2026_753_MOESM7_ESM.zip › Figure 4/4I/NEMF/CHEMI_10232025_173821Membrane.tif]

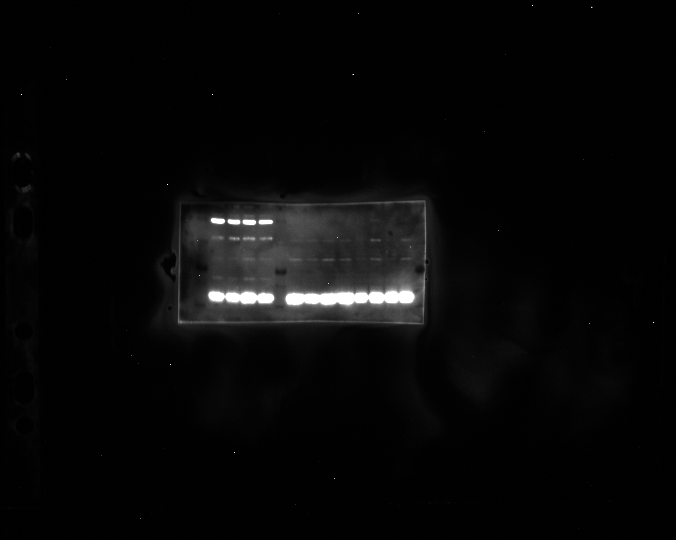

Supplement: Supplementary file 7 — Source data Fig. 4 [file 44318_2026_753_MOESM7_ESM.zip › Figure 4/4I/RPL26 high exposure/CHEMI_10232025_180325Chemi.tif]

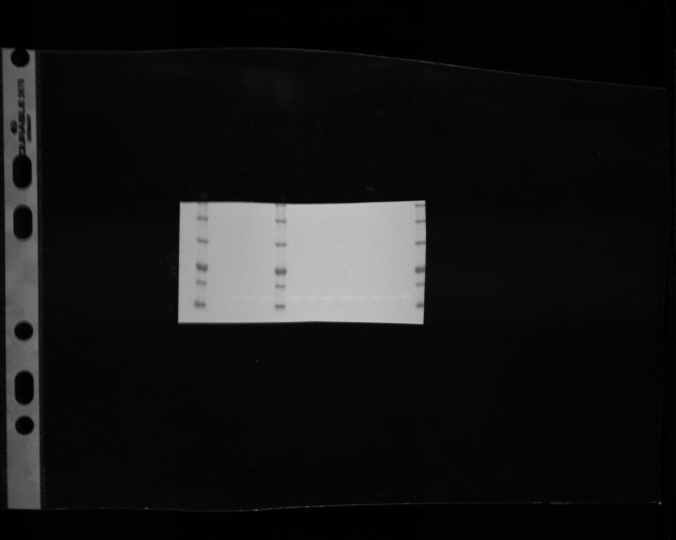

Supplement: Supplementary file 7 — Source data Fig. 4 [file 44318_2026_753_MOESM7_ESM.zip › Figure 4/4I/RPL26 high exposure/CHEMI_10232025_180325Membrane.tif]

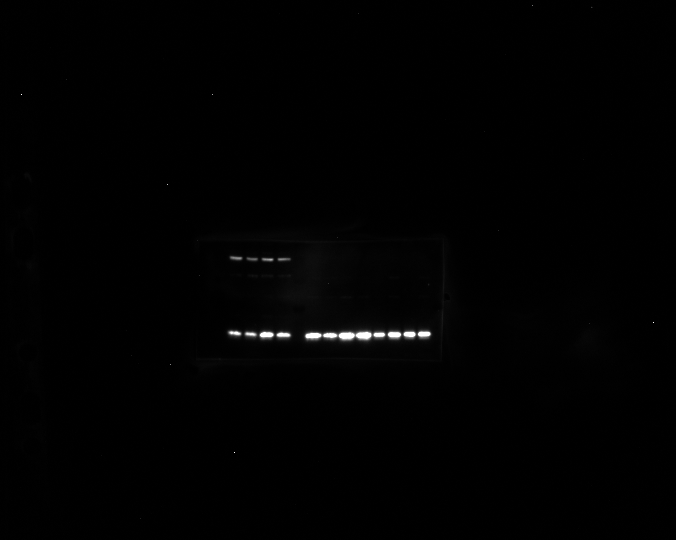

Supplement: Supplementary file 7 — Source data Fig. 4 [file 44318_2026_753_MOESM7_ESM.zip › Figure 4/4I/RPL26 low exposure/CHEMI_10232025_175636Chemi.tif]

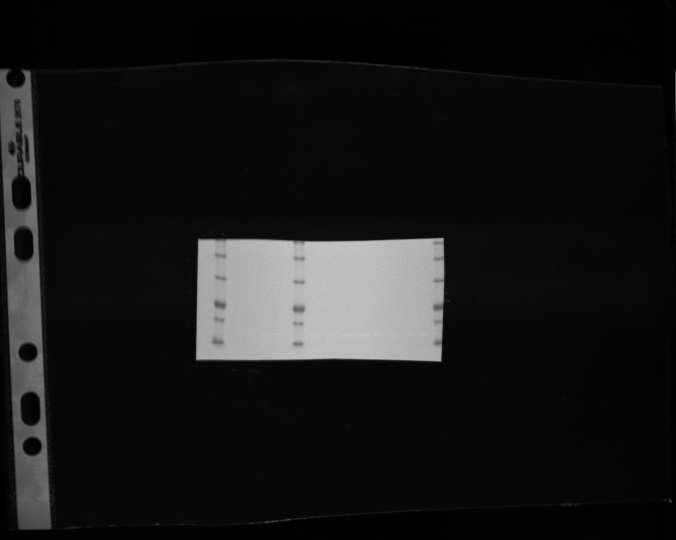

Supplement: Supplementary file 7 — Source data Fig. 4 [file 44318_2026_753_MOESM7_ESM.zip › Figure 4/4I/RPL26 low exposure/CHEMI_10232025_175636Membrane.tif]

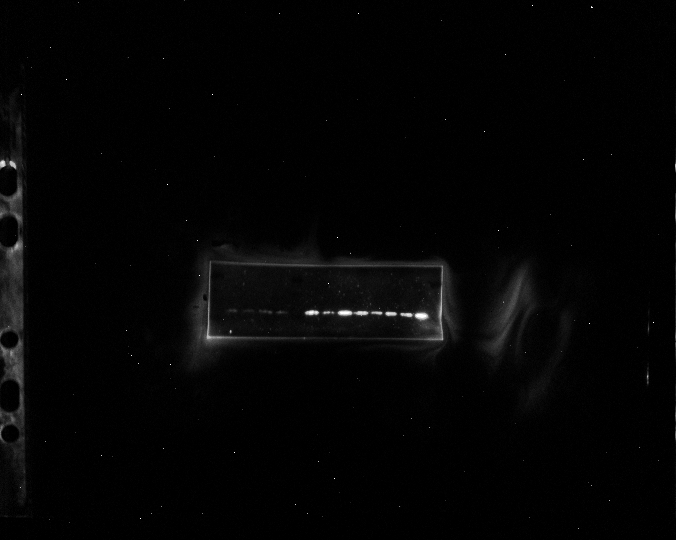

Supplement: Supplementary file 7 — Source data Fig. 4 [file 44318_2026_753_MOESM7_ESM.zip › Figure 4/4I/RPS10/CHEMI_10232025_174603Chemi.tif]

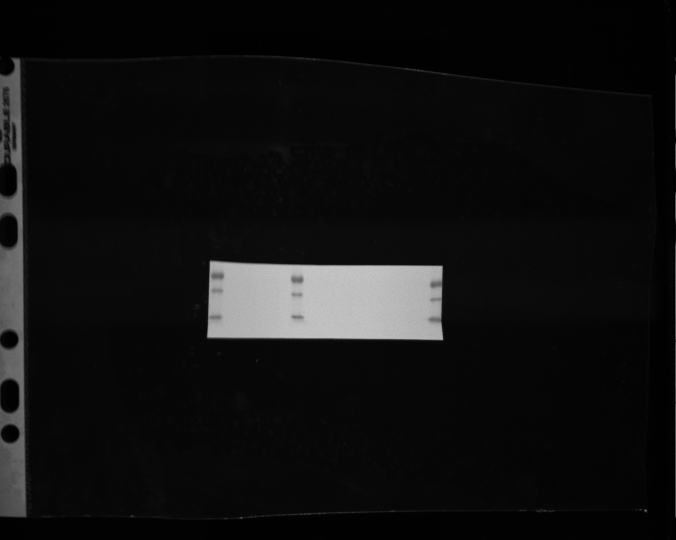

Supplement: Supplementary file 7 — Source data Fig. 4 [file 44318_2026_753_MOESM7_ESM.zip › Figure 4/4I/RPS10/CHEMI_10232025_174603Membrane.tif]

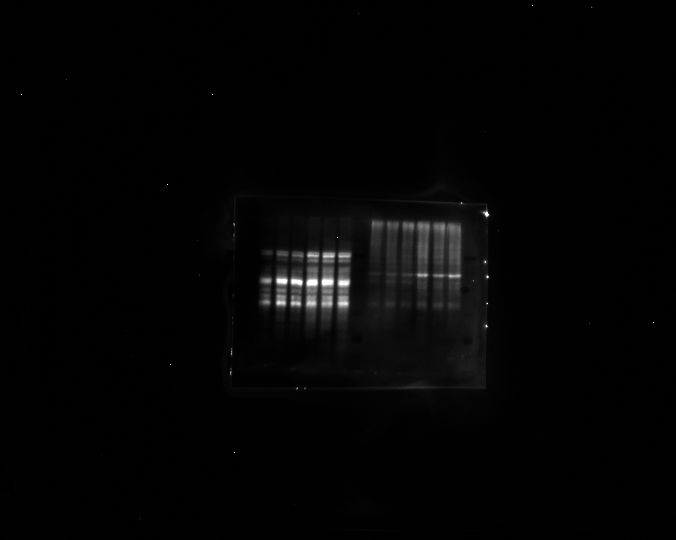

Supplement: Supplementary file 8 — Source data Fig. 5 [file 44318_2026_753_MOESM8_ESM.zip › Figure 5/5A/FLAG/CHEMI_08222025_180645Chemi.tif]

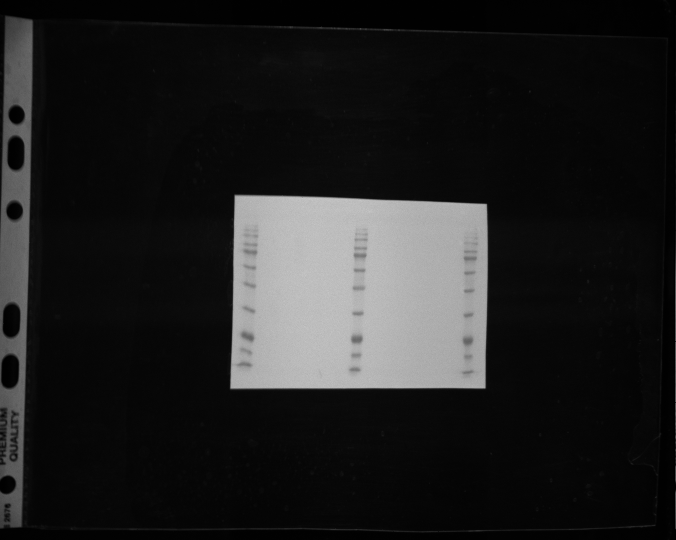

Supplement: Supplementary file 8 — Source data Fig. 5 [file 44318_2026_753_MOESM8_ESM.zip › Figure 5/5A/FLAG/CHEMI_08222025_180645Membrane.tif]

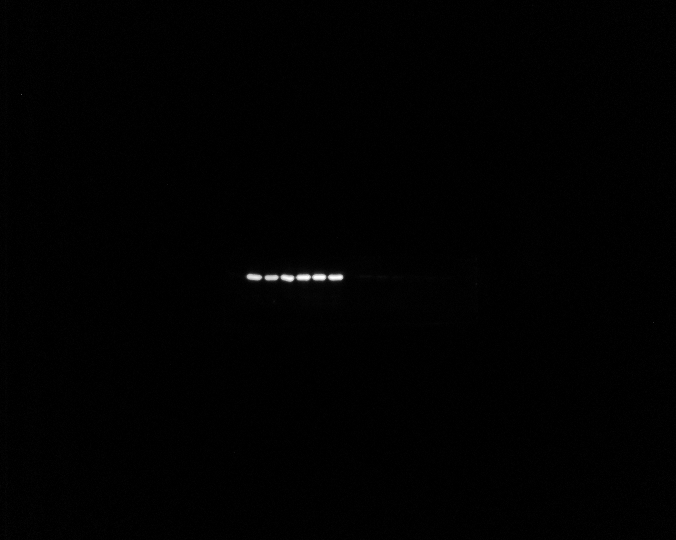

Supplement: Supplementary file 8 — Source data Fig. 5 [file 44318_2026_753_MOESM8_ESM.zip › Figure 5/5A/GAPDH/CHEMI_08222025_180920Chemi.tif]

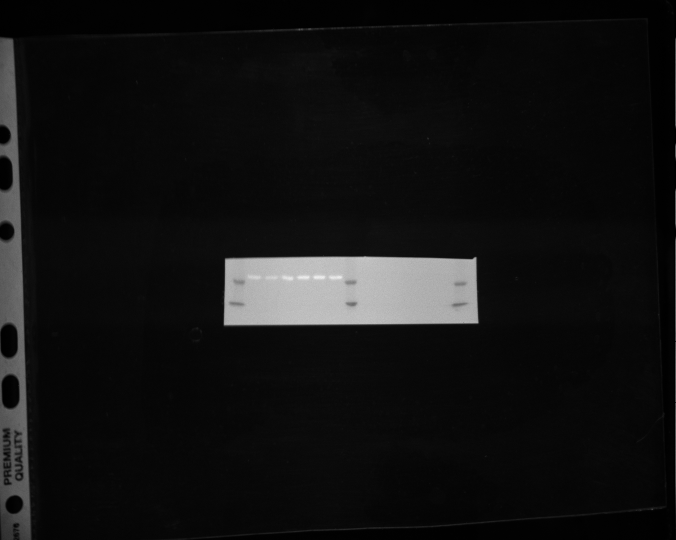

Supplement: Supplementary file 8 — Source data Fig. 5 [file 44318_2026_753_MOESM8_ESM.zip › Figure 5/5A/GAPDH/CHEMI_08222025_180920Membrane.tif]

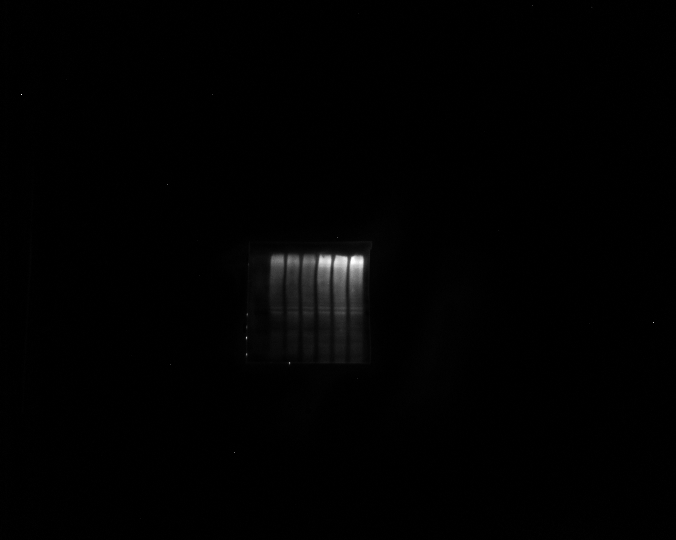

Supplement: Supplementary file 8 — Source data Fig. 5 [file 44318_2026_753_MOESM8_ESM.zip › Figure 5/5A/Ub input lysate/CHEMI_08222025_180320Chemi.tif]

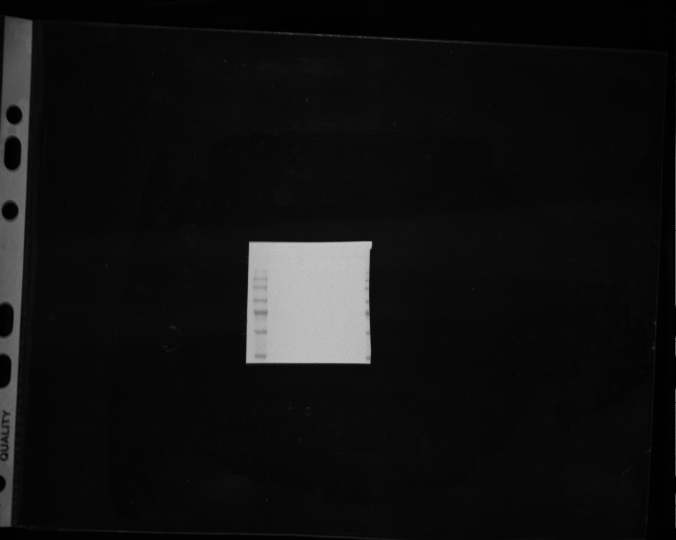

Supplement: Supplementary file 8 — Source data Fig. 5 [file 44318_2026_753_MOESM8_ESM.zip › Figure 5/5A/Ub input lysate/CHEMI_08222025_180320Membrane.tif]

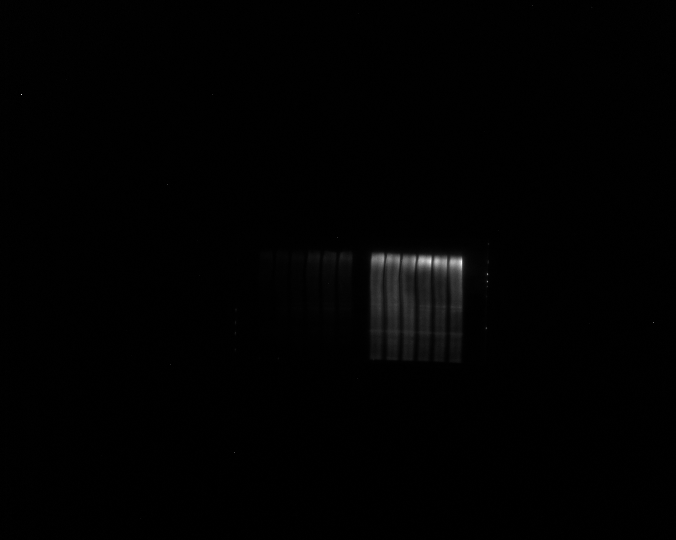

Supplement: Supplementary file 8 — Source data Fig. 5 [file 44318_2026_753_MOESM8_ESM.zip › Figure 5/5A/Ub TUBE IP/CHEMI_08222025_180019Chemi.tif]

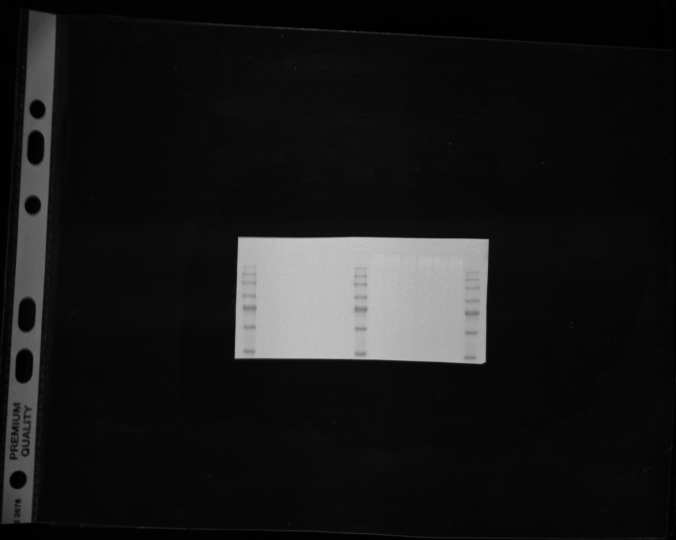

Supplement: Supplementary file 8 — Source data Fig. 5 [file 44318_2026_753_MOESM8_ESM.zip › Figure 5/5A/Ub TUBE IP/CHEMI_08222025_180019Membrane.tif]

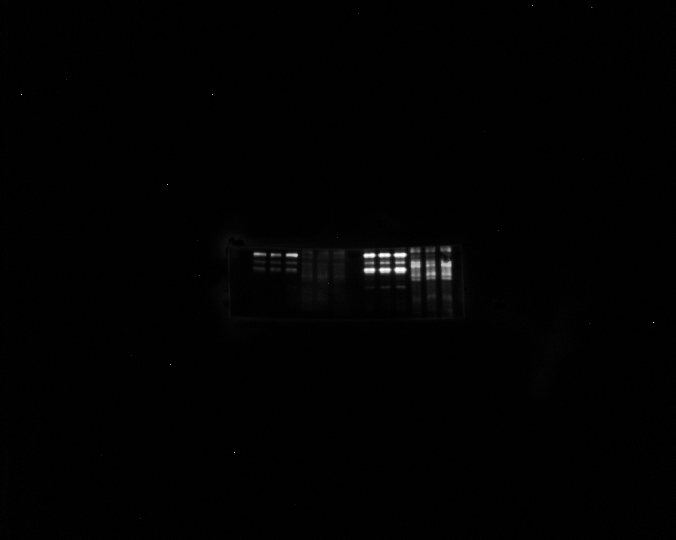

Supplement: Supplementary file 8 — Source data Fig. 5 [file 44318_2026_753_MOESM8_ESM.zip › Figure 5/5C/GFP/CHEMI_10282025_173257Chemi.tif]

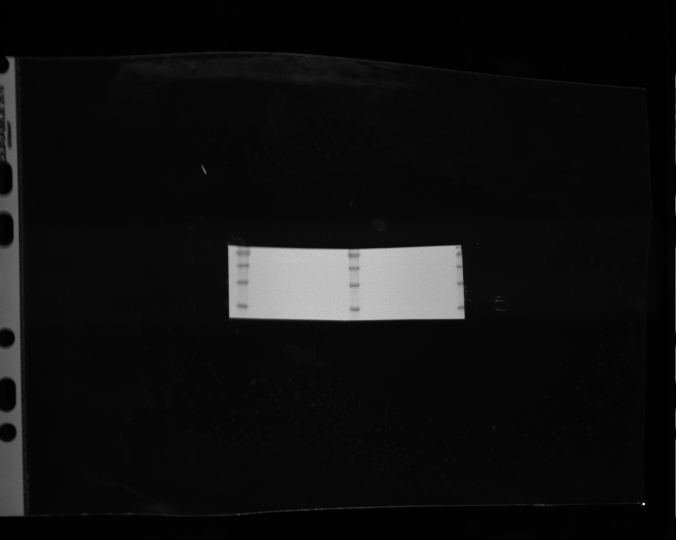

Supplement: Supplementary file 8 — Source data Fig. 5 [file 44318_2026_753_MOESM8_ESM.zip › Figure 5/5C/GFP/CHEMI_10282025_173257Membrane.tif]

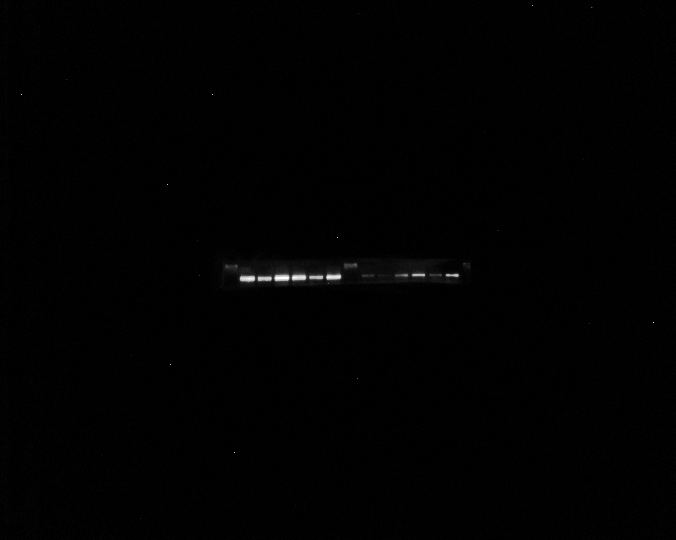

Supplement: Supplementary file 8 — Source data Fig. 5 [file 44318_2026_753_MOESM8_ESM.zip › Figure 5/5C/LTN1/CHEMI_10282025_163645Chemi.tif]

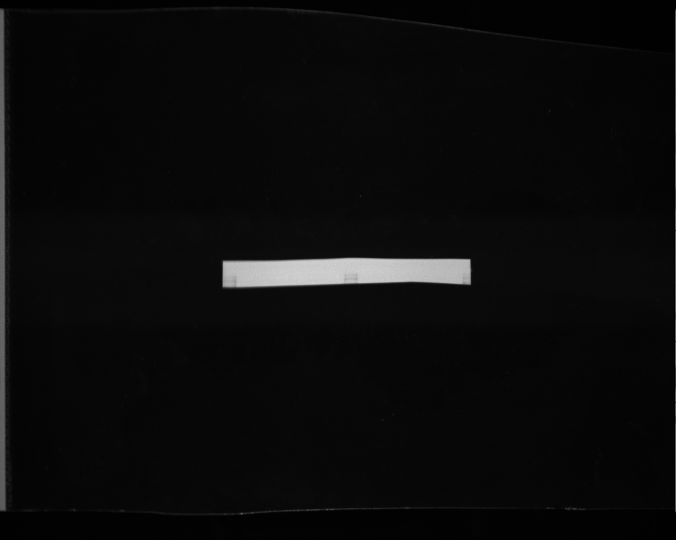

Supplement: Supplementary file 8 — Source data Fig. 5 [file 44318_2026_753_MOESM8_ESM.zip › Figure 5/5C/LTN1/CHEMI_10282025_163645Membrane.tif]

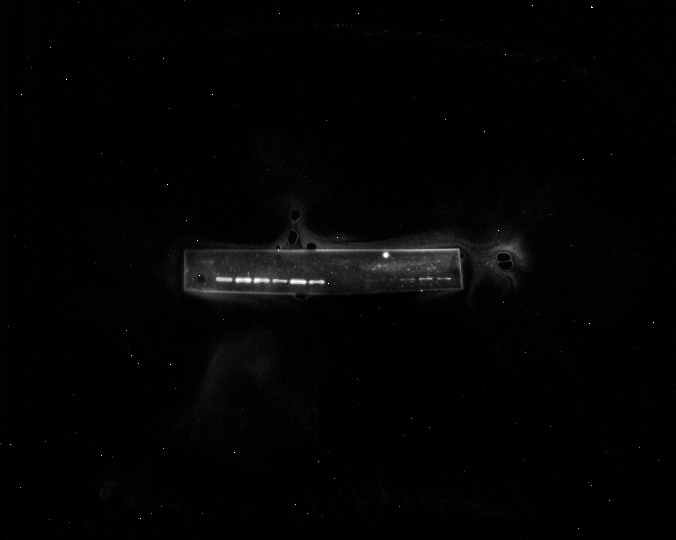

Supplement: Supplementary file 8 — Source data Fig. 5 [file 44318_2026_753_MOESM8_ESM.zip › Figure 5/5C/NEMF/CHEMI_10282025_172029Chemi.tif]

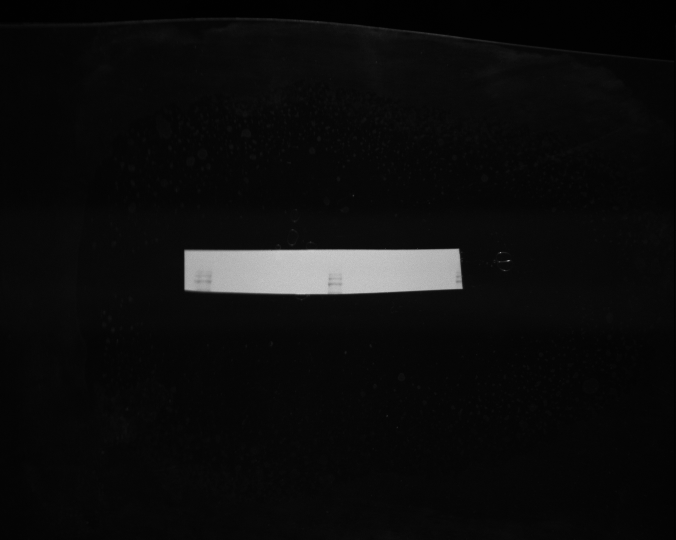

Supplement: Supplementary file 8 — Source data Fig. 5 [file 44318_2026_753_MOESM8_ESM.zip › Figure 5/5C/NEMF/CHEMI_10282025_172029Membrane.tif]

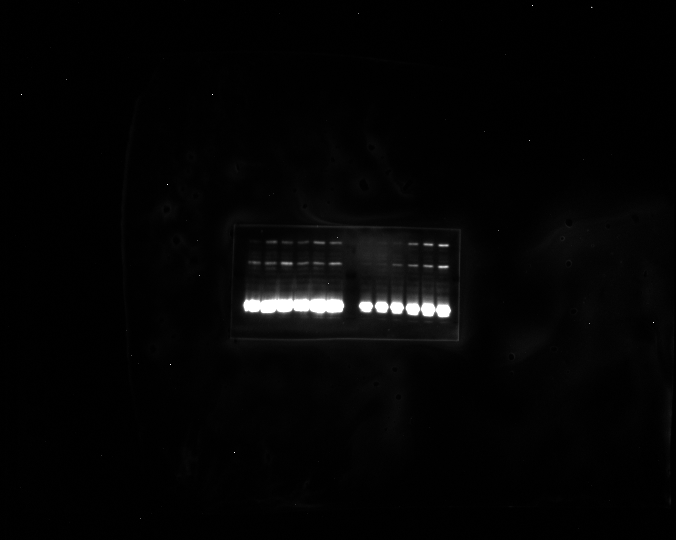

Supplement: Supplementary file 8 — Source data Fig. 5 [file 44318_2026_753_MOESM8_ESM.zip › Figure 5/5C/RPL26 high exposure/CHEMI_10282025_173725Chemi.tif]

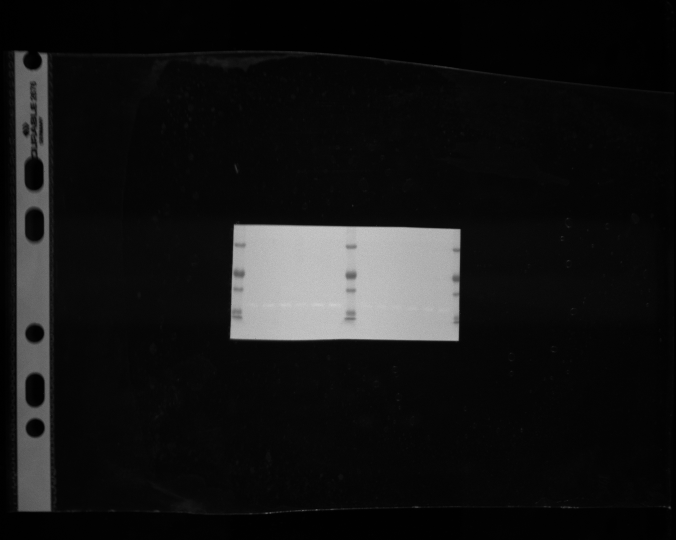

Supplement: Supplementary file 8 — Source data Fig. 5 [file 44318_2026_753_MOESM8_ESM.zip › Figure 5/5C/RPL26 high exposure/CHEMI_10282025_173725Membrane.tif]

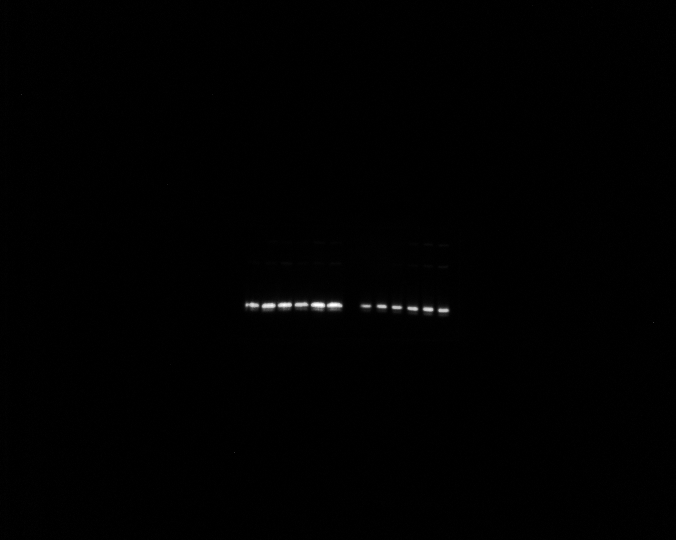

Supplement: Supplementary file 8 — Source data Fig. 5 [file 44318_2026_753_MOESM8_ESM.zip › Figure 5/5C/RPL26 low exposure/CHEMI_10282025_173549Chemi.tif]

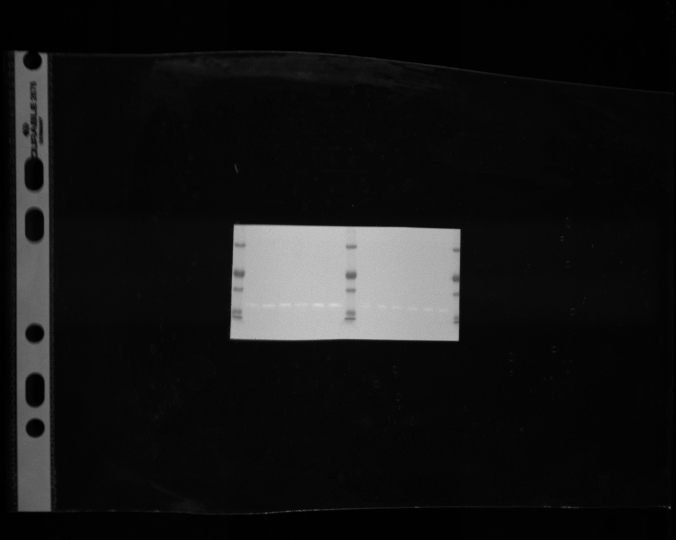

Supplement: Supplementary file 8 — Source data Fig. 5 [file 44318_2026_753_MOESM8_ESM.zip › Figure 5/5C/RPL26 low exposure/CHEMI_10282025_173549Membrane.tif]

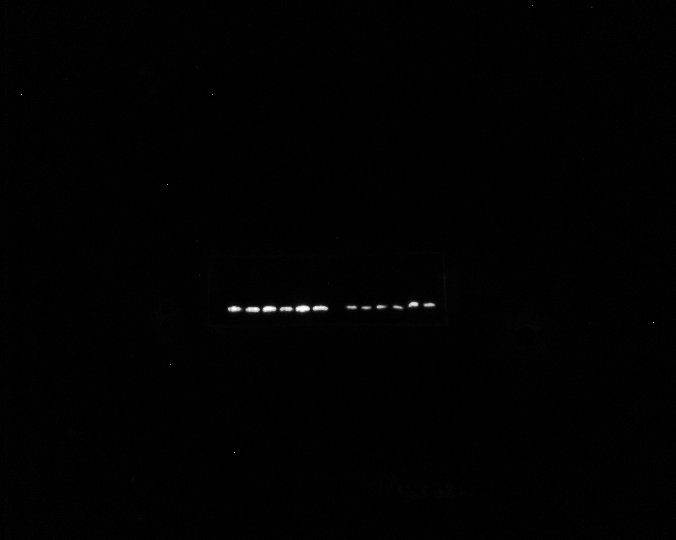

Supplement: Supplementary file 8 — Source data Fig. 5 [file 44318_2026_753_MOESM8_ESM.zip › Figure 5/5C/RPS10/CHEMI_10282025_172847Chemi.tif]

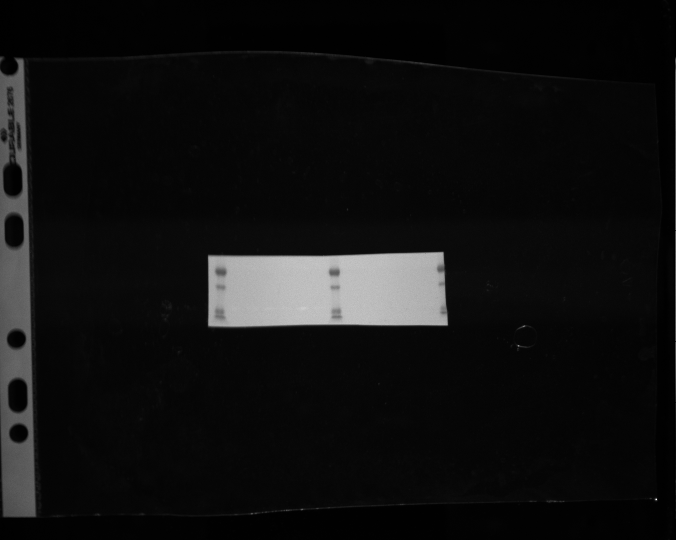

Supplement: Supplementary file 8 — Source data Fig. 5 [file 44318_2026_753_MOESM8_ESM.zip › Figure 5/5C/RPS10/CHEMI_10282025_172847Membrane.tif]
