# Supplementary material for: The coordinated action of UFMylation and the RQC pathways clears arrested polypeptides at the ER
Source: EMBO J. 2026 Mar 25;45(9):3252–75. doi: 10.1038/s44318-026-00753-9 (PMC13144351; doi:10.1038/s44318-026-00753-9)
Supplement: Supplementary file 9 — Expanded View Figures [file 44318_2026_753_MOESM9_ESM.pdf]

Expanded View Figures

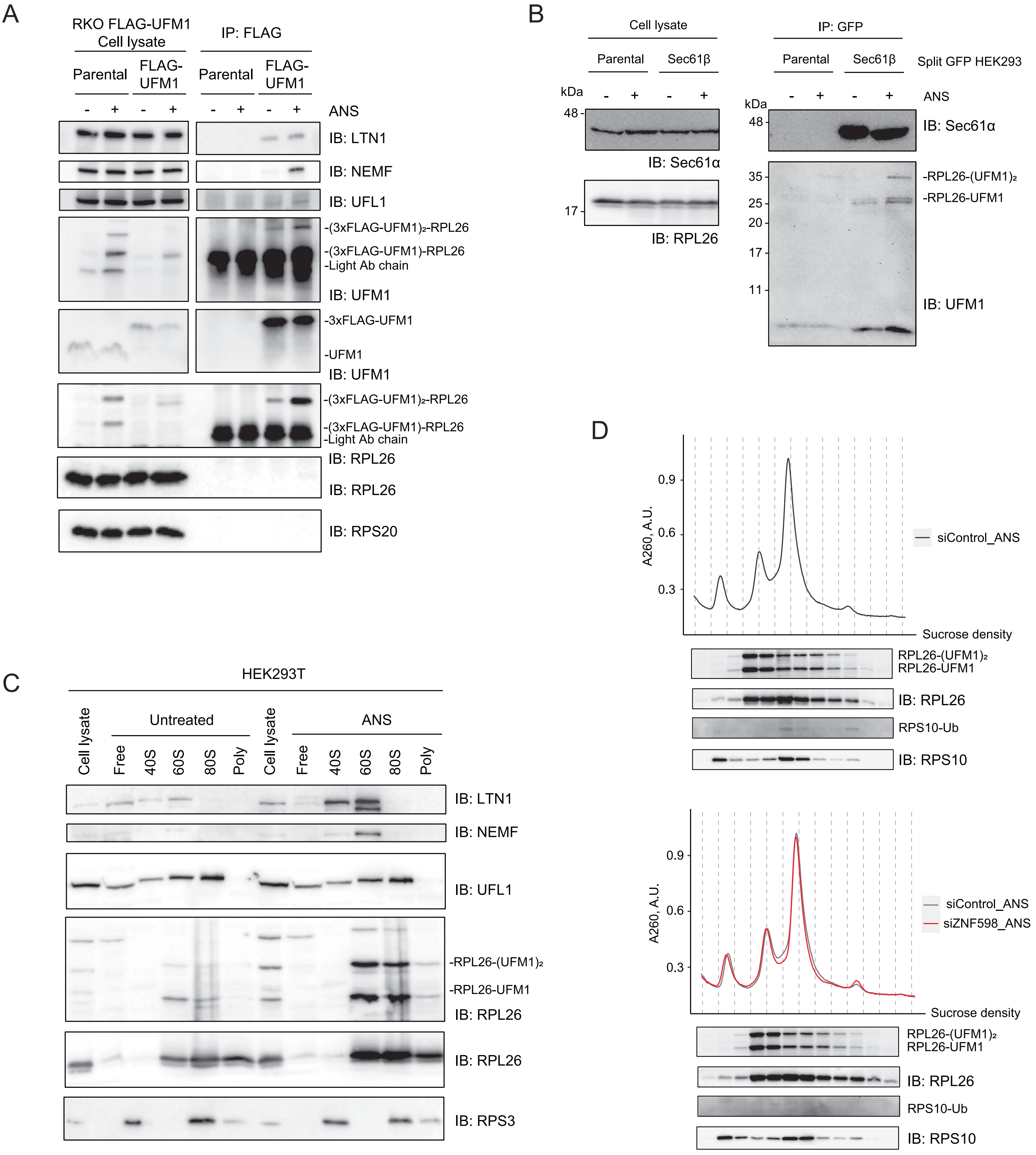

Figure EV1. The RQC and UFM1 E3 ligase machinery associate with stalled ribosomes.

(**A**) Immunoblot analysis of FLAG IP eluates upon 200 nM 1 h ANS treatment or from untreated RKO parental or RKO FLAG-UFM1 cells. (**B**) Immunoblot analysis of GFP IP eluates upon 4 μM 3 h ANS treatment or from untreated HEK293 split GFP parental or HEK293 Sec61β-split GFP cell lines. (**C**) Immunoblots of selected sucrose fractions of HEK293T cell lysates upon 200 nM 1 h ANS treatment or from untreated control condition. (**D**) Polysome profiles from HCT116 cells upon 72 h knockdown of control siRNA (top panel) or ZNF598 (bottom panel), accompanied by immunoblots showing RPL26 and RPS10 protein levels from corresponding fractions.

 © The Author(s)

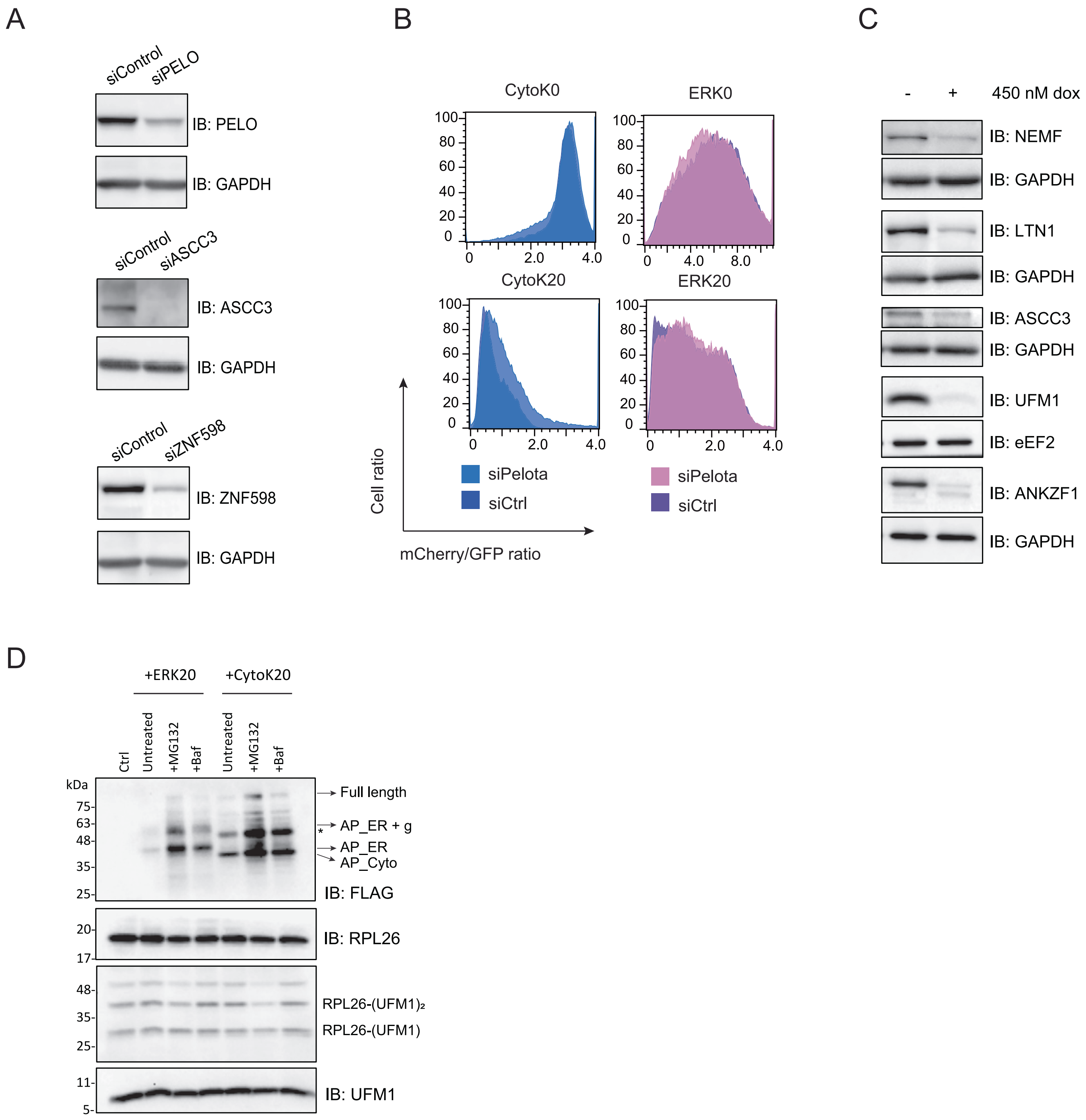

Figure EV2. The ER stalling reporter is primarily stabilized by a proteasomal inhibitor.

(**A**) Immunoblots showing knockdowns of RQC proteins Pelota, ASCC3, or ZNF598 upon 72 h siRNA treatment in HCT116 cells. GAPDH is used as a loading control. (**B**) Readthrough of reporters from Fig. 2A shown by mCherry/GFP ratio measured by FACS after 24 h expression in HCT116 cells upon siRNA-mediated knockdown of Pelota compared to non-targeting control siRNA. (**C**) Immunoblots showing knockouts of RQC proteins NEMF, LTN1, ASCC3, ANKZF1, or UFM1 knockout upon 48 h dox treatment in RKO cells. GAPDH or eEF2 are used as a loading control. (**D**) Immunoblots showing reporter accumulation upon 24 h expression of CytoK20 or ERK20 in HCT116 cells after treatment with either 20 nM MG132 for 3 h, 10 nM Baf for 16 h, or untreated as a control. AP arrested peptide, * degradation products.

© The Author(s)

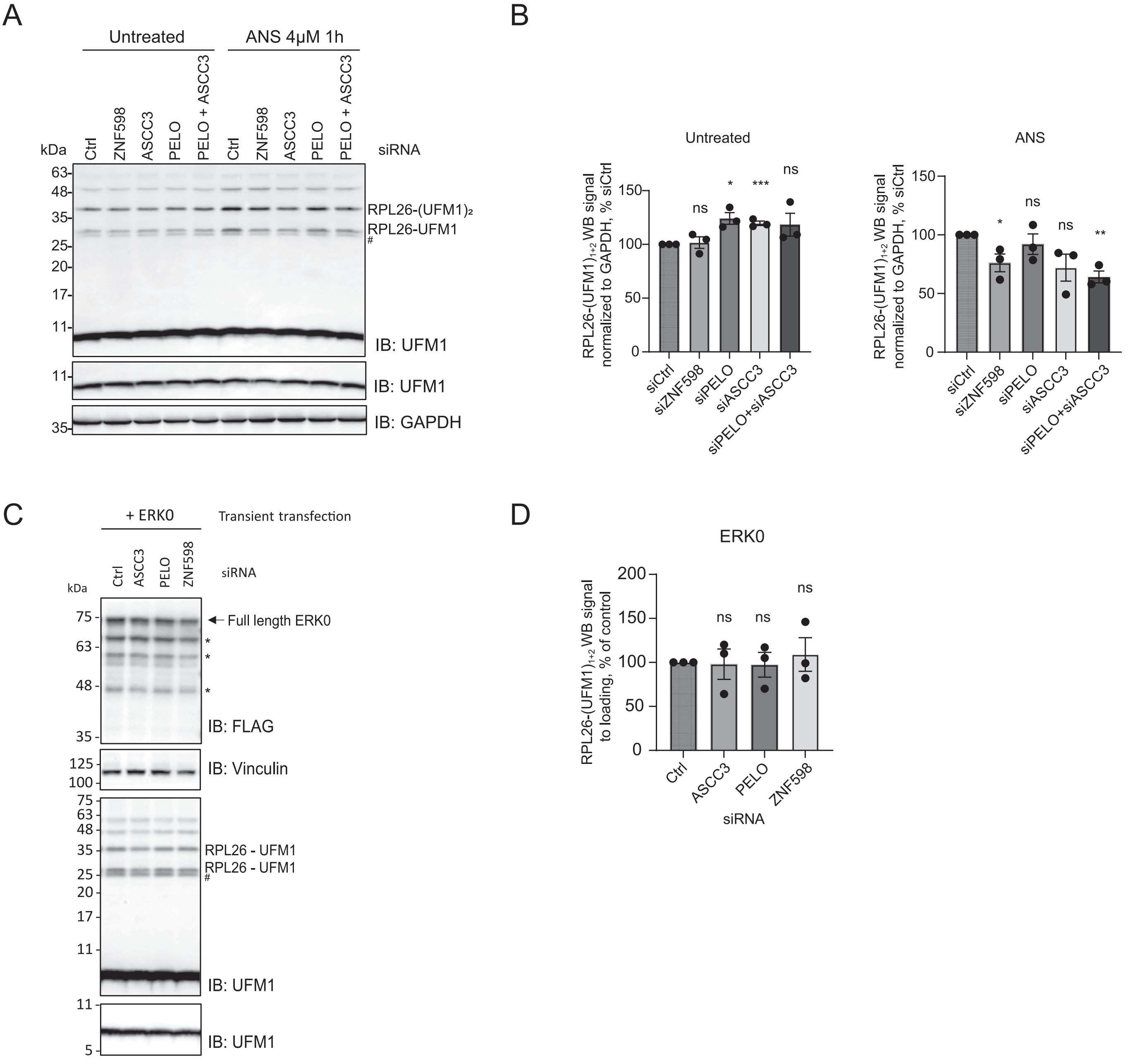

Figure EV3. Ribosomal splitting precedes UFMylation of RPL26.

(A) UFMylation levels visualized by UFM1 immunoblot in untreated or 1 h 4 μM ANS treated HCT116 cells upon 72 h siRNA-mediated knockdown of RQC components or non-targeting control. **(B)** Quantification of **(A)** with unpaired two-sided Student *t*-test, $n = 3$. Error bars represent SEM. **(C)** UFMylation levels visualized by UFM1 immunoblot in HCT116 cells upon 72 h siRNA-mediated knockdown of RQC components or non-targeting control, and 24 h expression of ERK0. **(D)** Quantification of **(C)** with unpaired two-sided Student *t*-test, $n = 3$. Error bars represent SEM. ****$P \leq 0.0001$, ***$P \leq 0.001$, **$P \leq 0.01$, *$P \leq 0.05$. List of complete *P* values available in Dataset EV2. # - UFC1-UFM1 complex, * degradation products.

 © The Author(s)

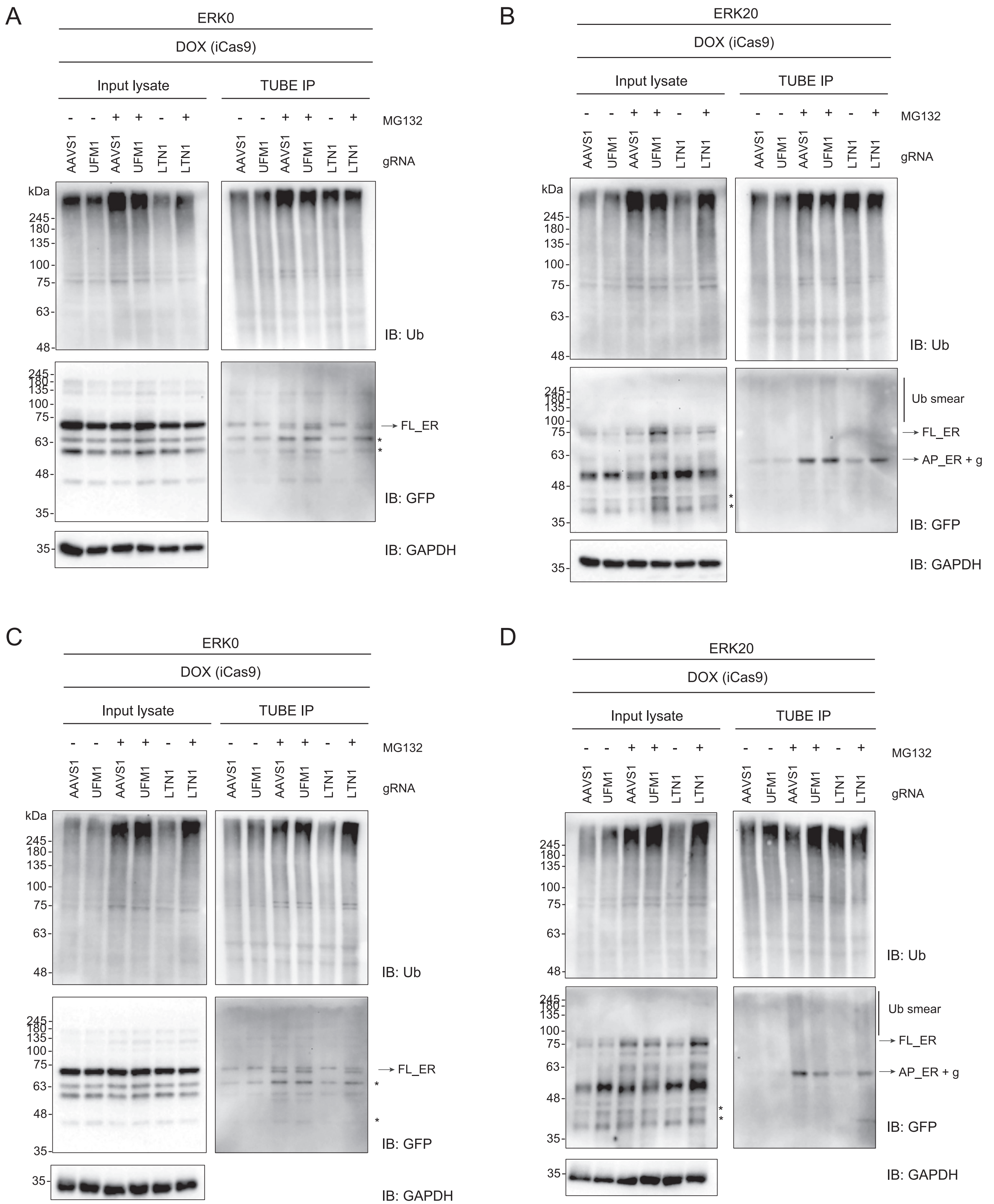
A
ERK0
DOX (iCas9)
Input lysate
TUBE IP
MG132
gRNA
AAVS1
UFM1
LTN1
kDa
245
180
135
100
75
63
48
35
IB: Ub
FL_ER
IB: GFP
IB: GAPDH
B
ERK20
Ub smear
AP_ER + g
C
D

◀ **Figure EV4. The loss of LTN1 or UFM1 does not impair the ubiquitination of ERK20.**

TUBE assays were performed from RKO iCas9 AAVS1 (non-targeting control), LTN1 or UFM1 KO cell line upon 24 h expression of ERK0 (**A**, **C**) or ERK20 reporter (**B**, **D**) and 3 h treatment with 20 µM proteasome inhibitor MG132 or DMSO as control. Protein levels are analyzed by immunoblotting. Representative duplicates. FL full length, AP arrested peptide, g glycosylation, * degradation products.

 © The Author(s)
